# Epigenetic regulation of gene expression improves Fusarium head blight resistance in durum wheat

**DOI:** 10.1038/s41598-020-73521-2

**Published:** 2020-10-19

**Authors:** Jitendra Kumar, Krishan M. Rai, Seyedmostafa Pirseyedi, Elias M. Elias, Steven Xu, Ruth Dill-Macky, Shahryar F. Kianian

**Affiliations:** 1 Department of Plant Pathology, University of Minnesota, St. Paul, MN USA; 2 Department of Microbial and Plant Genetics, University of Minnesota, St. Paul, MN USA; 3 Department of Plant Sciences, North Dakota State University, Fargo, ND USA; 4 USDA-ARS Cereal Crops Research Unit, Edward T. Schafer Agricultural Research Center, Fargo, ND USA; 5 USDA-ARS Cereal Disease Laboratory, St. Paul, MN USA

**Keywords:** Plant sciences, Plant biotechnology, Plant breeding, Plant cell biology, Plant development, Plant genetics, Plant immunity, Plant molecular biology, Plant stress responses

## Abstract

Eight advanced durum-breeding lines were treated with 5-methyl-azacytidine to test the feasibility of generating sources of Fusarium head blight (FHB) resistance. Of the 800 treated seeds, 415 germinated and were advanced up to four (M4) generations by selfing. Thirty-two of the resulting 415 M4 lines were selected following preliminary screening and were further tested for FHB resistance for three years at two field locations, and in the greenhouse. Five of the 32 M4 lines showed less than 30% disease severity, as compared to the parental lines and susceptible checks. Fusarium-damaged kernels and deoxynivalenol analyses supported the findings of the field and greenhouse disease assessments. Two of the most resistant M4 lines were crossed to a susceptible parent, advanced to third generation (BC_1_:F_3_) and were tested for stability and inheritance of the resistance. About, one third of the BC_1_:F_3_ lines showed FHB resistance similar to their M4 parents. The overall methylation levels (%) were compared using FASTmC method, which did not show a significant difference between M4 and parental lines. However, transcriptome analysis of one M4 line revealed significant number of differentially expressed genes related to biosynthesis of secondary metabolites, MAPK signaling, photosynthesis, starch and sucrose metabolism, plant hormone signal transduction and plant-pathogen interaction pathways, which may have helped in improved FHB resistance.

## Introduction

Cereals, such as maize (*Zea mays*), rice (*Oryza sativa*) and wheat (*Triticum aestivum* L.), supply approximately 45% of the dietary energy worldwide (https://www.fao.org/3/CA1796EN/ca1796en.pdf). Among them, wheat is the most important food grain source (https://www.fao.org/assets/infographics/FAO-Infographic-wheat-en.pdf). Fusarium head blight (FHB) primarily caused by *Fusarium graminearum*, is a devastating disease of both durum (*T. turgidum* L.) and common wheat (*T. aestivum* L.)^1–4^. FHB has been a significant threat to wheat production due to frequent outbreaks in many wheat-growing regions, including the United States^3,4^. The estimated losses to FHB were reported to be in excess of 100 million bushels annually for 1991 and the six subsequent years in the United States^5^. In a recent study, FHB induced yield losses in the United States were estimated around $1.176 billion during 2015 and 2016^6^. In addition to yield losses, food safety is compromised by contamination with mycotoxins, including deoxynivalenol (DON), that present a serious threat to human and animal health^7^.

The most prevalent strategies to control FHB include the application of fungicides, crop rotation and breeding for resistance^8^. Breeding for host resistance is considered as the most effective method to generate FHB resistance; however the FHB resistance is a complex trait and is controlled by multiple genes and also influenced by environmental factors^9^. Multiple sources of resistance have been identified and successfully utilized in developing FHB-resistance in bread wheat^10^. However, the plant breeding strategies have only been partly successful due to the lack of resistance sources in durum wheat^11–14^. Efforts have been made to introduce new sources of resistance from wild tetraploid wheat, hexaploid bread wheat and other alien species; however, these attempts have had only limited success^15,16^.

DNA methylation is important for plant growth and development^17^. Inadequate DNA methylation levels may result in abnormal growth and phenotype^18,19^. Plants can quickly adapt to changing environments by adjusting DNA methylation patterns^20^. For example, salt and low temperature treatments induced demethylation in the promoter region of *NtGPDL* (glycerophosphodiesterase-like protein) gene leading to stress tolerance in tobacco^21^. Transposable elements (TEs) were demethylated and transcriptionally reactivated to regulate neighboring genes as a defense response in Arabidopsis^22^. Further, demethylation of *metl* gene led to specific expression of stress response genes^23^, confirming that alteration in DNA methylation play crucial roles in plant response to environmental stresses. In another study, hypo-methylated mutants of Arabidopsis displayed enhanced resistance to the biotrophic pathogen *Hyaloperonospora arabidopsidis*, whereas two hyper-methylated mutants were susceptible^24^. Le et al.^25^ showed involvement of the DNA demethylases, ROS1, DML2, and DML3, in resistance to *Fusarium oxysporum* in Arabidopsis.

Cytosine methylation is known to be involved in many important biological processes, including defense response^26^. Cytosine methylations in the plant genome include CG, CHG, and CHH (where H denotes either A, T, or C) sequences^27^. In a study, treatment of susceptible rice plants with 5-azadeoxycytidine, which is an effective inhibitor of methyltransferase^28,29^, resulted in high levels of resistance to *Xanthomonas oryzae* and analyses indicated a complete lack of methylation in the promoter region of the *Xa21G* gene of the resistant plants^30^. The present study aimed to remove cytosine DNA methylation in advanced durum lines to test the feasibility of generating a novel source of FHB resistance. The treated lines showed promising results as compared with the parental lines and FHB-susceptible checks. A number of FHB responsive genes have been uncovered and their possible roles have been discussed in the present study.

## Materials and methods

### Select germplasm and treatment with DNA methylation inhibitor

Eight advanced durum lines; D0-3028, D0-3708, D0-41708, D0-4581, D0-6710, D0-6855, E-25 and TRT-4 (D0-3028 and D0-4581 were later released as Carpio and Joppa;^31,32^) were selected for treatment with 5-azacytidine (Millipore Sigma), which is an effective inhibitor of methyltransferase^28,29^. A total of 100 seeds for each of the eight durum lines were placed in individual Petri dishes with 0.5 mM 5-azacytidine solutions for 3 days as described by Akimoto et al.^30^. Following treatment, seeds were washed with 10% detergent and sterilized double distilled water. The treated seeds were planted individually in plastic pots containing 30% soil and 70% Pro-Line C/B Growing Mix (https://jollygardener.com/soil_jg_mixes.htm). Upon germination, the pots containing plantlets were transferred to a greenhouse set at 25 °C under 16 h photoperiod and grown to full maturity. The plants were selfed and advanced for four generations (M4) without selection (Supplementary Fig. S1). The M4 seeds were bulked and used in all the experiments.

### Experiment design and inoculations

A total of 415 M4 lines were planted in the greenhouse for elimination screening and lines that showed more than 50% susceptibility were eliminated. Of the 415 M4 lines tested, 32 were selected following elimination screening and used for multi-year multi-location field testing. The 32 M4 lines, eight parental lines and two checks, one susceptible (MN00269) and one resistant (Alsen), were planted in the mist-irrigated field nurseries with a randomized complete block design with three replicates each at two locations, Saint Paul, Minnesota (MN) and Fargo, North Dakota (ND) during the summer of 2015. An additional two years (2016 and 2017) of field evaluations were performed at Saint Paul. Lines at Saint Paul were grown in single row plots (ca. 5 feet long) and were spray inoculated and mist-irrigated to facilitate disease development as described by Guatam and Dill-Macky^33^. The inoculum in Saint Paul consisted of macroconidia from a mixture of 15–30 *F. graminearum* isolates each year. Each line in the experiment at Fargo was planted in a hill plot. The Fargo nursery was inoculated when the majority of the plants were at the boot stage by applying Fusarium-colonized corn seeds at a rate of 35.6 g m^−2^. The corn was soaked to imbibe water, autoclaved, and infected with spores produced from 20 *F. graminearum* strains. The strains used in each nursery collected from commercial wheat fields in Minnesota and North Dakota and used in that state’s screening nursery^34^.

Disease evaluations were also conducted in the greenhouse, using the concentration of 100,000 conidia/ml, with the goal of measuring Type II resistance (resistance to spread in the spike). In greenhouse experiments, the same 42 lines evaluated in the field were evaluated for four experiments conducted in Fall 2015, Spring 2016, Fall 2016 and Spring 2017. The 42 lines examined in the field experiments were included in each greenhouse experiment. Seeds of each line were planted in a plastic pot with five seeds planted for each replicate and each experiment included three replications. Twelve to 15 plants per lines were inoculated and assessed for disease development in each experiment. The greenhouse settings for photoperiod and temperature were 16 h light and 22 °C, respectively. A single virulent isolate of *F. graminearum* was used for inoculum in each of these experiments. Approximately, 100 μL of inoculum was pipetted into two adjacent spikelets (the fourth and fifth spikes from the bottom of the spike) at anthesis. Each inoculated spike was then misted with water and covered with a plastic bag for 48 h.

### Disease and mycotoxin analysis

FHB severity was examined visually 21 DAI by counting the total and the number of symptomatic spikelets in nondestructively selected 20 heads in each plot. Disease severity for each line was calculated as the percentage of infected spikelets of all spikelets assessed. Spikes (ca. 30 per plot) were harvested at maturity and dried for 5 days at 95 °C. Dried spikes were threshed using a belt thresher and the grain cleaned manually. Fusarium damaged kernels (FDK) analysis was done by counting out 100 arbitrarily selected seeds per plot and visually categorizing each grain as healthy or symptomatic, with FDK being expressed as a percentage. Following the FDK analysis, the samples were submitted for deoxynivalenol (DON) analyses using gas chromatography—mass spectrometry (GC–MS, detection limit 0.05 ppm) as described elsewhere^35^.

In the greenhouse experiments spikes (ca. 15 per entry) were assessed visually by counting the total and the number of symptomatic spikelets in each inoculated spike.

### Statistical analyses

The FHB severity data were analyzed using R, version 3.2.2 (https://www.r-project.org/). Data from the two locations, Saint Paul and Fargo, were analyzed separately by using 80% trimmed mean to reduce the effect of outliers. Separate ANOVA models were applied to determine which model resulted in more variation among the lines compared to within the lines.

### Stability and inheritance of resistance

The stability and inheritance of resistances, which were generated by alterations in the methylation patterns, were examined in two of the most promising lines. These lines were crossed with a susceptible parental cultivar; Ben^36^ and the resulting backcross-derived lines were advanced for three generations (BC_1_:F_3_) without a selection pressure. The BC_1_:F_3_ families were then tested in the field in one year and in one greenhouse experiment. These lines were tested together with the resistant M4 (parent) lines and susceptible checks.

### DNA methylation level sample preparation

A total of 10 lines, comprising of four best and three worst performing lines and their parental lines, were selected for genome wide DNA methylome level analysis using the FAST^m^C method^37^. Total genomic DNA was isolated from the selected lines using DNeasy Plant Mini Kit (Qiagen). One microgram of DNA from each sample was used for estimation of genome wide DNA methylation level as described previously^37,38^.

### Transcriptome analysis

To capture the transcriptome changes in the resistant (M4) vs. susceptible (parental) wheat varieties in response to *F. graminearum* infection, RNA-seq analysis was performed. Two time points, 12 and 48 h post inoculation (hpi), were selected to identify the differentially expressed genes (DEG) of *F. graminearum*-wheat interaction. These time points were chosen as at 12 hpi fungal spores germinate on the inner surface of lemma and palea whereas, at 48 hpi hyphae start to multiply extensively in the lemma. A total of 18 samples (6 sets of triplicates) were collected and used for RNA-seq analysis.

#### Plant growth conditions

One best performing M4 line, E.25.10, and a susceptible parent of E.25, Ben, were selected for transcriptome analysis. Seeds for both lines were sown in plastic pots containing 30% soil and 70% Pro-Line C/B Growing Mix (https://jollygardener.com/soil_jg_mixes.htm) and were grown in environment-controlled growth chambers set at 22˚C/20˚C (day/night cycle) with a 16 h photoperiod. Plants were fertilized once, applied two weeks after planting with, 20-20-20 (N-P-K).

#### *Fusarium* inoculations

Strain PH-1 of *F. graminearum* (provided by Dr. H Corby Kistler, USDA-ARS Cereal Disease Laboratory) was used for the inoculations of the plants used in the transcriptome analysis. Conidia were produced in CarboxyMethyl Cellulose (CMC) liquid medium by incubating in a shaker at 28 °C, 180 rpm for 2 days and harvested as described elsewhere^39^. Eight E.25.10 plants and eight Ben plants were inoculated with macroconidial inoculum and four E.25.10 plants and four Ben plants, were mock-inoculated with water. At mid-anthesis, 2–3 florets in a single spike of a biological replicate were inoculated by pipetting conidial suspension between the palea and lemma. Immediately following the inoculation the inoculated spikes were misted with water and covered with a plastic bag to promote disease development. Mock-inoculated plants were also misted with water and covered with a plastic bag. Of the eight E.25.10 and eight Ben plants, four plants were sampled at 12 hpi and four at 48 hpi. Infected spikelets from each of the four biological replicates were harvested separately at 12 and 48 hpi. Inoculated spikelets were also collected from the each of the four biological replicates of the mock-inoculated plants at 48 hpi. Harvested spikelets were frozen immediately in liquid nitrogen and stored in − 80 °C freezer for further analysis.

#### RNA sequencing and data processing

Three biological replicates of each genotype and treatment were used for transcriptome analysis. Total RNA was harvested from the infected spikelets at 12 and 48 hpi and also from the mock-inoculated spikelets using Spectrum Plant Total RNA Kit (Millipore Sigma) following manufacturer’s instructions. On column DNase digestion protocol (Millipore Sigma) was performed to remove any residual DNA during the RNA isolation. Three biological replicates for each genotype and treatment were used for transcriptome analysis. TruSeq dual indexed stranded RNA libraries were prepared following the manufacturers guidelines (Illumina). RNA quality and library size were analyzed on a Bioanalyzer (Agilent Technologies). Libraries were sequenced on an Illumina Genome Analyzer using HiSeq 2500 High Output, 50 bp PE flow cell and v4 chemistry at the University of Minnesota Genomics Center. The sequencing files were submitted to NCBI following preliminary analysis (SRA accession PRJNA595999).

Raw data was processed, using the rnaseq2 pipeline available at the gopher-pipelines (https://bitbucket.org/jgarbe/gopher-pipelines/wiki/rnaseq2-pipeline), for quality filtration, adapter trimming and reads mapping using Kallisto. The mapping result output was used for downstream analysis.

#### Differential gene expression analysis

Normalization and differential expression analyses were performed using DESeq2^40^. After calling for differentially expressed genes (DEGs), the normalized data along with log2 fold changes and p-values ≤ 0.01, were used for downstream analysis. Genes differentially expressed with log2 fold change ≥ 2 or ≤ − 2 and a P ≤ 0.01 were considered significant. For the identification of DEGs, the DESeq cutoff was set to 0.5 RPKM while the DESeq parameters for dispersion estimation were set with method “pooled” and sharing Mode “fitOnly”. The false discovery rate (FDR) threshold for DEG calling was set to 0.05. DEGs common to the mock-inoculated and *Fusarium*-inoculated were removed from the further analysis. Common and unique DEGs between the inoculated treatment samples harvested at 12 and 48 hpi were used for various annotation purposes.

#### Gene Ontology annotations and enrichment analysis

For the Gene Ontology (GO) analysis, gene ids for each DEGs were identified using the blastP similarity search with e-value of 10^–5^ in the corresponding *Triticum aestivum* database (https://phytozome.jgi.doe.gov/pz/portal.html#!info?alias=Org_Taestivum_er). GO enrichment analysis was performed using agriGO^41^. MapMan analysis was performed with default parameters to assign MapMan bins for the differentially expressed transcripts (https://MapMan.gabipd.org/). Common and unique DEGs with log2 fold changes that were obtained from DESeq2 output were used in MapMan.

For pathway analyses the KO ids were assigned to the significant DEGs using the blastKOALA (https://www.kegg.jp/blastkoala/) option at the Kyoto Encyclopedia of Genes and Genomes (KEGG) server. KEGG database integrates genomic information with functional information by collecting manually drawn pathway maps on cellular processes and gene annotations^42^. The assigned KO ids were used to perform pathway analysis using KEGG pathway tool.

#### Quantitative real-time polymerase chain reaction

Fourteen genes with various expression values were selected for validation of gene expression using quantitative RT-PCR (qRT-PCR). The selected genes and their primers are listed in Supplementary Table S1 with the corresponding Ensembl Gene IDs. The default parameters of a template protocol, SYBR Green I 96-II, of the Roche LightCycler 480 was used for the qRT-PCR.

## Results

### Screening of the treated lines

**Figure 1:**
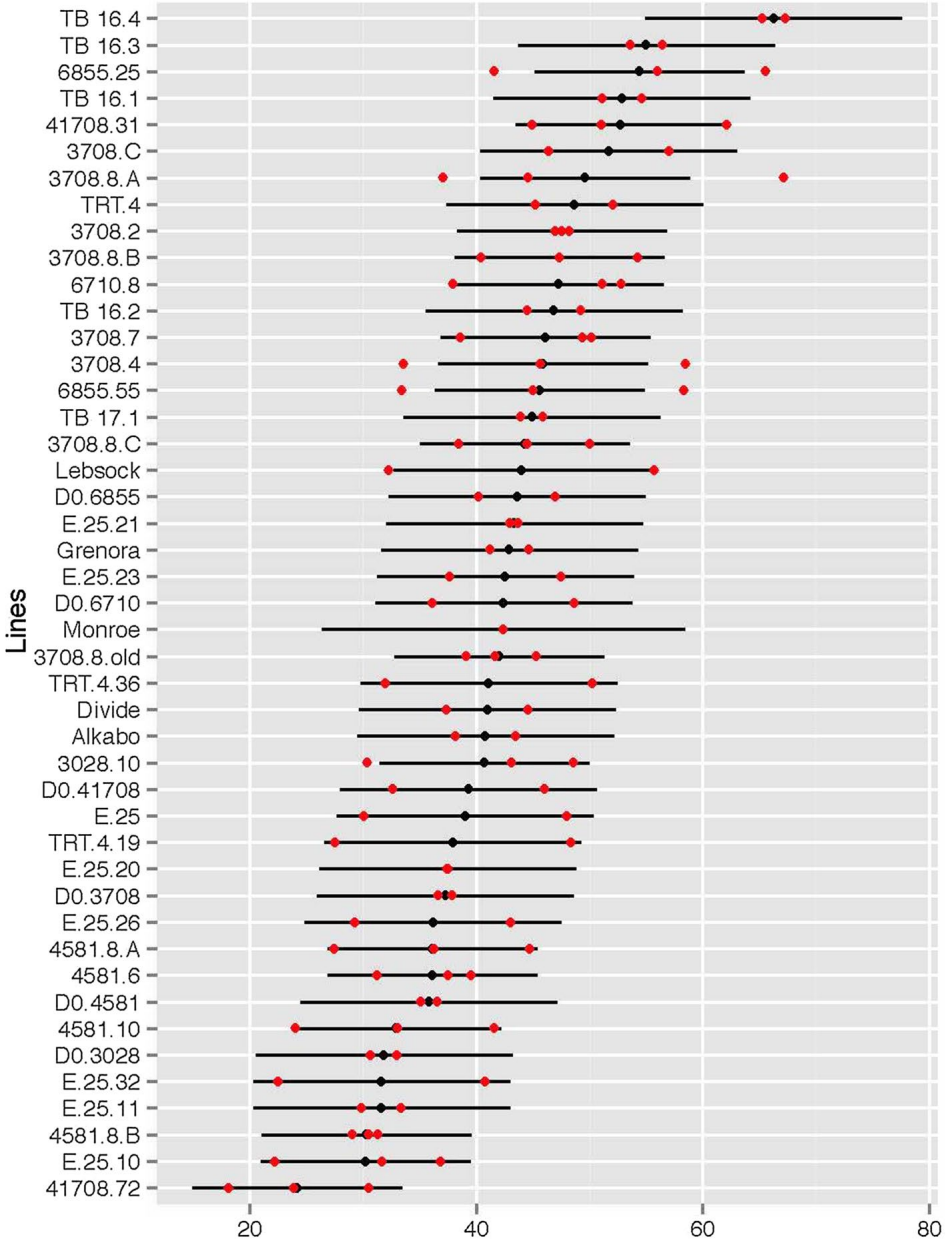
ANOVA plot showing the Fusarium head blight (FHB) disease severity estimates with 95% confidence intervals as black lines and dots. The lines at top and bottom have highest and lowest severity, respectively. Red dots indicate 80% trimmed mean of visual scoring data of disease severity, collected during the summer 2015 field season at Saint Paul, MN. Each red dot indicates one replicate. One replicate is an 80% trimmed mean of scores from 20 spikes. X-axis shows disease severity percentage and Y-axis show the name of the lines tested during the study.

**Figure 2:**
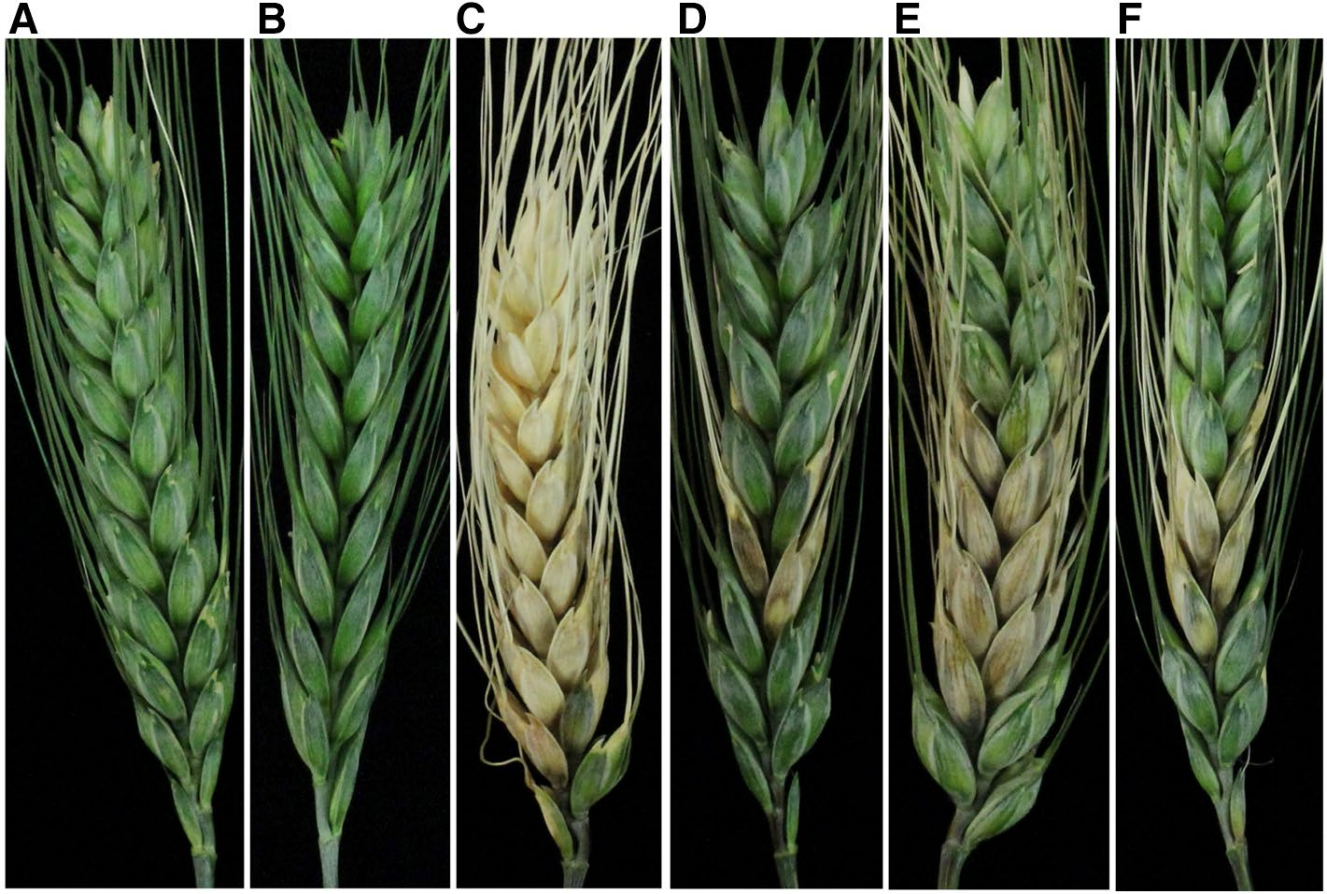
*Fusarium*-inoculated and control spikes of wheat showing various degree of disease severity. Uninoculated (**A**) and water inoculated (**B**) spikes as a control, susceptible check (**C**), resistant check (**D**), parental line (**E**) and M4 line showing disease severity upon point inoculations.

The germination rate following the 5-azacytidine treatments was approximately 50%, which was likely due to lethality of the treatment. A total of 415 treated seeds, of the 800 treated seeds, germinated, matured and grew into plants that produced seed. Of the 415, 32 lines were selected following elimination screening and were used in more rigorous testing. The scores for individual lines over different field experiments varied, but five, of the 32, lines showed promising result by having a lower FHB severity, FDK and DON values (Fig. 1, Supplementary Tables S2–S6). The data from the greenhouse inoculation experiments further supported the field data and showed a lower disease severity in the selected M4 lines (Table 1, Fig. 2). The selected M4 lines (E.25.10, E.25.11, E.25.23, E.25.32 and 41,708.72) had higher level of FHB resistance as compared with the parental lines and susceptible check (Figs. 1 and 2, Table 1, Supplementary Tables S2–S6).

**Table 1:**
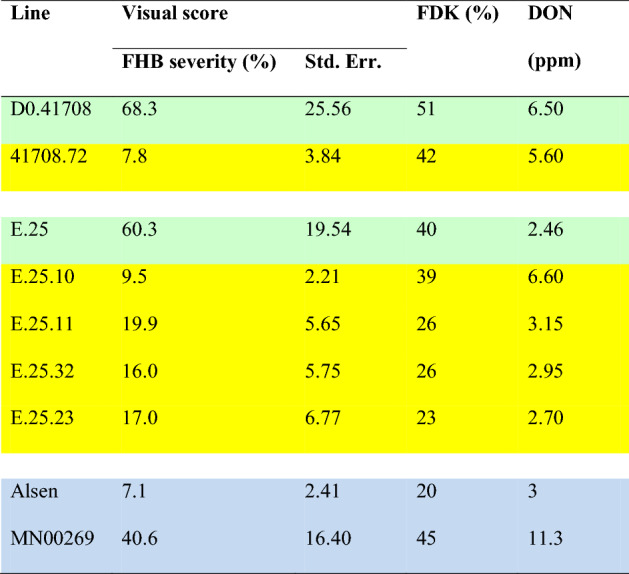
Visual sore of FHB disease severity, Fusarium damaged kernel (FDK) percentage and deoxynivalenol (DON) values from the greenhouse inoculations.

*Five best performing M4 lines are highlighted in yellow and the parents in light green.

Checks, Alsen (FHB resistant) and MN00269 (FHB susceptible), are highlighted in light blue.

The values are mean of three biological replicates.

For visual score, each biological replicates are mean of 15 spikes.

### Stability and inheritance of resistance

The BC_1_:F_3_ families, derived by crossing E.25.11 and 41,708.72 separately with Ben, were tested for inheritance and stability of the resistance. A total of 388 BC_1_:F_3_ families were tested in the summer 2017 field season together with the parental lines. Some of BC_1_:F_3_ families showed resistance similar to or better than the parental lines. Selected BC_1_:F_3_ families (50 best and 50 worst performing from the field data) were tested again in greenhouse, which supported the findings of the field study. The select BC_1_:F_3_ families performed on par with their parental lines. This preliminary data supports stable inheritance of resistance; however, additional testing is required to validate the number of resistant families to better estimate the number of genes underlying the trait.

### Estimation of genome wide DNA methylation level

**Figure 3:**
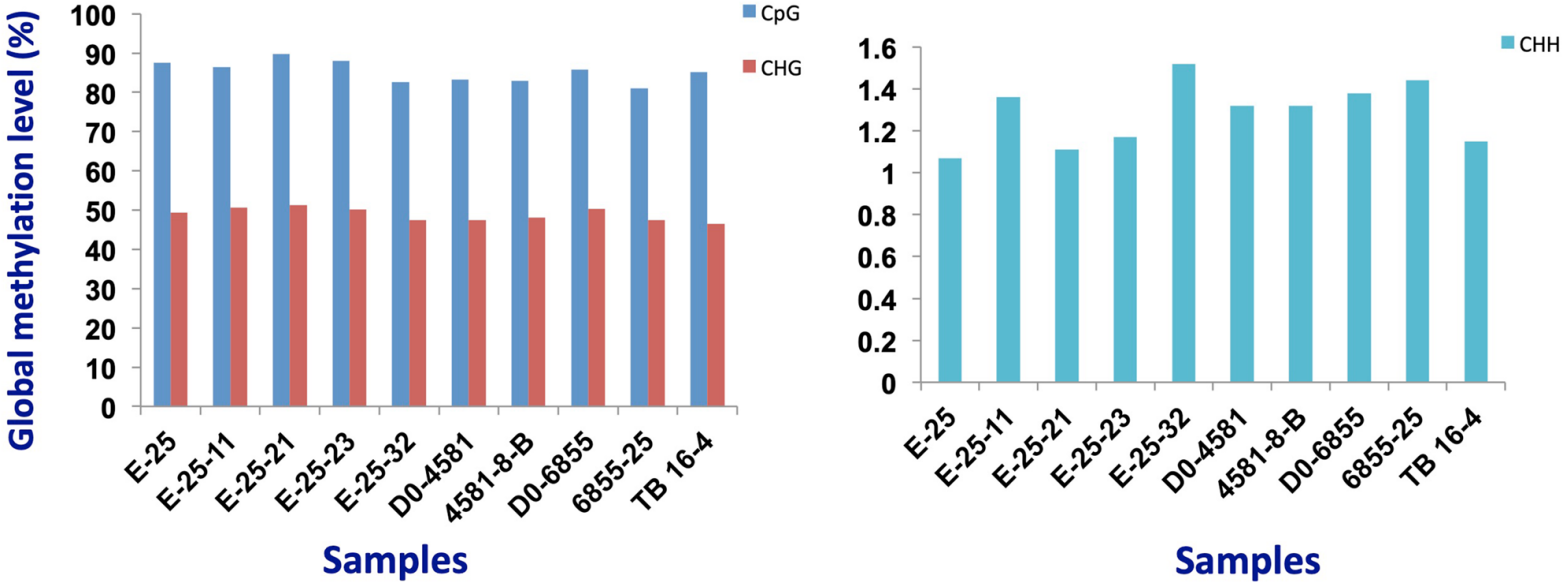
Global methylation level profiling using FASTmC method for M4 and parental lines. CpG islands are sites of transcription initiation. Methylation in the CpG site leads to transcriptional silencing of the genes. CHG: where H is A, C or T and CHH: where H is A, C or T.

Methylome analysis using FASTmC estimates genome-wide DNA methylation levels at all cytosine sequences^37^, which indicated no significant difference at the global level between parental and M4 lines. Some of the best performing M4 lines, E.25.11, E.25.23 and E.25.32 did not show a significant difference from their parental line, E.25 (Fig. 3). This suggests that M1 lines may have had significant level of demethylation that was unstable and which was lost during advancement, M1 to M4.

### Transcriptome analysis

On average, 40 million reads (range 31.51–49.62 million) were obtained for each sample, which were used to capture the dynamic transcriptome changes in resistant vs. susceptible line.

#### Differential expression of genes

**Figure 4:**
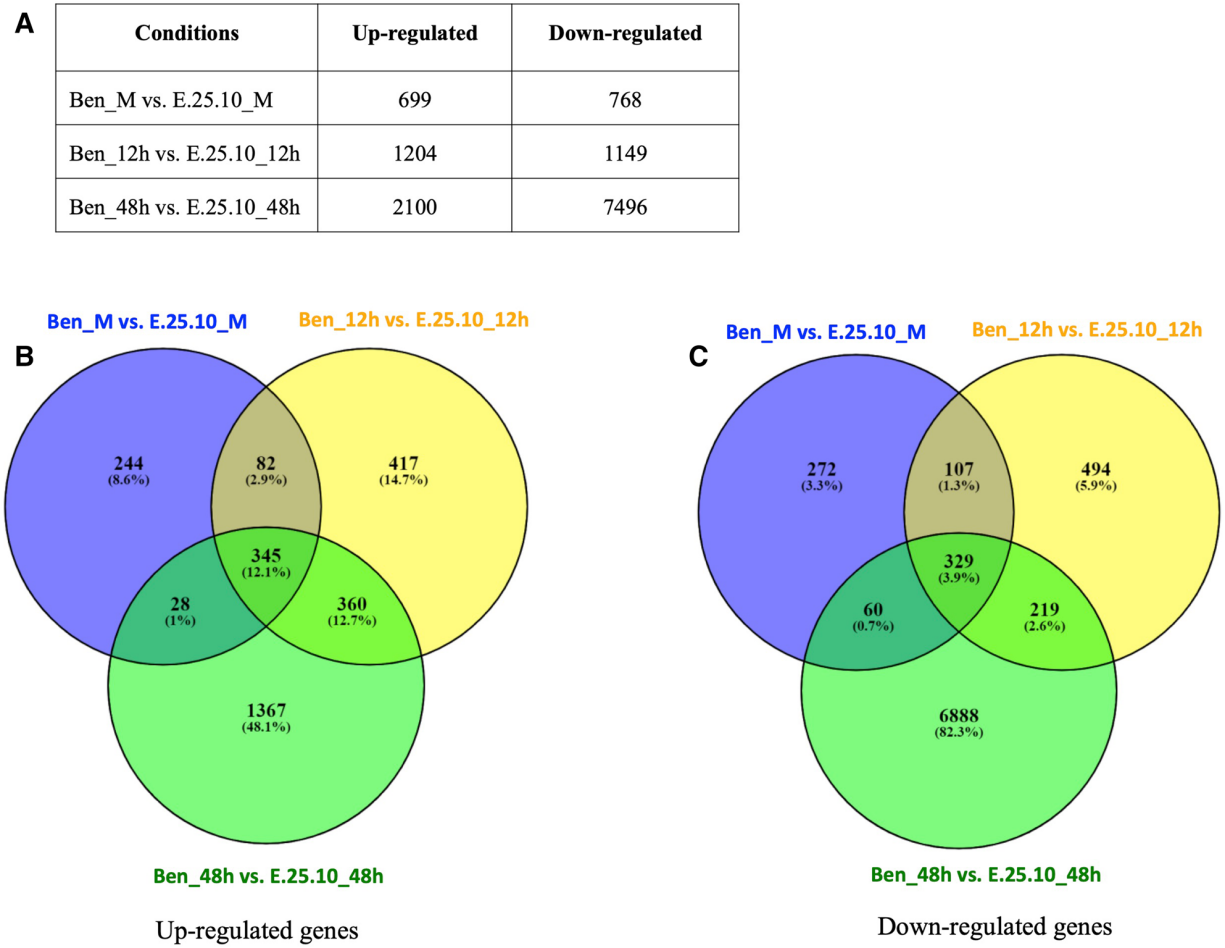
Table and Venn diagrams displaying differentially expressed genes in M4 line as compared to Ben. A total number of up- and down-regulated genes (log2 fold change ≥ 2 or ≤ -2 and a P < 0.01) in M4 line as compared to Ben in mock-inoculated at 48 h and *Fusarium* inoculated at 12 h and 48 h (**A**). Venn diagrams displaying up-regulated (**B**) and down-regulated (**C**) common (overlapping) and unique (non-overlapping) genes upon mock-inoculation at 48 h and *Fusarium* inoculation at 12 h and 48 h. Genes from panel (**A**) were used to display in Venn diagrams (**B**,**C**).

The total number of DEGs (log2 fold change ≥ 2 or ≤ -2 and a P < 0.01) at the two time points, 12 and 48 hpi, and mock treatment (M) are shown in Fig. 4A. A total of 699 and 768 genes were up- and down-regulated, respectively, in the Ben_M vs. E.25.10_M (mock-inoculated); 1204 and 1149 up- and down-regulated genes, respectively, in the Ben_12h vs. E.25.10_12h (12 hpi); and 2100 and 7496 up- and down-regulated genes, respectively, in the Ben_48h vs. E.25.10_48h (48 hpi) treatments (Fig. 4A). A total of 360 and 219 genes were commonly up- and down-regulated in the Ben_12h vs. E.25.10_12h and Ben_48h vs. E.25.10_48h (Fig. 4). A total of 417 and 1367 genes were up-regulated at 12 and 48 hpi, whereas, a total of 494 and 6888 genes were down-regulated at 12 and 48 hpi, respectively, and were unique to the provided conditions (Fig. 4). The genes, which were present commonly in mock-inoculated and the *Fusarium*-inoculated wheat at 12 and 48 hpi, were considered wound responsive, or plant growth and development related genes and were eliminated from further analysis (Fig. 4B,C). Genes that were common between the 12 hpi and 48 hpi samplings (360 and 219; Fig. 4B,C) were handled separately from the genes that were unique to either the 12 hpi (417 and 494; Fig. 4B,C) or 48 hpi sampling (1367 and 6888; Fig. 4B,C) and used in further analysis to examine early and late host responses.

Gene expression levels of fourteen genes, with a range of expression values, were validated using qRT-PCR. The relative fold change in the expression of selected genes was in agreement with the transcriptome results and validated its findings (Supplementary Fig. S2).

#### Functional assessment of differentially expressed genes

The agriGO categorization of differentially expressed common genes at the 12 and 48 hpi samplings revealed elevated expression of genes related to ion binding, cation binding, metal ion binding, defense response, cell wall organization and modification, hydrolase activity, enzyme regulator activity and other activities in the E.25.10 line, as compared to Ben (Supplementary Fig. S3, Supplementary Table S7), that could play essential roles in conferring FHB resistance. In addition, genes related to binding, metabolic processes and several other categories, which could also play roles in conferring FHB resistance, were down-regulated in the M4 line as compared to Ben (Supplementary Fig. S4; Supplementary Table S8). The genes, which were uniquely up- or down-regulated at 12 hpi or 48 hpi, were also categorized using agriGO. The genes, unique to the 12 hpi sampling time, were involved with cell wall synthesis, cell wall organization, oxidative stress, signal transmission, sequence specific DNA binding, and transcription factor activity (Supplementary Figs. S5 and S6, Supplementary Tables S9 and S10). The genes unique to the 48 hpi sampling time were involved in several other activities such as cell wall macromolecule catabolic process, protein ubiquitination, protein amino acid phosphorylation, regulation of gene expression, cell signaling and communication, multidrug transport, photosynthesis, negative regulation of catalytic activity and response to oxidative stress (Supplementary Figs. S7 and S8, Supplementary Tables S11 and S12).

**Figure 5:**
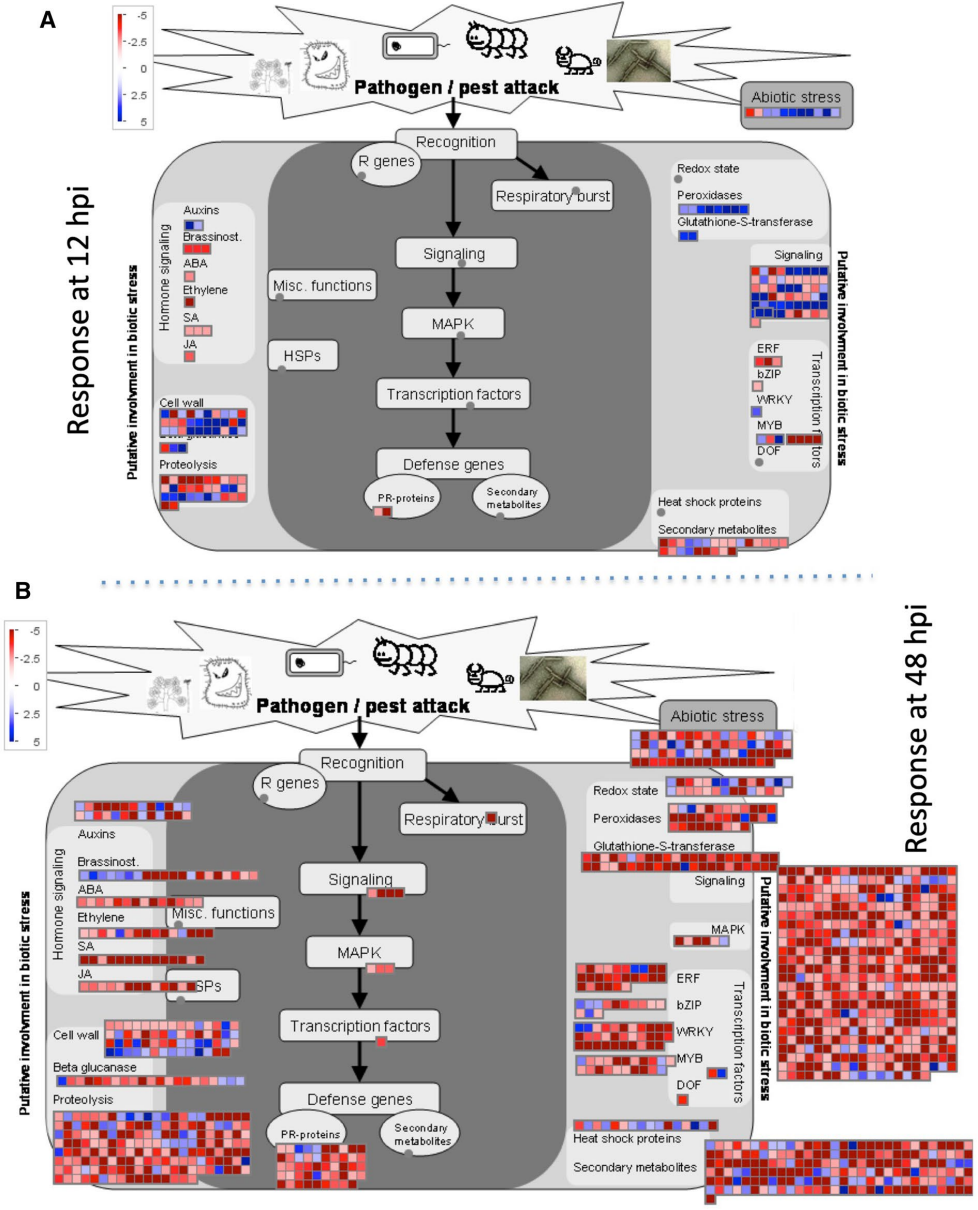
MapMan overview showing differentially expressed unique genes related to biotic and abiotic stress in M4 line at 12 hpi (**A**) and 48 hpi (**B**). Up-regulated genes are shown by light to deep blue color boxes and the down-regulated genes are shown by light to deep red color boxes. Color intensity show the level of expressions as indicated by the intensity bar on top left of each panel. Genes involved in same functions are clubbed together. Grey dots indicate that there were no significant expressions of such genes.

MapMan categorization revealed a significant level of DEGs, common to the 12 and 48 hpi sampling times, related to abiotic and biotic stresses, signaling, secondary metabolism, proteolysis, signaling, hormone signaling, and PR-proteins as differentially expressed in M4 line as compared to Ben (Supplementary Fig. S9). The transcriptional pattern of the same genes at 12 and 48 hpi differed significantly (Supplementary Fig. S9). MapMan categorization of the genes unique to the 12 or 48 hpi sampling times showed differentially expressed genes for abiotic stress, signaling, transcription factors, secondary metabolites, PR-proteins, proteolysis, cell wall, and hormone signaling (Fig. 5). As expected a large number of genes related to biotic stress were found differentially expressed at 48 hpi, as compared to 12 hpi, which coincides with fungal growth and accumulation from 12 to 48 hpi and suggests their role in the FHB resistance of the M4 line.

#### Pathways expressed or suppressed

**Figure 6:**
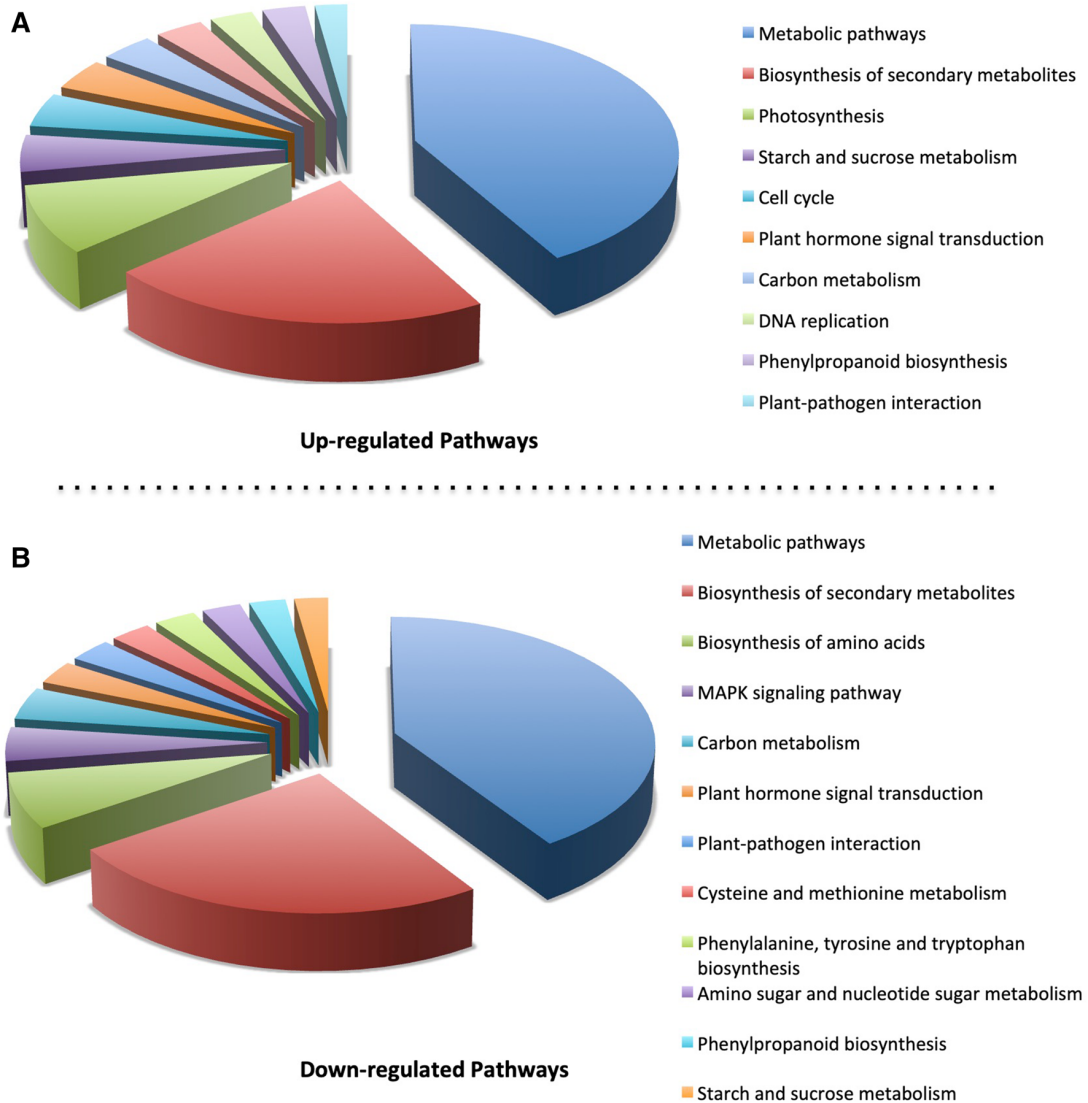
Classification of Kyoto Encyclopedia of Genes and Genomes (KEGG) pathways (42) detected in the present analysis. The differentially expressed genes showing up- and down-regulated pathways in M4 lines in response to *Fusarium* infection are shown here.

KEGG analysis demonstrated that diverse defense mechanisms were expressed more intensely or suppressed in the resistant M4 line at both 12 and 48 hpi as compared to the susceptible parental line (Fig. 6). A large number of genes representing metabolic pathways and biosynthesis of secondary metabolites were up- and down-regulated (Fig. 6). In addition, pathways involved in plant pathogen interaction, MAPK signaling, plant hormone signal transduction, starch and sucrose metabolism, photosynthesis and biosynthesis of secondary metabolites were significantly differentially expressed (Fig. 6).

## Discussion

In this study, we explored the possibility of generating a novel source of FHB resistance using DNA demethylation in durum wheat seeds that may be useful for future breeding efforts. Seeds of eight advanced durum lines were treated with 5-azacytidine, allowed to germinate, grow into plants, set seed and were propagated through to the M4 generation. This was done to assure that the epigenetic modification is stable and heritable. Five best performing lines were selected following a multi-year, multi-location and field vs. green house testing. Except a few variations, the performances of selected lines were consistent and the results were reproducible. The variation in FHB resistance in the M4 lines or in the parental lines observed during this study can be attributed to variability in FHB reactions, unfavorable environmental conditions for disease development or escape from inoculation which commonly occur in field FHB evaluation as documented by other researchers^43–45^. Stability and inheritance of resistance of M4 lines were further tested by crossing two M4 lines (E.25.11 and 41,708.72) with a susceptible parental line, Ben and developing BC_1_:F_3_ families and testing them by Fusarium inoculation, which confirmed that the modification is stable, genetic and heritable.

The M4 lines together with parental lines were subjected to global methylome level analysis to see if an overall difference in methylation level is evident. There were slight non-significant differences in methylation level in the M4 vs. parental lines. This suggests that unstable demethylation were lost in the process of advancement of M1 to M4 generation. Only the stable demethylations are present in M4 generation and contributing to resistance against FHB. The underlying mechanism was investigated by transcriptome analysis of a M4 line and a susceptible parent at two time points, 12 and 48 hpi, and including a mock inoculation. The transcriptome analyses showed that distinct groups of genes were activated at different stages (12 and 48 hpi) in the M4 line and the susceptible parent in response to Fusarium infection.

### Genes associated with *Fusarium* infection and plant response

To filter the genes associated with *Fusarium* infection, we eliminated the common genes that were shared between “Ben_M vs. E.25.10_M” and “Ben_12h vs. E.25.10_12h” and “Ben_M vs. E.25.10_M” and “Ben_48h vs. E.25.10_48h” (Fig. 4) and moved forward with the reminder. This was also done to remove the genes related to genetic background of Ben and E.25.10 lines. The macroconidia of *Fusarium* are reported to germinate within 6–12 h, and thus it was expected that the plants would elicit a defense response by 12 hpi. There were 360 up-regulated and 219 down-regulated genes common between 12 and 48 hpi. In addition, there were 417 up-regulated and 494 down-regulated unique genes at 12 hpi, which indicates an early response to infection. However, a more intense response was documented at 48 hpi as a total of 1367 up- and 6888 down-regulated unique genes were found. The common and unique genes represented several defense related pathways and were documented by Gene Ontology and MapMan analyses (Supplementary Figs. S3–S9 and Fig. 5). Numerous genes that were related to PR-proteins, hormone signaling, signaling, transcription factors, secondary metabolism, cell wall and oxidative stress were found differentially expressed in response to *Fusarium* infections.

#### Hormone signaling

Genes related to auxins, ABA, brassinosteroids, ethylene, jasmonic acid and salicylic acid were found differentially expressed at the 12 and 48 hpi samplings. However, there were significant differences in the number of genes between the two sampling times. Elevated levels of auxin in plant tissues have been observed previously following pathogen infection^46^. In the current analysis, at least 26 genes associated with auxin related pathways were found differentially expressed. Brassinosteroids are plant-specific steroidal hormones, which are involved in signal transduction that results in the regulation of expression of several hundred genes including stress related genes^47^. Twenty genes related to brassinosteroids were found differentially expressed in the current study, which is significant considering that these are known to play a role in signal transduction. Studies of the co-application of exogenous ABA or jasmonic acid with *F. graminearum* demonstrated increased wheat susceptibility, suggesting a major negative role of ABA and jasmonic acid in FHB resistance^48,49^. As all 14 genes related to ABA and 13 genes related to jasmonic acid were found down regulated in the current analysis, our data would support the role of ABA and jasmonic acid in increased wheat susceptibility. Ethylene is induced in plant tissues upon pathogen challenge; however, the role of ET in plant defense is ambiguous due to both positive and negative effects observed during host–pathogen interactions^50^. The reported studies match our findings where 13 out of the 15 ethylene related genes were down-regulated in the resistant line. SA pathway has been reported to play critical roles in resistance against *F. graminearum* and genes related to the SA pathway have been reported to be up-regulated in previous studies^51,52^. However, all of the 14 genes associated with SA pathway in the current analysis were found down regulated. This may indicate a genotype-dependent response or the importance of the timing of activation of the SA pathway, as has been reported in wheat by others^53,54^.

#### Pathogenesis related proteins

Induction of pathogenesis related (PR) proteins, PR-1, PR-2, PR-3, PR-4, and PR-5, have been documented in *Fusarium* infected wheat 6–12 hpi, reaching the highest levels at 36–48 h^55^, as was in our study (Fig. 5). Forty nine PR proteins were found differentially expressed during the current analysis and were categorized as; NB-ARC domain-containing disease resistance proteins, receptor like proteins 46 (RLP46), kinase family proteins with leucine-rich repeat (LRR) domains, receptor like proteins 27 (RLP27), leucine-rich repeat (LRR) family proteins, leucine-rich repeat serine/threonine protein kinases such as FLS2, receptor like proteins 33 (RLP33), acidic endochitinase precursors, LRR and NB-ARC domains-containing disease resistance proteins, PR-protein 1 precursors, PR-protein PRMS precursors, proteins Z (Z4), or serine protease inhibitor (SERPIN) family proteins. Two LRR receptor like kinases (LRR-RLK), one from barley (*HvLRRK-6H*) and one from wheat (*TaLRRK-6D*), were found highly induced upon *F. graminearum* infection. Virus-induced gene silencing (VIGS) of these genes resulted in susceptibility of the host plants^56^.

#### Transcription factors

In response to external stimuli, transcription factors generate primary responses by up- or down-regulating downstream gens as a part of plant defense mechanism^57^. Thirteen bZIP transcription factor family proteins were found differentially expressed (4 up- and 9 down-regulated) in the current analysis. The present study also documented differential expression of at least 26 genes encoding for AP2/EREBP (ERF) transcription factor family proteins. The WRKY transcription factor family had 32 genes differentially expressed during the present analysis. The types of WRKY proteins were WRKY35, WRKY11, WRKY65, WRKY51, WRKY50, WRKY33, WRKY30, WRKY41, WRKY71, WRKY55, WRKY3, WRKY11, WRKY40, WRKY46 and WRKY9. *WRKY*, *Myb* and *bZIP* transcription factors have been shown to paly important roles in FHB resistance in multiple studies^58,59^, which suggests why they were differentially expressed in the current analysis. A bZIP transcription factor, *Fgap1*, has been reported to mediate oxidative stress response in *Fusarium* infection^60^. *TaWRKY45* expression was enhanced in response to *Fusarium* infection and a transgenic wheat plant constitutively expressing *TaWRKY45* showed increased resistance to FHB^61^. Another WRKY protein, *TaWRKY70*, was found to have higher expression and regulate downstream genes in response to *Fusarium* infection. VIGS silencing of the *TaWRKY70* resulted in a higher fungal biomass and susceptibility^62^.

#### Signaling

Four hundred and seventy seven genes related to signaling pathways were found differentially expressed in response to *Fusarium* infection in the M4 line in this study (Fig. 5). Of the 477 genes identified, 123 genes belonged to the LRR receptor kinases family, 55 to S-locus glycoprotein-like receptor kinases, 41 to calcium signaling, 39 to legume-lectin receptor kinases, 37 to DUF 26 receptor kinases, 37 to unclassified receptor kinases, 35 to cell wall associated receptor kinases, 17 to G-proteins, 15 to *Catharanthus roseus*-like RLK1 receptor kinases, 14 to thaumatin-like receptor kinases, 14 to sugar and nutrient physiology, 7 to light reactions and 6 to MAP kinases. LRR receptor kinases have been extensively studied because of their role in host response and have been discussed previously (See “Pathogenesis related proteins” on PR-proteins). S-locus glycoprotein-like receptor kinases has been reported to play a negative role in plant defense responses^63^, which supports the finding that 54, of the 55 S-locus glycoprotein like receptor kinases genes identified in the current analysis, were down-regulated. Ca2 + signaling is crucial for plant innate immunity as it mediates signaling process by variation in cytosolic Ca2 + concentration^64^. Differential expression of 41 Ca2 + signaling genes found in this study is in accordance with their previously reported role in plant defense. Overexpression of a L-type lectin-like protein kinase 1 (*AtLPK1*) was demonstrated to confer resistance against *Botrytis cinerea* in *Arabidopsis thaliana* by inducing stronger expression of a group of defense-related genes^65^. Significant differential expression of 39 legume-lectin genes in the present study further supports the role of legume-lectin genes in resistance to fungal pathogens in durum. G-protein coupled receptors (GPCRs) transmit signals, from extracellular challenges to intracellular G-proteins, that ultimately direct the appropriate biological response in host cells^66^. Significant differential expression of 17 G-proteins in the present study is well aligned with its reported role in biotic and abiotic stresses.

#### Cell wall

Fifty-nine proteins with a role in cell wall synthesis, organization or degradation were found to be differentially expressed in the current analysis. In addition to the 59 proteins with a role in cell wall synthesis, organization or degradation, we observed the differential expression of 21 glucanases and 4 chitinase genes in our dataset, which is in line with the previous findings^57,67^.

Of the 59 genes, 8 genes were associated with cell wall modifications, 6 were cellulose synthase genes, 5 were glucuronoxylan genes, 16 were cell wall degradation related genes (cellulases and beta-1,4-glucanases, mannan-xylose-arabinose-fucose and pectate lyases and polygalacturonases), 10 genes were related to cell wall precursor synthesis, 9 genes were related to cell wall hemicellulose synthesis and 5 genes represented cell wall pectin esterases. Cell wall composition and lignification has been reported to play important roles in conferring host resistance to FHB^67^. Cell wall features transferred from common wheat to durum wheat, by generating recombinant inbred lines (RILs) obtained by crossing the hexaploid resistant wheat with the susceptible durum wheat, were reported to improve FHB resistance significantly^68^.

#### Secondary metabolites

Plant deal with *Fusarium* infection and mycotoxin accumulation through the inhibition of toxin biosynthesis or converting the mycotoxin into less toxic compounds. The inhibition of toxin biosynthesis is handled through antioxidant properties of secondary metabolites. The main secondary metabolites with antioxidant activity belong to phenolic compounds, carotenoids and tocopherols in cereals^69^. A total of 181 genes representing for secondary metabolites biosynthesis were differentially expressed in the M4 line as compared to the susceptible line. Of the 181 genes, 45 genes were involved in lignin biosynthesis and belonged to phenylpropanoids, 23 were anthocyanins, 7 were chalcones, 21 were dihydroflavonols, 16 were flavonols and all of them belonged to flavonoids, 20 belonged to isoprenoids and were involved in mevalonate pathway, carotenoids pathway or terpenoids pathway and 7 were simple phenols. Phenolics are the major contributors to total antioxidant capacity of cereals and are divided into two groups: flavonoid phenylpropanoids and non-flavonoid phenylpropanoids which includes anthocyanins, chalcones, flavones, flavonols, flavanones, flavanols, stilbenes, lignans, and phenolic acids^70^. We documented differentially expressed gens, representing lignins, chalcones, flavonols, and phenols, which is in agreement to the previous reports by providing FHB resistance to M4 plants.

#### Oxidative stress

An FHB-resistant wheat variety has been reported to show rapid induction of ascorbate peroxidase (APX) and polyphenol oxidase (PPO) activity, correlated with the activity of antioxidative enzymes^71^. In the current study, 27 genes related to redox (thioredoxin, glutaredoxins, ascorbate and glutathione), 33 to peroxidases and 44 to glutathione S transferases were recorded as being differentially expressed. The significant differential expression of glutathione S transferases in the M4 line is in accordance with several previous studies, which have identified a role of glutathione S transferases in FHB resistance, with the resistance conferred by detoxifying the DON and thus reducing the aggressiveness of the pathogen^72,73^.

#### Abiotic stress

Plants are exposed to a wide range of biotic and abiotic stresses at the same time, or consecutively. Studies have also reported stress specific or independent commonalities in the response to stresses in different plant systems^74,75^. The crosstalk between the biotic and abiotic stress signaling pathways becomes synergistic and may lead to a cross-tolerance and enhancement of the plant’s resistance to pathogens^76^. The significant differential expression of 70 abiotic stress (heat, cold, drought and salinity) related genes in response to *Fusarium* infection in the current analysis also supports the commonalities between abiotic and biotic stress response as reported by other researchers. Based on the previous reports and findings of this study, some or all 70 abiotic stress related genes may also have contributed to the FHB resistance in the M4 line included in this study.

### Altered pathways and mechanism of resistance in M4 line

In this study we documented at least 10 pathways that had 10 or more genes up-regulated and 23 pathways that had 10 or more genes down-regulated in response to *Fusarium* infection. Metabolic pathways and the pathways involved in the biosynthesis of secondary metabolites recorded the highest number of genes, which were up- or down-regulated (Fig. 6). The high number of DEGs in metabolic and secondary metabolite pathways suggests that the *Fusarium*-infected M4 plants need both energy and antioxidants in order to fight the *Fusarium* infections as reported by Gorinstein et al.^70^ and Erayman et al.^77^. A large number, 29, of genes from the MAPK signaling pathway were found to be differentially expressed in this study and these genes might have helped the plant in a late defense response to pathogen, or through stomatal development or maintaining the homeostasis of reactive oxygen species (Supplementary Fig. S14). A significant number of up-regulated genes were also found in association with photosynthesis and starch and sucrose metabolism pathways (Supplementary Figs. S10 and S11). Fifteen genes were documented to be up-regulated in photosynthesis pathways including photosystem I and II and the photosynthetic electron transport system (Supplementary Fig. S10). Twenty-six DEGs were recorded as being up-regulated in starch and sucrose metabolism pathway (Supplementary Fig. S11). Starch and sucrose metabolism pathway is reported to play critical role in FHB resistance in previous studies^77,78^. This suggests that the M4 plants in this study were capable of generating more energy, as compared to susceptible plants, and therefore could respond better to *Fusarium* infection. Genes related to plant pathogen interactions and plant hormone signal transduction pathways were found significantly differentially expressed (Fig. 6). Eighteen genes, representing the plant-pathogen interaction pathway, had significant differential expression in response to *Fusarium* infection, and may contribute to resistance in the M4 line via hypersensitive response, cell wall reinforcement, induction of defense related genes and stomatal closure (Supplementary Fig. S12). In addition, twenty-four genes from plant hormone signal transduction pathways were also recorded as being differentially expressed in this study. These genes regulate cell enlargement, cell division, shoot initiation, stomatal closure and senescence (Supplementary Fig. S13).

## Conclusion

This study explored an alternative method for generating FHB resistance in durum wheat, as most of the durum wheat cultivars are susceptible to FHB. Eight of the advanced durum lines were treated with 5-azacytidine to remove methylation and allow the expression of probable candidate genes. Treated lines were advanced four generations (M4) and tested for the FHB resistance over multiple years and locations. Five of the treated lines showed promising resistance to FHB and were selected for methylome level analysis and transcriptome analysis. Methylome level/percentage analysis did not show a significant difference; however, transcriptome analysis indicated significant differences between the parental and M4 line. Genes that were differentially expressed more than two fold in the M4 line were filtered by eliminating the genes expressed in the mock-inoculated control plants. Differential gene expression patterns associated with the M4 line indicated multi-facetted defense responses.

We performed transcriptome profiling of wheat spikes against the *Fusarium* infection at 12 and 48 hpi to explore the early and late response. The M4 line activated defense systems by differentially expressing transcripts related to PR proteins, transcription factors, signaling, secondary metabolites, proteolysis, cell wall, oxidative stress and hormone signaling. The transcripts related to binding activity were the processes most affected by *Fusarium* infection in the M4 lines. In addition, signaling, metabolic processes, PR-proteins and oxidative stress associated transcripts were higher in the M4 lines. The KEGG pathway enrichment indicated that genes involved in metabolic pathways and the biosynthesis of secondary metabolites were highly affected. Our results demonstrate how a new approach to generating resistance to a notorious pathogen, *Fusarium*, can help with developing new durum wheat varieties with improved FHB resistance. The findings of the study may assist in breeders in the development of new varieties with improved FHB resistance by utilizing M4 lines as a parent. In future work we plan to undertake deep RNA sequencing of multiple M4 and parental lines to locate individual genes or combinations of genes conferring FHB resistance in durum wheat.

## Supplementary Information

Supplementary Information.
