# Supplementary material for: Epigenetic regulation of gene expression improves Fusarium head blight resistance in durum wheat
Source: Sci Rep. 2020 Oct 19;10:17610. doi: 10.1038/s41598-020-73521-2 (PMC7572394; doi:10.1038/s41598-020-73521-2)
Supplement: Supplementary file 1 — Supplementary Information. [file 41598_2020_73521_MOESM1_ESM.docx]

**Epigenetic regulation of gene expression improves fusarium head blight resistance in durum wheat**

**Jitendra Kumar^1^, Krishan M. Rai^2^, Seyedmostafa Pirseyedi^3^, Elias M. Elias^3^, Steven Xu^4^, Ruth Dill-Macky^1^ and Shahryar F. Kianian^5*^**

^1^Department of Plant Pathology, University of Minnesota, St. Paul, MN, USA

^2^Department of Microbial and Plant Genetics, University of Minnesota, St. Paul, MN, USA

^3^Department of Plant Sciences, North Dakota State University, Fargo, ND, USA

^4^USDA-ARS Cereal Crops Research Unit, Edward T. Schafer Agricultural Research Center, Fargo, ND, USA

^5^USDA-ARS Cereal Disease Laboratory, St. Paul, MN, USA

**Supplementary Figure 1.** Flowchart representing process of 5-azacytidine treatment and selection of the resistant lines.


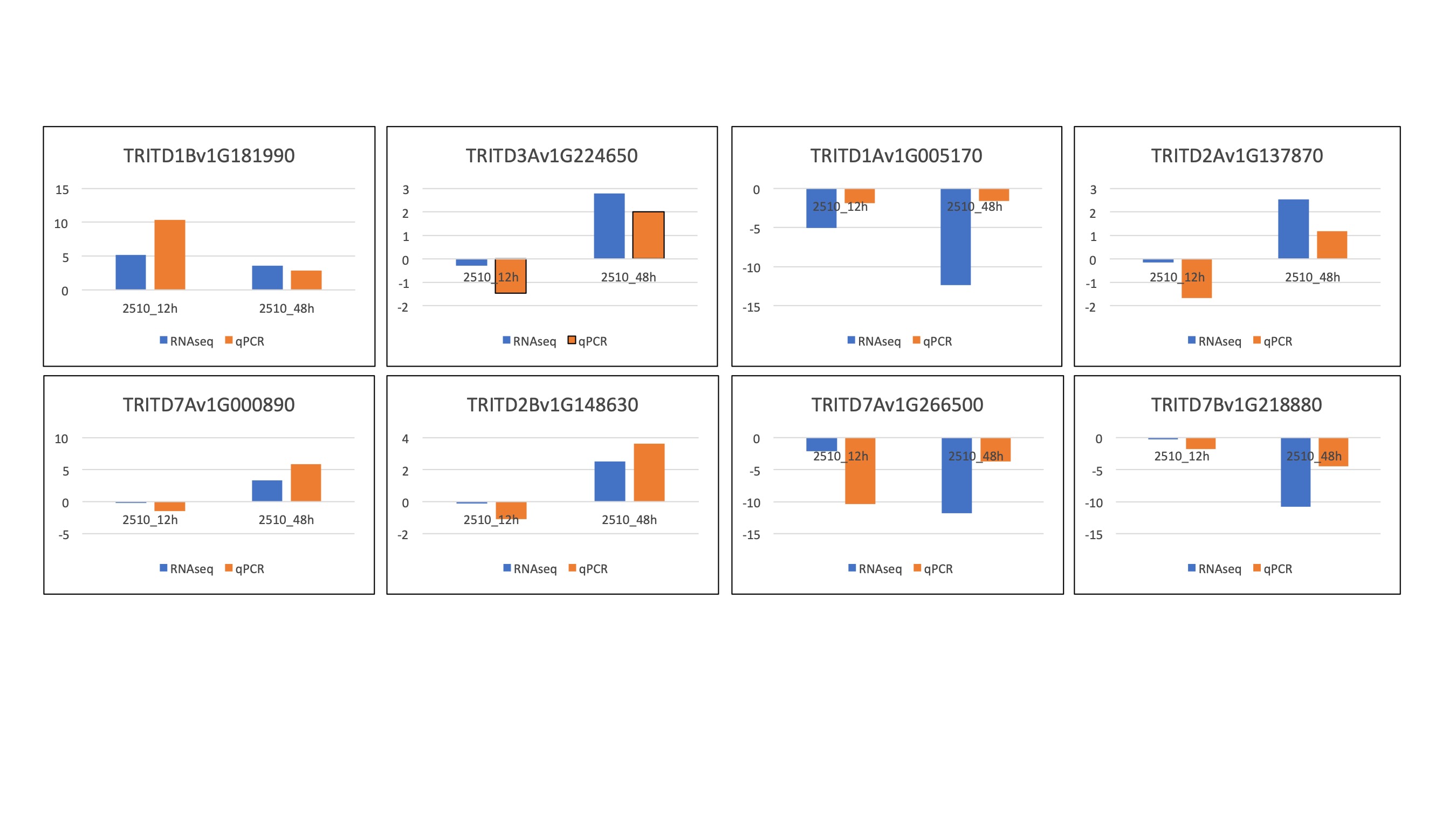


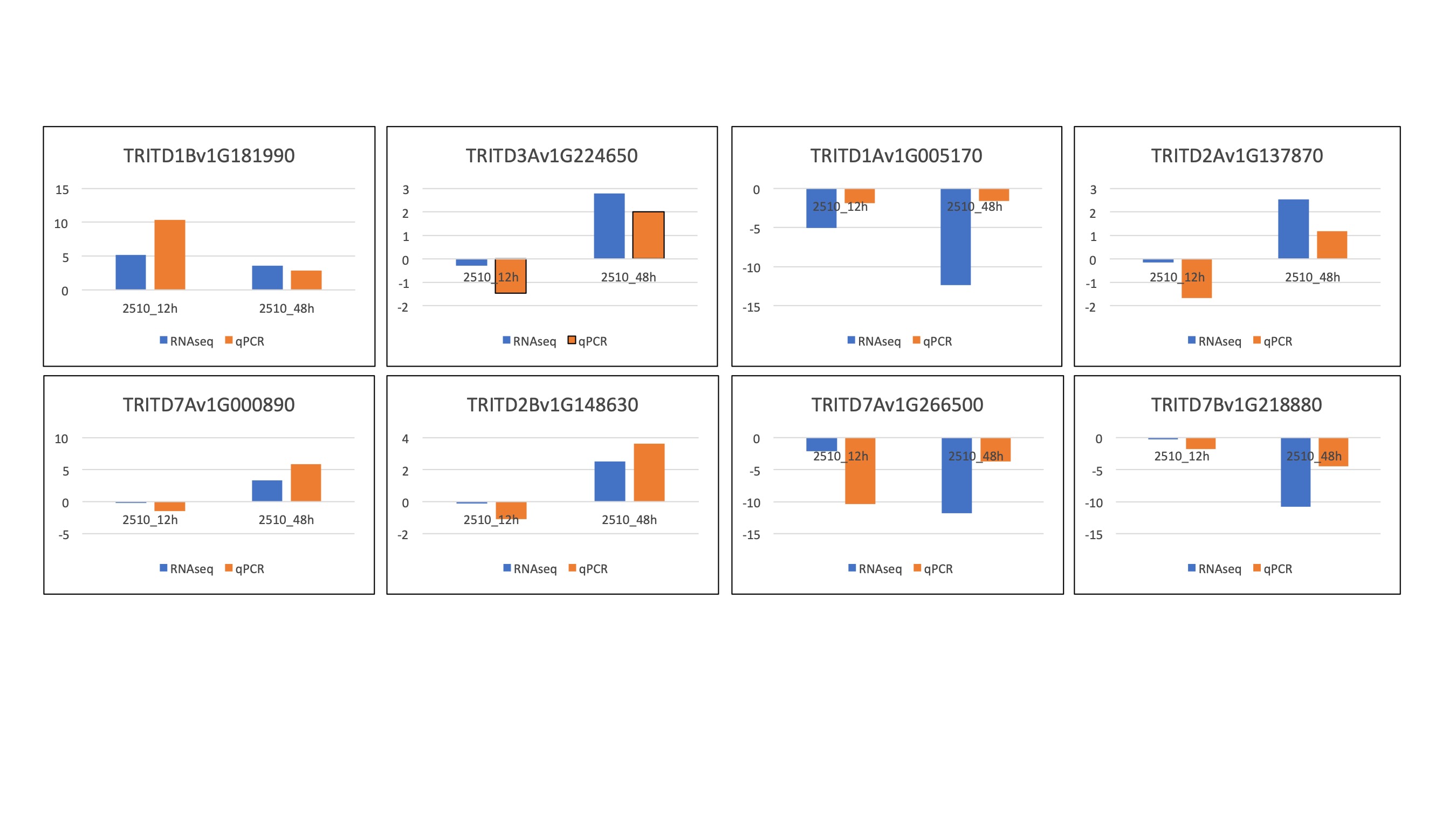


**Supplementary Figure 2.** Quantitative real-time PCR based validation of gene expression in M4 line at 12 hour (12h) and 48 hour (48h) post infection as compared to Ben at 12h and 48h. The expression values estimated by RNA-Seq and real-time are in blue and orange, respectively.


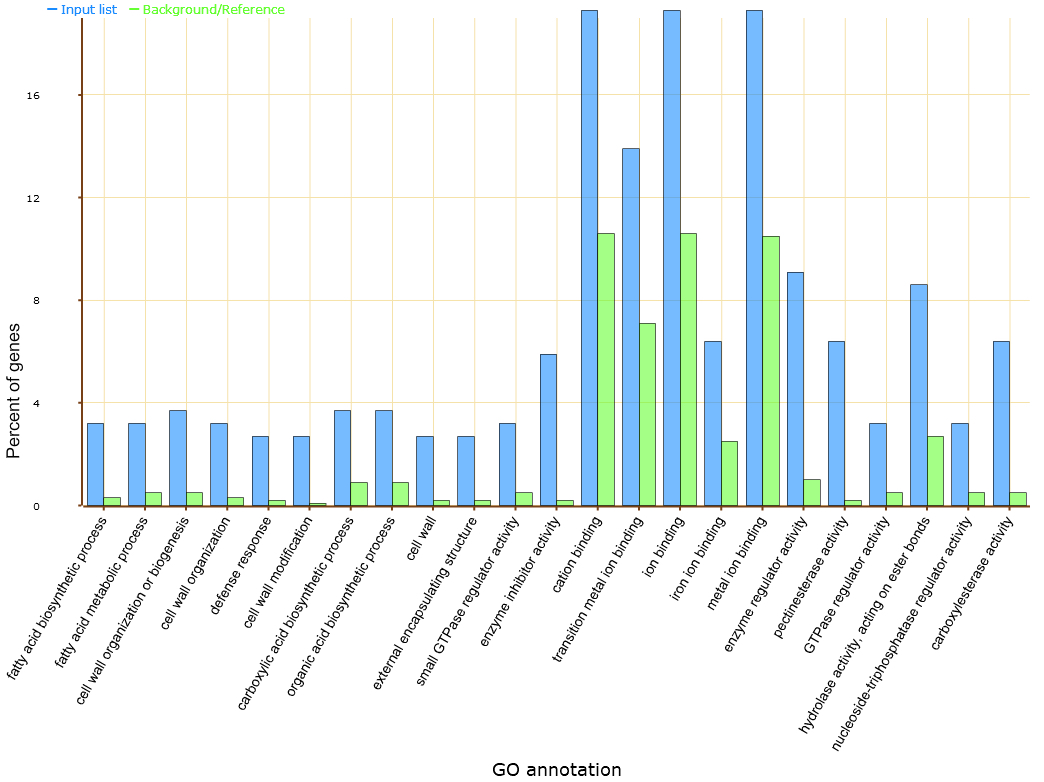


**Supplementary Figure 3.** Bar graph representation of enrichment of gene ontology (GO) term for genes commonly up-regulated at 12 hpi and 48 hpi in M4 line as compared to Ben. X-axis represents GO annotation for predicted functions of the genes and the Y-axis shows percent of genes for each functions. Blue bars represent percent of genes in input list and the green bars represent genes in reference list.


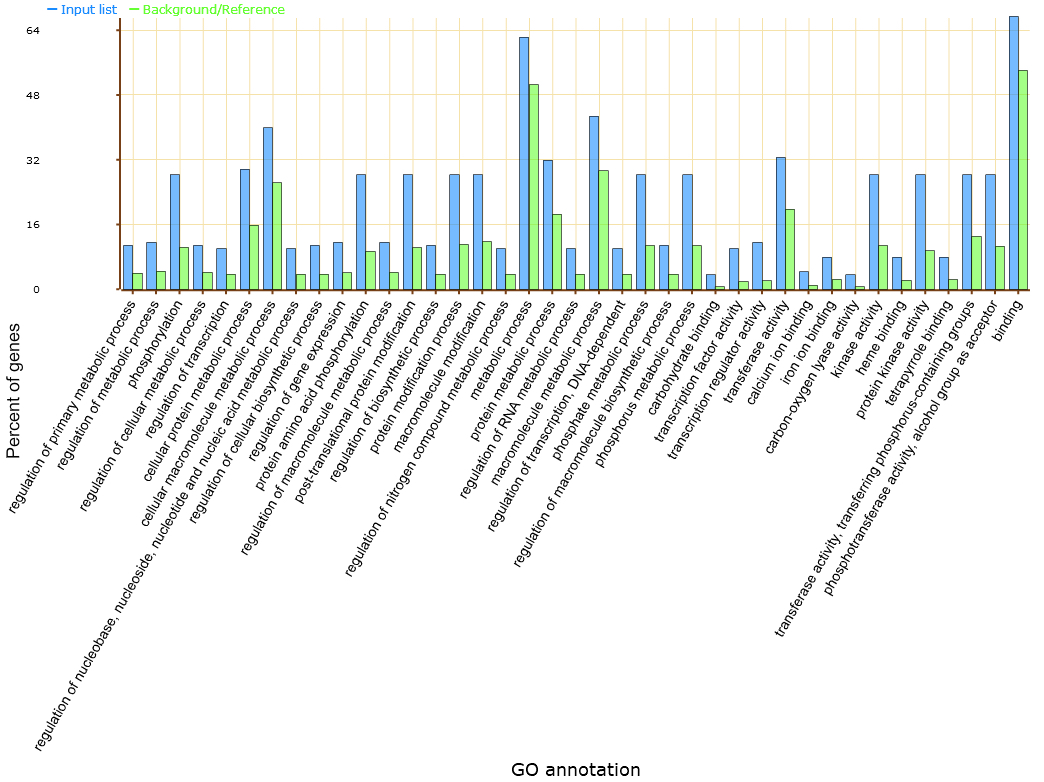


**Supplementary Figure 4.** Bar graph representation of enrichment of gene ontology (GO) term for genes down-regulated commonly at 12 hpi and 48 hpi in M4 line as compared to the susceptible line. X-axis represents GO annotation for predicted functions of the genes and the Y-axis shows percent of genes for each functions. Blue bars represent percent of genes in input list and the green bars represent genes in reference list.


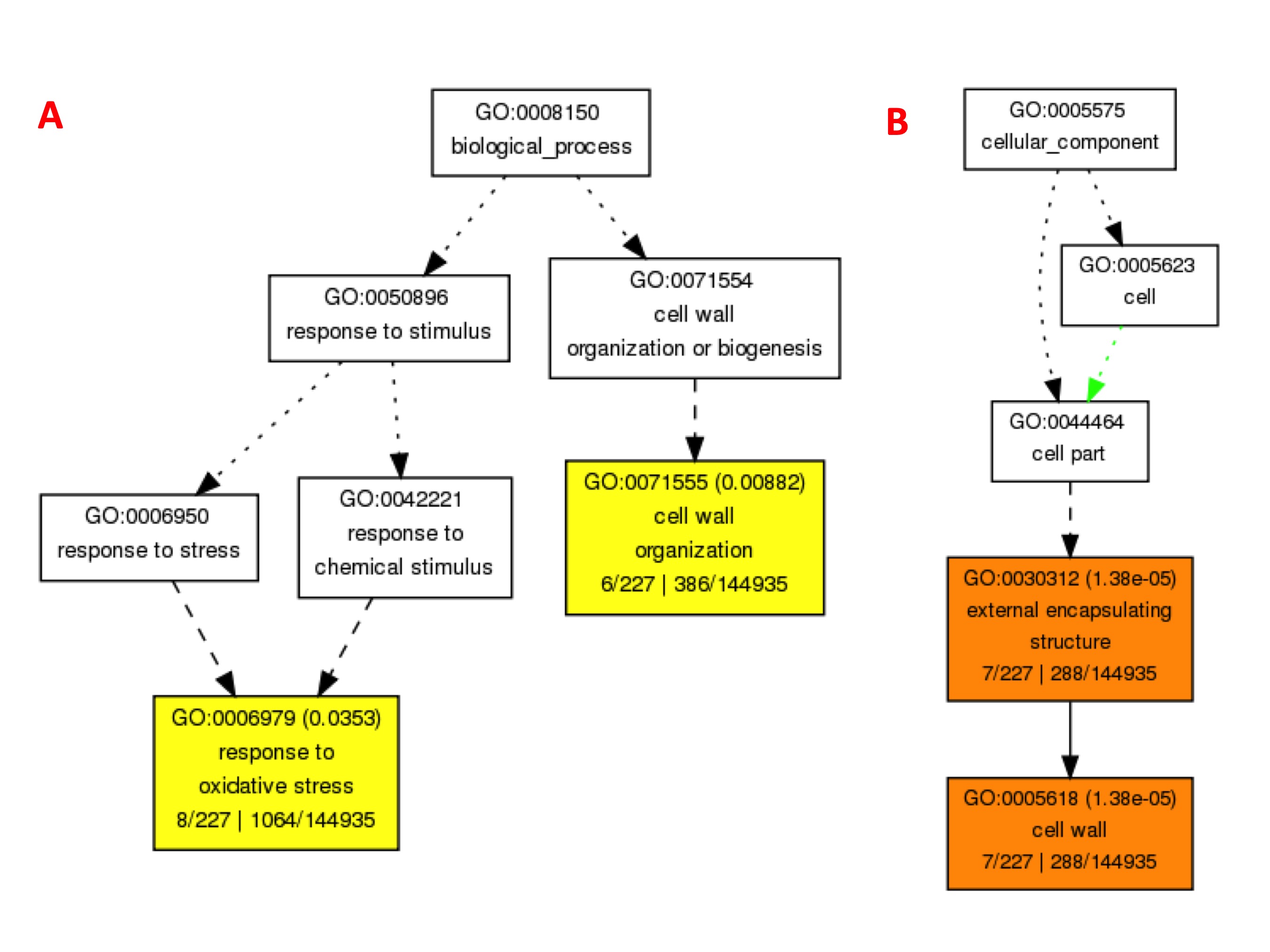


**Supplementary Figure 5.** Distribution of up-regulated genes, unique to 12 hpi in M4 line, represented with their corresponding gene ontology (GO) Accession numbers. The distribution was done on the basis of biological process (A) and cellular component (B). The biological process at 12 hpi emphasize on the oxidative stress and cell wall organization (A), whereas the cellular components diagram show significance of cell wall organization at 12 hpi (B). Significant genes and functions are shown in colored boxes. The colored boxes also show annotated/total number in query list and, annotated/total number in background.


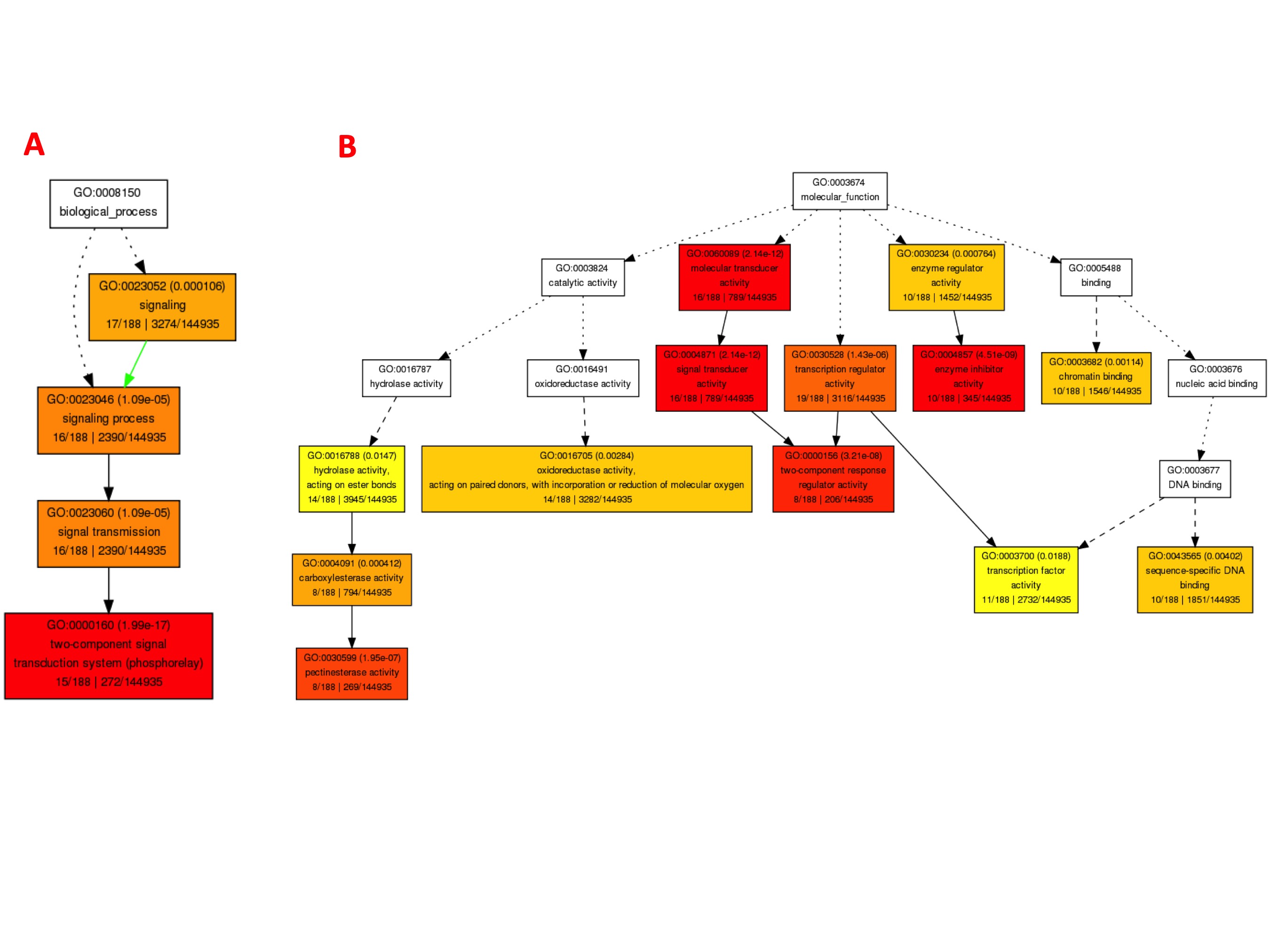


**Supplementary Figure 6.** Distribution of down-regulated transcripts represented with their corresponding GO Accession numbers unique to 12 hpi in M4 line. The distribution was done on the basis of biological process (A) and molecular function (B). The biological process at 12 hpi emphasize on the signal transmission (A), whereas the molecular function diagram show significance of DNA binding, transcription factors, enzyme and enzyme inhibition activity at 12 hpi (B). Significant genes and functions are shown in colored boxes. The colored boxes also show annotated/total number in query list and, annotated/total number in background.


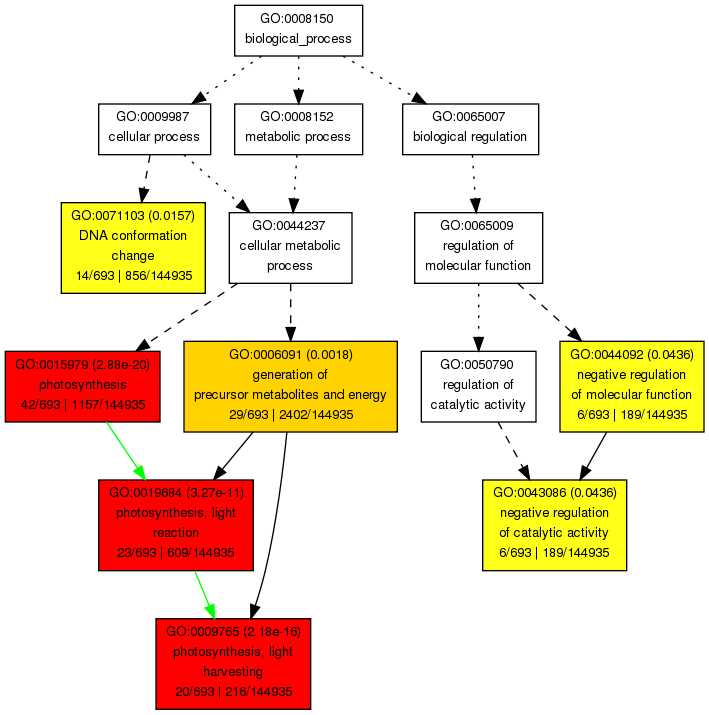


**Supplementary Figure 7.** Distribution of up-regulated transcripts represented with their corresponding GO Accession numbers unique to 48 hpi in M4 line. The distribution was done on the basis of biological process. The biological process at 48 hpi emphasize on the generation of the precursor metabolites and energy, photosynthesis and negative regulation of molecular functions. Significant genes and functions are shown in colored boxes. The colored boxes also show annotated/total number in query list and, annotated/total number in background.


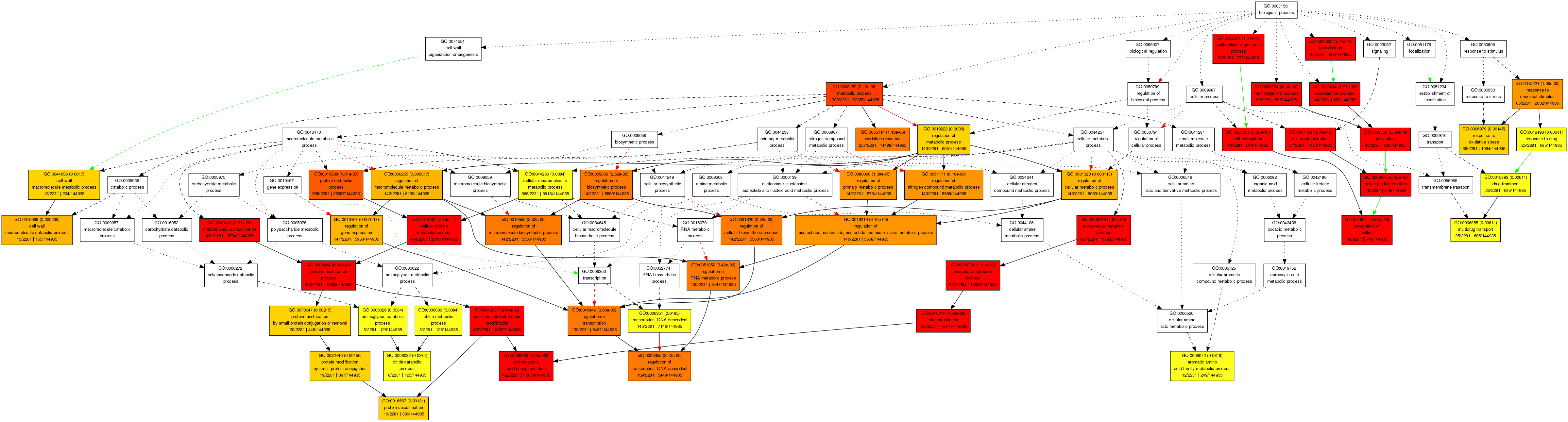


**Supplementary Figure 8.** Distribution of down-regulated transcripts represented with their corresponding GO Accession numbers unique to 48 hpi in M4 line. The distribution was done on the basis of biological process. The biological process at 48 hpi emphasize on cell wall, protein ubiquitination, regulation of transcription, response to chemical stimulus, phosphorylation and regulation of biosynthetic process. Significant genes and functions are shown in colored boxes. The colored boxes also show annotated/total number in query list and, annotated/total number in background.


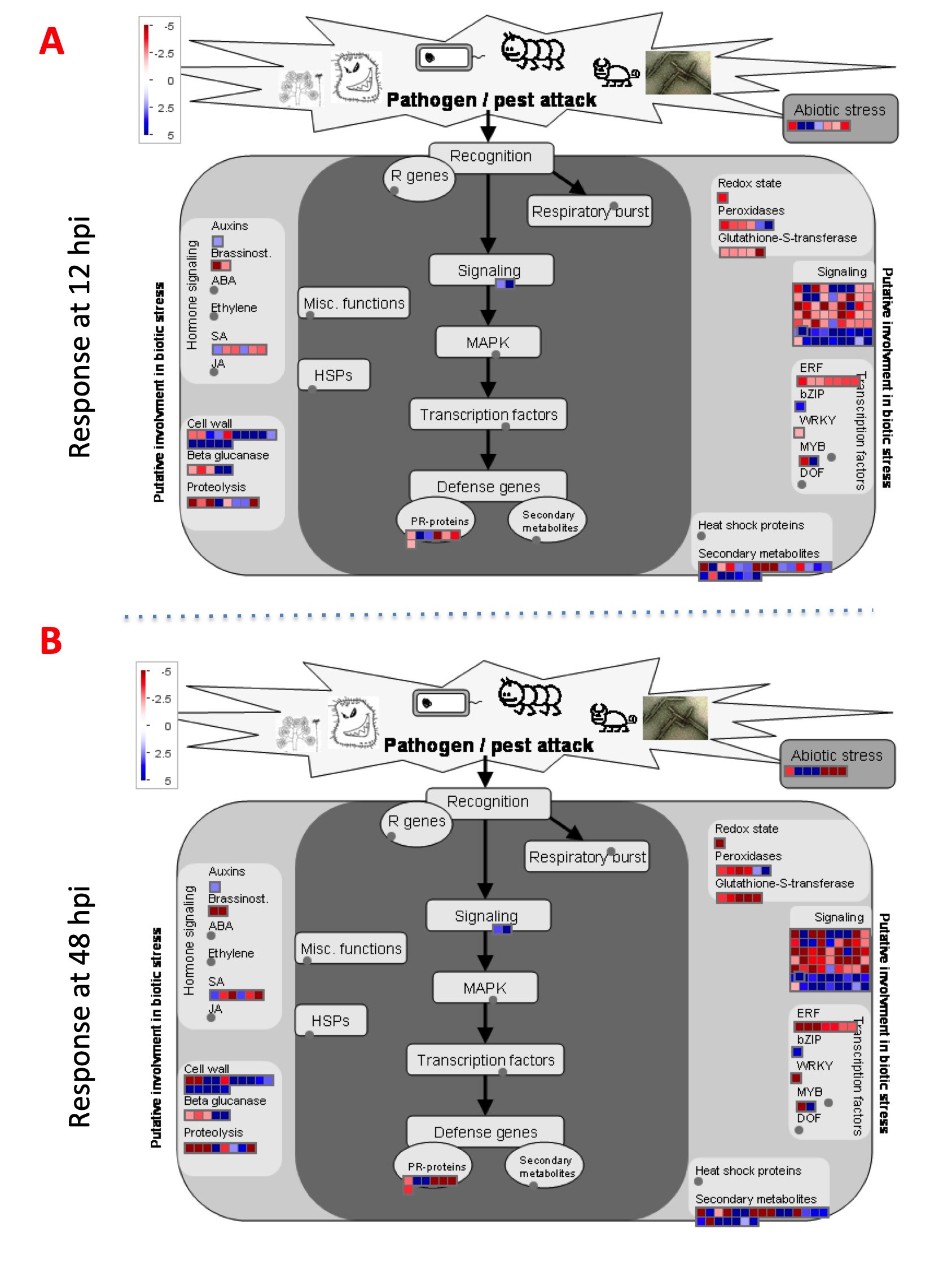


**Supplementary Figure 9.** MapMan overview showing differentially expressed common (common between 12 hpi and 48 hpi) genes related to biotic and abiotic stress in M4 line at 12 hpi (A) and 48 hpi (B). Up-regulated genes are shown by light to deep blue color boxes and the down-regulated genes are shown by light to deep red color boxes. Color intensity show the level of expressions as indicated by the intensity bar on top left of each panel. Genes involved in same functions are clubbed together. Grey dots indicate that there were no significant expressions of such genes.


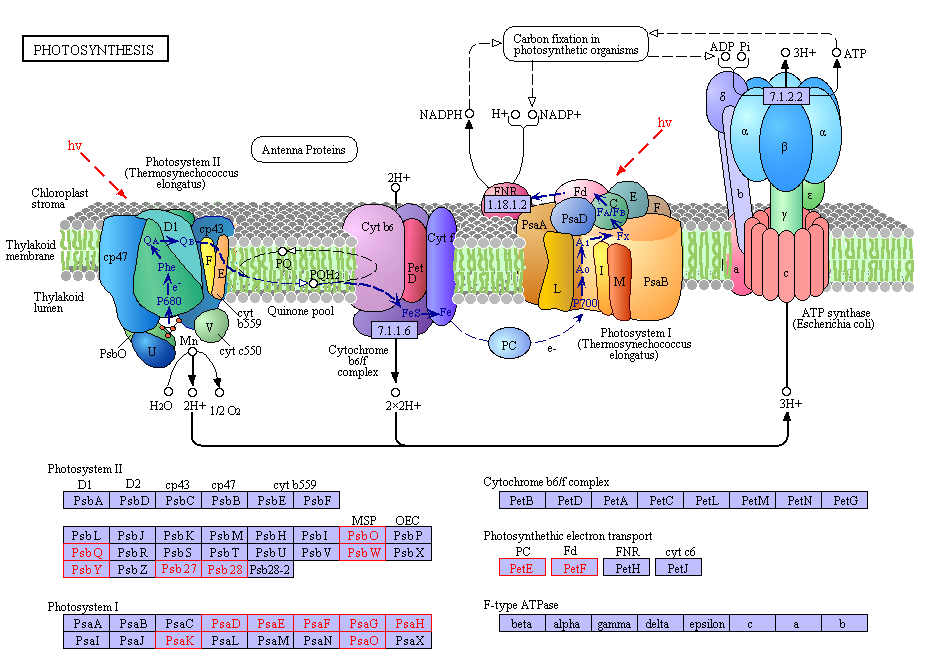


**Supplementary Figure 10.** Kyoto Encyclopedia of Genes and Genomes (KEGG) pathway map (42) analysis of up-regulated genes involved in photosynthesis pathways. Photosynthesis is one of the significant pathways detected after Fusarium inoculation and shows increased energy requirement to fight Fusarium infection. Up-regulated genes (log2 fold change ≥ 2) are shown in red boxes and are also given below, whereas, the black boxes show the genes that are not affected by the infection.


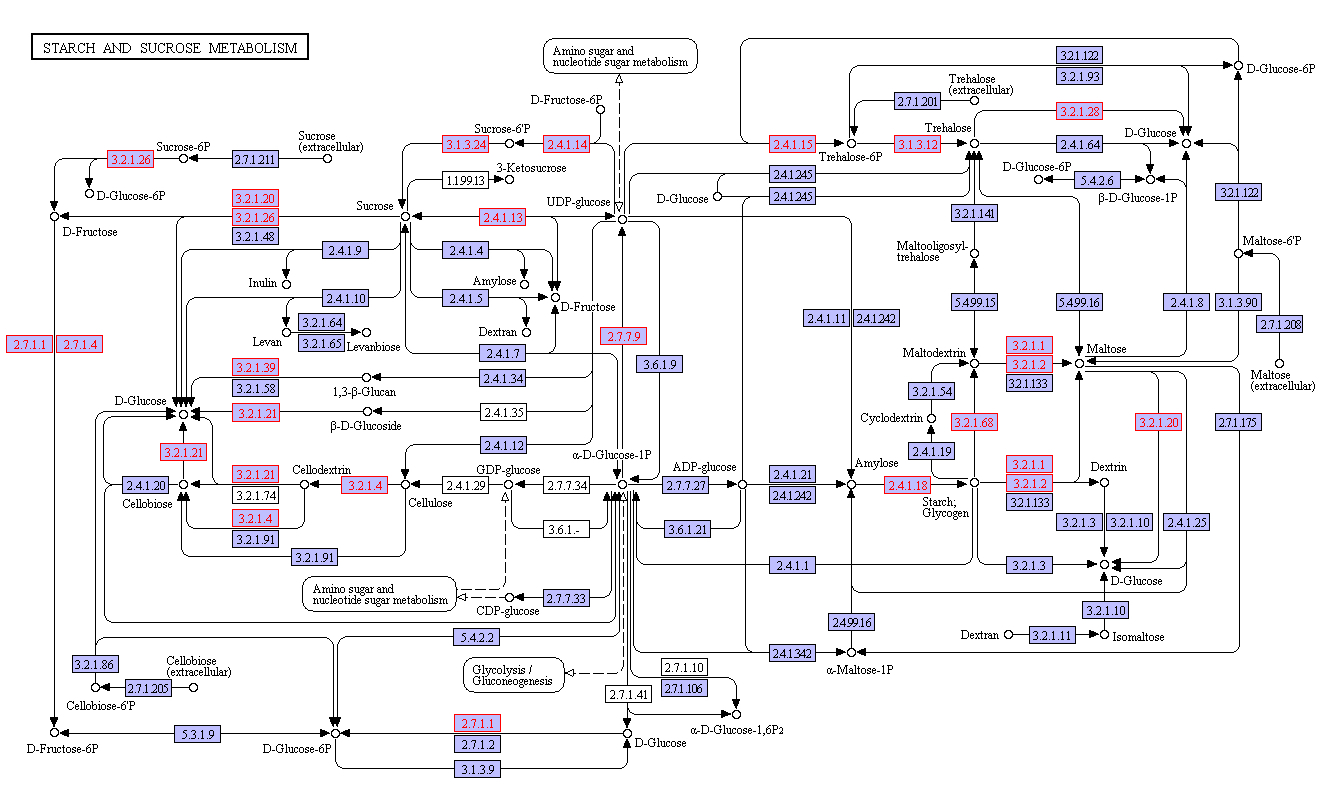


**Supplementary Figure 11**. Kyoto Encyclopedia of Genes and Genomes (KEGG) pathway map (42) analysis of DEGs. Starch and sucrose metabolism is one of the significant pathways detected after Fusarium inoculation. DEGs (log2 fold change ≥ 2 or ≤ - 2) are shown in red boxes, whereas, the black boxes show the genes that are not affected by the infection.


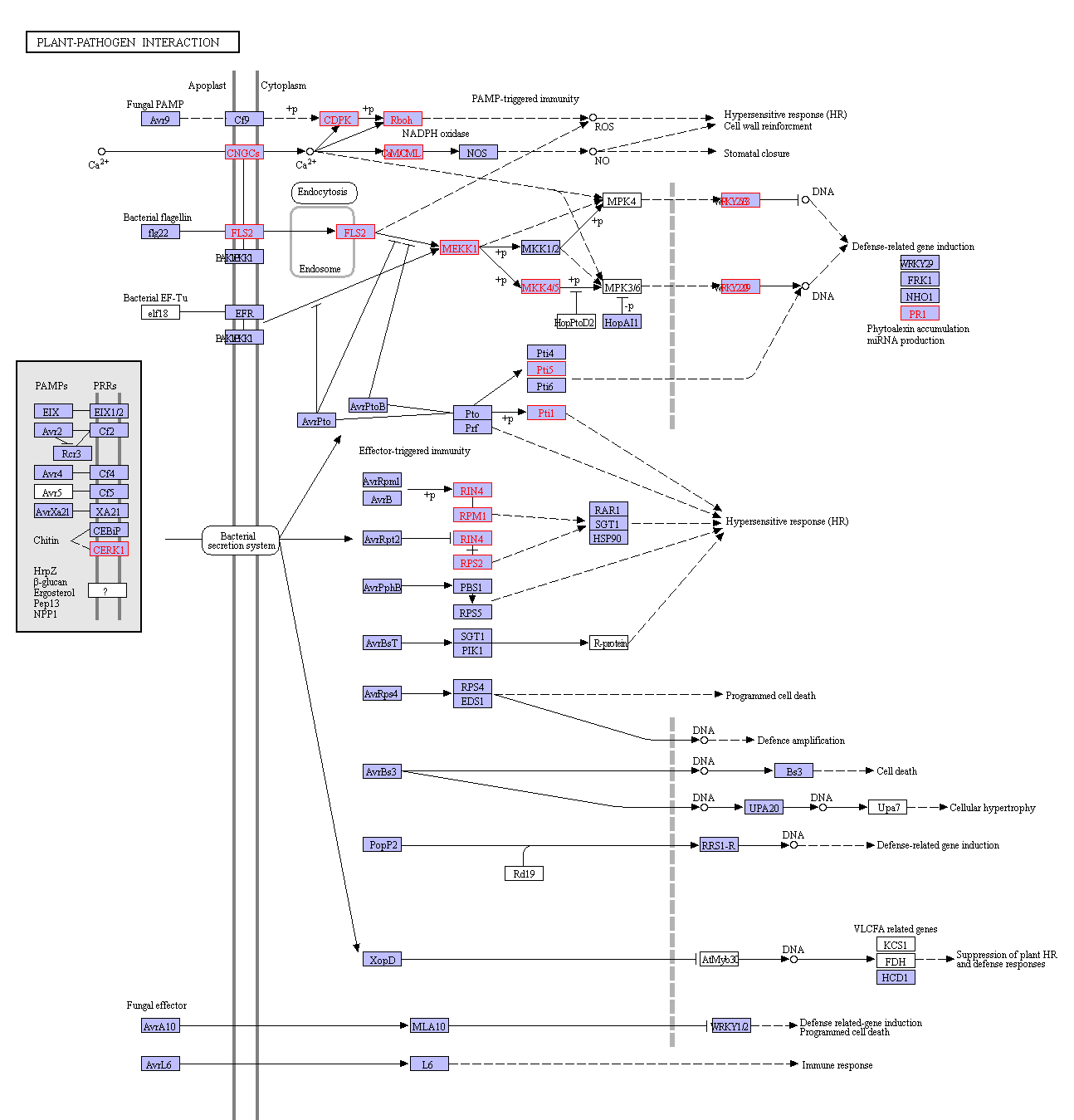


**Supplementary Figure 12**. Kyoto Encyclopedia of Genes and Genomes (KEGG) pathway map (42) analysis of DEGs involved in plant-pathogen interaction (PPI). PPI is one of the significant pathways detected after Fusarium inoculation and determines resistance or susceptibility. Significant DEGs (log2 fold change ≥ 2 or ≤ - 2) are shown in red boxes, while the genes with non-significant expression (≥ - 2 or ≤ 2) are shown in black boxes.


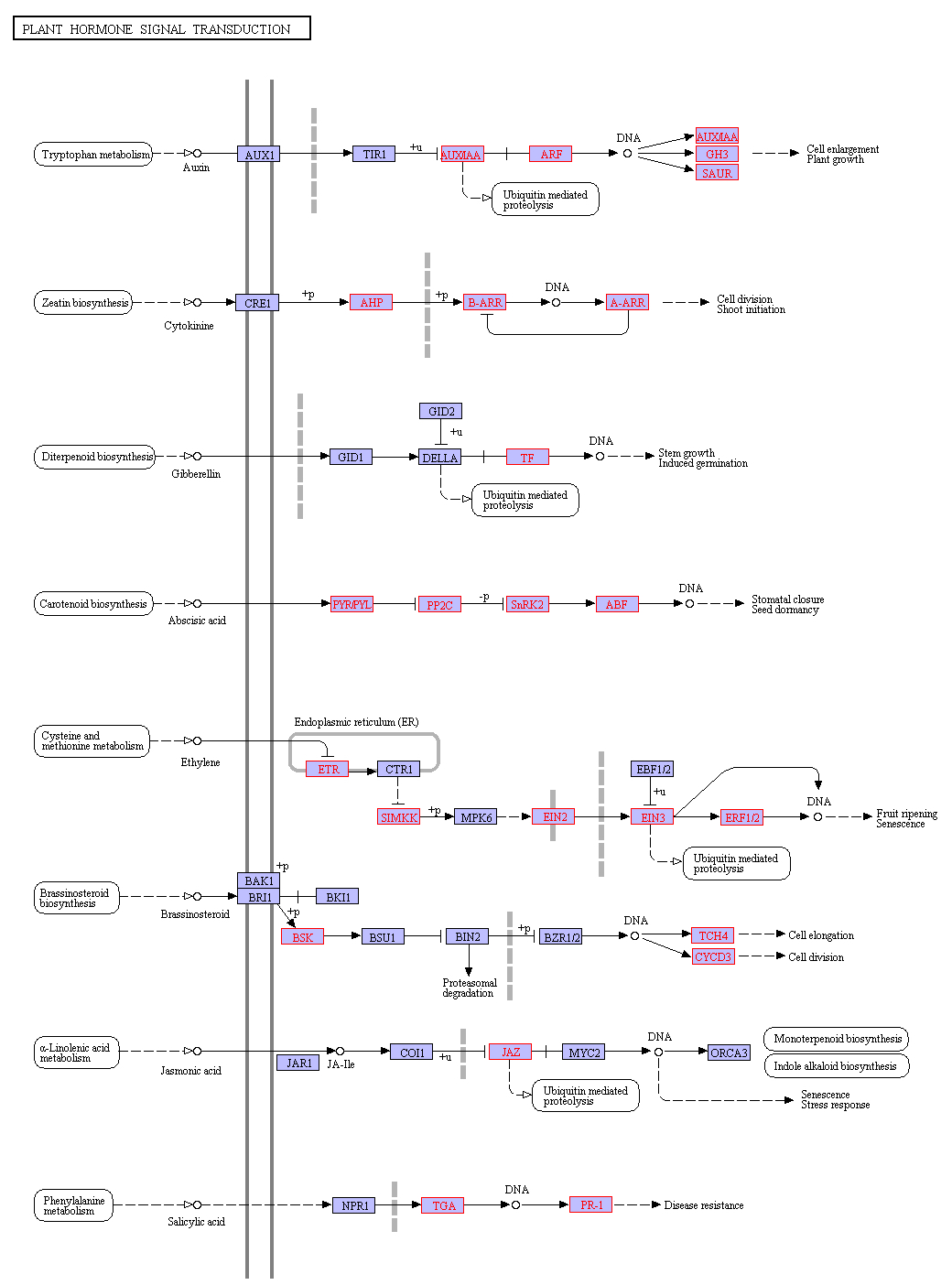


**Supplementary Figure 13**. Kyoto Encyclopedia of Genes and Genomes (KEGG) pathway map (42) analysis of DEGs involved in hormone signal transduction, which is one of the significant pathways detected after Fusarium inoculation, and determines resistance or susceptibility. Significant DEGs (log2 fold change ≥ 2 or ≤ - 2) are shown in red boxes, while the genes with non-significant expression (≥ - 2 or ≤ 2) are shown in black boxes.

**
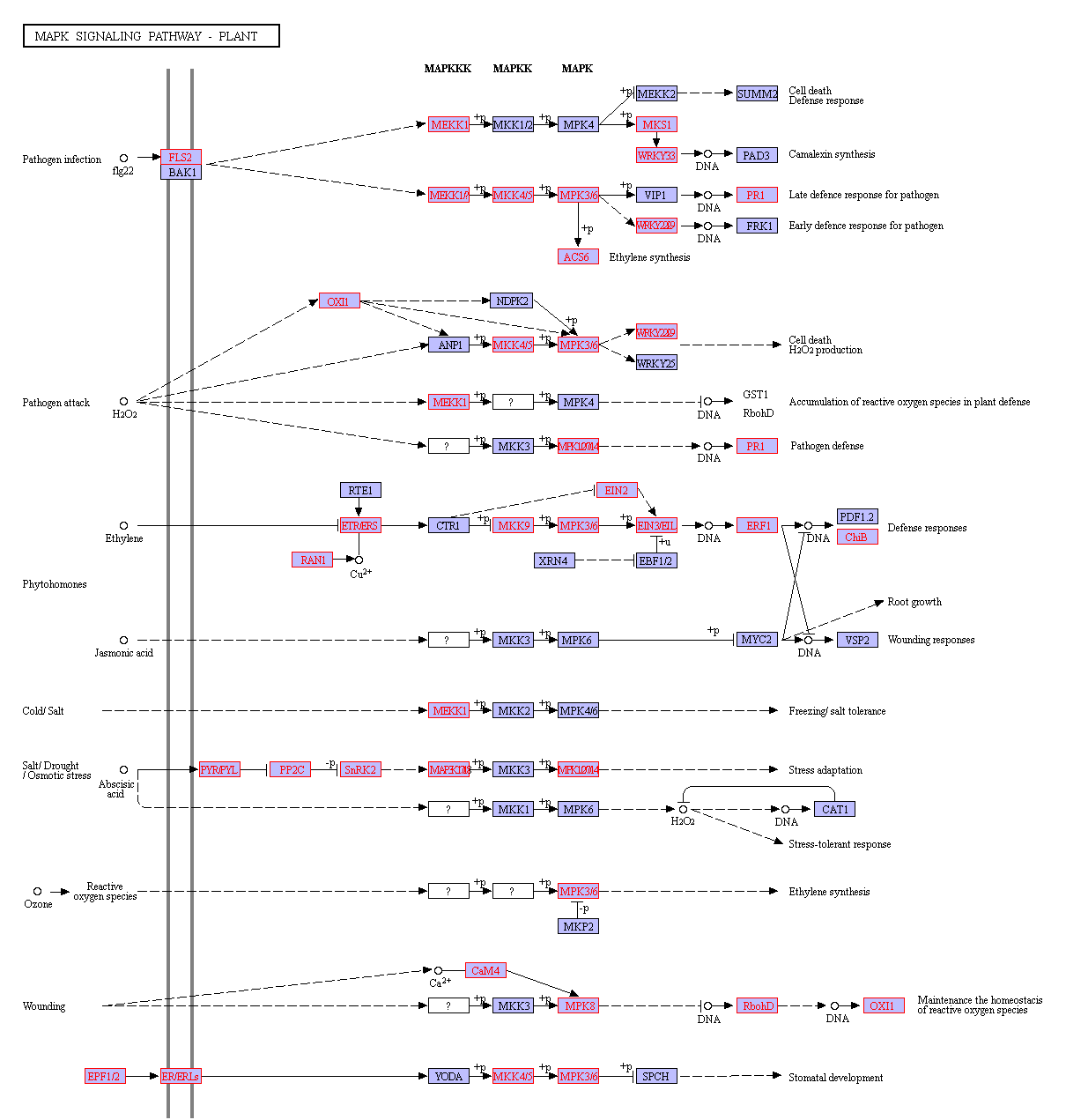
**

**Supplementary Figure 14.** Kyoto Encyclopedia of Genes and Genomes (KEGG) pathway map (42) analysis of DEGs involved in MAPK signaling pathway. Significant DEGs (log2 fold change ≥ 2 or ≤ -2) are shown in red boxes, while the genes with non-significant expression (≥ -2 or ≤ 2) are shown in black boxes.

**Supplementary Table 1**. List of primers used in real-time PCR.

| **Ensembl Gene ID** | **Primers** | **Sequence** |
| --- | --- | --- |
| 1Bv1G181990 | 1Bv1G181990F | ATCCTGATGGTTGCCTTCG |
|  | 1Bv1G181990R | CGTCGTCGTTGTCCTCTTC |
| 7Av1G000890 | 7Av1G000890F | CAAGATCTCCCAGGAGGAGTA |
|  | 7Av1G000890R | GCAGTAGAAGCCGTAGGTG |
| 3Av1G224650 | 3Av1G224650F | CCCAAACACTCCATTCACTCT |
|  | 3Av1G224650R | GGCGAGAGCTTTCATCACT |
| 2Av1G137870 | 2Av1G137870F | GCTCCTCCACCAACATCATC |
|  | 2Av1G137870R | GCCGTAGCCTTCCTGTTC |
| 5Av1G227400 | 5Av1G227400F | CAAGATCCGCAACAAGGAGAT |
|  | 5Av1G227400R | GCCATGTAGATGCCGTTCTT |
| 2Bv1G148630 | 2Bv1G148630F | GCCTTCTCTACCAGCTTCAC |
|  | 2Bv1G148630R | TGATCCTGCCGTTCCTTTC |
| 1Av1G005170 | 1Av1G005170F | CATCGAATCCCTTGGATCTCTC |
|  | 1Av1G005170R | CCTGTTGTGCATCTCCTTCT |
| 6Av1G226100 | 6Av1G226100F | TCAACTGATGGATCTGCTAGTG |
|  | 6Av1G226100R | GCTTCTGTAGGCCTTCTTTCT |
| 7Av1G266500 | 7Av1G266500F | AAAGAGTTCTCGAGGCTGAC |
|  | 7Av1G266500R | ACGCGTACATCCCGAAC |
| 5Av1G219320 | 5Av1G219320F | TGGGTATCGGAGAAGCAGTA |
|  | 5Av1G219320R | CCACCTGTGTGTAGTGCAA |
| 7Av1G266530 | 7Av1G266530F | ACGGCAAGGTTTCTGTGT |
|  | 7Av1G266530R | CCACCGCTGCAAGGATTA |
| 6Bv1G226900 | 6Bv1G226900F | TTGCACCAGTCTTGGGTATC |
|  | 6Bv1G226900R | GCTAGCCGGGTAGATTCTTTAG |
| 2Bv1G189070 | 2Bv1G189070F | GAGGATCTTCGTGGGCTAATC |
|  | 2Bv1G189070R | TGATGCCAAGGGCTTCTATC |
| 7Bv1G218880 | 7Bv1G218880F | TGGGTTGCTAAACCAAGAAGA |
|  | 7Bv1G218880R | CGACGTGTACATCCCGAAC |

**Supplementary Table 2.** Tukey HSD differences of the visual score from Saint Paul, MN field season 2015, along with means, standard errors, and confidence intervals.

| **Lines** | **Estimate*** | **Std. Error** | **Lower** | **Upper** | **Differences** |
| --- | --- | --- | --- | --- | --- |
| 41708.72 | 24.16 | 4.72 | 14.92 | 33.41 | b |
| E.25.10 | 30.23 | 4.72 | 20.99 | 39.48 | ab |
| 4581.8.B | 30.29 | 4.72 | 21.04 | 39.54 | ab |
| E.25.11 | 31.62 | 5.78 | 20.30 | 42.95 | ab |
| E.25.32 | 31.63 | 5.78 | 20.31 | 42.96 | ab |
| D0.3028 | 31.85 | 5.78 | 20.52 | 43.17 | ab |
| 4581.1 | 32.89 | 4.72 | 23.64 | 42.14 | ab |
| D0.4581 | 35.81 | 5.78 | 24.49 | 47.14 | abc |
| 4581.6 | 36.11 | 4.72 | 26.86 | 45.35 | abc |
| 4581.8.A | 36.15 | 4.72 | 26.90 | 45.40 | abc |
| E.25.26 | 36.15 | 5.78 | 24.83 | 47.48 | abc |
| D0.3708 | 37.26 | 5.78 | 25.93 | 48.58 | abc |
| E.25.20 | 37.48 | 5.78 | 26.16 | 48.81 | abc |
| TRT.4.19 | 37.94 | 5.78 | 26.61 | 49.26 | abc |
| E.25 | 39.01 | 5.78 | 27.69 | 50.34 | abc |
| D0.41708 | 39.30 | 5.78 | 27.97 | 50.63 | abc |
| 3028.1 | 40.69 | 4.72 | 31.45 | 49.94 | abc |
| Alkabo | 40.80 | 5.78 | 29.47 | 52.12 | abc |
| Divide | 40.96 | 5.78 | 29.64 | 52.29 | abc |
| TRT.4.36 | 41.11 | 5.78 | 29.78 | 52.43 | abc |
| 3708.8.old | 42.00 | 4.72 | 32.76 | 51.25 | abc |
| Monroe | 42.37 | 8.17 | 26.36 | 58.39 | abc |
| D0.6710 | 42.41 | 5.78 | 31.08 | 53.73 | abc |
| E.25.23 | 42.56 | 5.78 | 31.24 | 53.89 | abc |
| Grenora | 42.92 | 5.78 | 31.59 | 54.24 | abc |
| E.25.21 | 43.35 | 5.78 | 32.03 | 54.68 | abc |
| D0.6855 | 43.59 | 5.78 | 32.26 | 54.91 | abc |
| Lebsock | 44.00 | 5.78 | 32.67 | 55.33 | abc |
| 3708.8.C | 44.30 | 4.72 | 35.05 | 53.55 | abc |
| TB 17.1 | 44.92 | 5.78 | 33.59 | 56.24 | abc |
| 6855.55 | 45.59 | 4.72 | 36.35 | 54.84 | abc |
| 3708.4 | 45.89 | 4.72 | 36.64 | 55.14 | abc |
| 3708.7 | 46.06 | 4.72 | 36.82 | 55.31 | abc |
| TB 16.2 | 46.86 | 5.78 | 35.53 | 58.18 | abc |
| 6710.8 | 47.28 | 4.72 | 38.04 | 56.53 | abc |
| 3708.8.B | 47.34 | 4.72 | 38.09 | 56.59 | abc |
| 3708.2 | 47.57 | 4.72 | 38.32 | 56.82 | abc |
| TRT.4 | 48.66 | 5.78 | 37.33 | 59.98 | abc |
| 3708.8.A | 49.61 | 4.72 | 40.37 | 58.86 | abc |
| 3708.C | 51.68 | 5.78 | 40.36 | 63.01 | abc |
| 41708.31 | 52.70 | 4.72 | 43.45 | 61.95 | a c |
| TB 16.1 | 52.87 | 5.78 | 41.54 | 64.19 | abc |
| 6855.25 | 54.37 | 4.72 | 45.12 | 63.61 | a c |
| TB 16.3 | 55.00 | 5.78 | 43.67 | 66.32 | abc |
| TB 16.4 | 66.27 | 5.78 | 54.94 | 77.59 | c |

*Estimates are generated using visual scores of three biological replicates in R. Each biological replicates contained 20 spikes.

**Supplementary Table 3.** Tukey HSD differences for the visual score from Fargo, ND field season 2015, along with means, standard errors, and confidence intervals.

| **Lines** | **Estimate*** | **Std. Error** | **Lower** | **Upper** | **Differences** |
| --- | --- | --- | --- | --- | --- |
| Alsen | 16.94 | 9.09 | 0.88 | 34.75 | b |
| E.25 | 27.18 | 6.43 | 14.58 | 39.77 | ab |
| E.25.26 | 29.47 | 6.43 | 16.87 | 42.07 | abc |
| E.25.11 | 31.97 | 6.43 | 19.37 | 44.57 | abcd |
| E.25.23 | 34.94 | 6.43 | 22.34 | 47.54 | abcd |
| E.25.21 | 36.42 | 4.54 | 27.51 | 45.33 | abc |
| 3708.C | 36.84 | 6.43 | 24.25 | 49.44 | abcde |
| E.25.10 | 38.71 | 5.25 | 28.42 | 48.99 | abcd |
| E.25.32 | 38.75 | 6.43 | 26.15 | 51.35 | abcdef |
| 41708.72 | 40.58 | 5.25 | 30.30 | 50.87 | abcde |
| 3708.2 | 44.63 | 5.25 | 34.34 | 54.91 | abcdefg |
| D0.41708 | 45.10 | 6.43 | 32.50 | 57.69 | abcdefgh |
| TB 16.2 | 47.69 | 6.43 | 35.09 | 60.29 | abcdefgh |
| 3708.4 | 49.52 | 5.25 | 39.23 | 59.81 | abcdefgh |
| 4581.1 | 49.54 | 5.25 | 39.26 | 59.83 | abcdefgh |
| 41708.31 | 50.42 | 5.25 | 40.13 | 60.70 | abcdefgh |
| 4581.6 | 50.52 | 5.25 | 40.23 | 60.81 | abcdefgh |
| TRT.4 | 50.56 | 6.43 | 37.96 | 63.16 | abcdefgh |
| 3708.8.old | 50.77 | 5.25 | 40.48 | 61.06 | abcdefgh |
| 3708.8.B | 50.98 | 5.25 | 40.69 | 61.27 | abcdefgh |
| TRT.4.36 | 51.63 | 6.43 | 39.03 | 64.22 | abcdefgh |
| 6855.25 | 51.75 | 5.25 | 41.46 | 62.04 | abcdefgh |
| 3708.8.A | 51.79 | 5.25 | 41.51 | 62.08 | abcdefgh |
| D0.3708 | 52.31 | 6.43 | 39.71 | 64.91 | abcdefgh |
| D0.3028 | 52.50 | 6.43 | 39.90 | 65.10 | abcdefgh |
| TB 17.1 | 52.60 | 7.42 | 38.05 | 67.14 | abcdefgh |
| D0.4581 | 52.91 | 6.43 | 40.31 | 65.50 | abcdefgh |
| 3708.7 | 53.50 | 5.25 | 43.21 | 63.79 | abcdefgh |
| TRT.4.19 | 55.44 | 6.43 | 42.84 | 68.04 | abcdefgh |
| 4581.8.A | 56.39 | 5.25 | 46.11 | 66.68 | abcdefgh |
| D0.6855 | 56.50 | 6.43 | 43.90 | 69.10 | abcdefgh |
| 3028.1 | 57.90 | 5.25 | 47.61 | 68.18 | abcdefgh |
| 6855.55 | 57.90 | 5.25 | 47.61 | 68.18 | abcdefgh |
| 4581.8.B | 58.84 | 5.25 | 48.55 | 69.12 | a cdefgh |
| 3708.8.C | 59.42 | 5.25 | 49.13 | 69.70 | a cdefgh |
| TB 16.1 | 65.28 | 6.43 | 52.68 | 77.88 | cdefgh |
| TB 16.3 | 68.00 | 6.43 | 55.40 | 80.60 | defgh |
| 6710.8 | 70.33 | 5.25 | 60.05 | 80.62 | fgh |
| D0.6710 | 76.56 | 6.43 | 63.96 | 89.16 | gh |
| TB 16.4 | 77.81 | 6.43 | 65.21 | 90.41 | h |
| MN00269 | 80.75 | 9.09 | 62.93 | 98.57 | efgh |

*Estimates are generated using visual scores of three biological replicates in R. Each biological replicates contained 20 spikes.

**Supplementary Table 4.** Fusarium damaged kernel (FDK) and deoxynivalenol (DON) values of the seven best (5 M4 lines in yellow and 2 parental lines in light green) and seven worst (not highlighted) performing lines during the 2015 summer field trial at Saint Paul, MN and Fargo, ND.

| **Saint Paul, MN** | | | | **Fargo, ND** | | | |
| --- | --- | --- | --- | --- | --- | --- | --- |
| **Lines** | **% FDK values*** | **Lines** | **DON (ppm)*** | **Lines** | **% FDK values*** | **Lines** | **DON (ppm)*** |
| 41708-72 | 42 | 41708-72 | 5.6 | 41708-72 | 33 | 41708-72 | 11.08 |
| D0-41708 | 51 | D0-41708 | 6.5 | D0-41708 | 31 | D0-41708 | 8.83 |
| E-25-10 | 39 | E-25-10 | 6.6 | E-25-10 | 17 | E-25-10 | 6.45 |
| E-25-11 | 26 | E-25-11 | 3.15 | E-25-11 | 16 | E-25-11 | 7.75 |
| E-25-23 | 23 | E-25-23 | 2.7 | E-25-23 | 17 | E-25-23 | 5.53 |
| E-25-32 | 26 | E-25-32 | 2.95 | E-25-32 | 23 | E-25-32 | 5.53 |
| E-25 | 40 | E-25 | 2.46 | E-25 | 19 | E-25 | 6.08 |
|  |  |  |  |  |  |  |  |
| 3708-C | 48 | TB 17-1 | 10.9 | D0-3708 | 40 | 6710-8 | 15.85 |
| 6855-25 | 49 | D0-3708 | 11.4 | D0-6710 | 40 | TB 16-3 | 16.3 |
| TB 16-3 | 49 | 3028-10 | 11.53 | 41708-31 | 41 | D0-4581 | 16.4 |
| 3708-8-old | 50 | 3708-7 | 12.17 | 4581-8-A | 41 | 3708-4 | 18.38 |
| TB 17-1 | 52 | 3708-8-C | 13.13 | TB 16-1 | 45 | TB 16-1 | 19.18 |
| 3708-8-C | 53 | 3708-8-old | 13.37 | TB 16-3 | 47 | D0-6710 | 19.35 |
| TB 16-4 | 54 | 3708-8-B | 18.27 | TB 16-4 | 58 | D0-3708 | 19.58 |

*Mean value of three biological replicates. Alsen and MN00269 are the FHB resistant and susceptible checks.

**Supplementary Table 5.** Visual score, fusarium damaged kernel (FDK) and deoxynivalenol (DON) values of the seven best (5 M4 lines in yellow and 2 parental lines in light green) and seven worst (not highlighted) performing lines during the 2016 summer field trial at Saint Paul, MN.

| **Visual Score** | | | **FDK** | | **DON** | |
| --- | --- | --- | --- | --- | --- | --- |
| **Lines** | **Score*** | **Std. Err.** | **Lines** | **% FDK value** | **Lines** | **DON (ppm)** |
| 41708-72 | 18.25 | 0.0395 | 41708-72 | 5.67% | 41708-72 | 1.9 |
| D0-41708 | 21.90 | 0.0202 | D0-41708 | 3.33% | D0-41708 | 3 |
| E-25-10 | 15.97 | 0.0261 | E-25-10 | 6.33% | E-25-10 | 2 |
| E-25-11 | 18.48 | 0.0099 | E-25-11 | 6.00% | E-25-11 | 2.4 |
| E-25-23 | 17.04 | 0.0124 | E-25-23 | 8.33% | E-25-23 | 1.4 |
| E-25-32 | 16.57 | 0.0343 | E-25-32 | 6.33% | E-25-32 | 2.3 |
| E-25 | 16.41 | 0.022 | E-25 | 2.67% | E-25 | 2.2 |
|  |  |  |  |  |  |  |
| 4581-10 | 19.31 | 0.0306 | TB 16-3 | 8.00% | 3708-4 | 3.7 |
| D0-4581 | 19.34 | 0.0281 | TB 17-1 | 6.00% | 41708-31 | 4 |
| 41708-31 | 19.40 | 0.0196 | 6855-25 | 6.67% | TRT-4-19 | 4.3 |
| 6710-8 | 19.93 | 0.0393 | E-25-26 | 11.00% | 6710-8 | 4.4 |
| 4581-8-B | 20.11 | 0.0456 | 4581-10 | 9.33% | D0-6710 | 5.1 |
| TB 16-1 | 20.67 | 0.0457 | D0-4581 | 15.67% | TB 16-2 | 5.6 |
| 4581-6 | 22.15 | 0.0563 | 4581-6 | 13.67% | D0-3708 | 7 |
|  |  |  |  |  |  |  |
| Alsen | 8.09 | 1.11 | Alsen | 2.50% | Alsen | 0.9 |
| MN00269 | 9.98 | 0.26 | MN00269 | 9.00% | MN00269 | 3 |

*Average of three biological replicates. Each biological replicates are average of score from 20 spikes. Alsen and MN00269 are the FHB resistant and susceptible checks.

**Supplementary Table 6.** Visual score of field and greenhouse inoculations and deoxynivalenol (DON) values from the field inoculations of the seven best (5 M lines in yellow and 2 parental lines in light green) and seven worst (not highlighted) performing lines during the 2017 summer field and Fall greenhouse seasons at Saint Paul, MN.

| **Field*** | | **Green House*** | | **DON analysis*** | |  |
| --- | --- | --- | --- | --- | --- | --- |
| **Lines** | **% Infection** | **Lines** | **% Infection** | **Lines** | **DON (ppm)** | |
| 41708-72 | 12.73% | 41708-72 | 14.84% | 41708-72 | 3.4 | |
| D0-41708 | 12.05% | D0-41708 | 6.98% | D0-41708 | 7.9 | |
| E-25-10 | 11.97% | E-25-10 | 5.25% | E-25-10 | 2.7 | |
| E-25-11 | 10.14% | E-25-11 | 7.31% | E-25-11 | 5.2 | |
| E-25-23 | 7.13% | E-25-23 | 5.43% | E-25-23 | 2.3 | |
| E-25-32 | 13.58% | E-25-32 | 12.22% | E-25-32 | 3.6 | |
| E-25 | 10.87% | E-25 | 6.73% | E-25 | 4.5 | |
|  |  |  |  |  |  | |
| TRT-4-19 | 14.89% | TRT-4-19 | 12.83% | 41708-31 | 7.8 | |
| TB 16-1 | 15.05% | 3708-8-old | 13.48% | D0-3028 | 7.8 | |
| 4581-8-A | 15.14% | 4581-8-A | 14.47% | 3708-8-old | 7.8 | |
| 4581-10 | 15.85% | TB 16-3 | 16.01% | 3708-C | 8.2 | |
| 41708-31 | 16.67% | 4581-10 | 16.79% | 6855-25 | 8.3 | |
| TB 16-3 | 17.04% | TB 16-4 | 16.89% | 6710-8 | 9.6 | |
| TB 16-2 | 18.25% | TB 16-2 | 19.08% | 4581-10 | 9.8 | |
|  |  |  |  |  |  | |
|  |  | Alsen | 3.44% | Alsen | 0.8 | |
|  |  | MN00269 | 19.87% | MN00269 | 4.8 | |

*The data shown are average value of three biological replicates. Alsen and MN00269 are the FHB resistant and susceptible checks.

**Supplementary Table 7.** Enrichment of GO terms that represents up-regulated genes common to 12 h and 48 h upon *Fusarium* inoculations.

| **GO accession** | **Term type** | **Term** | **Query item** | **Query total** | **bgitem** | **bgtotal** | **pvalue** |
| --- | --- | --- | --- | --- | --- | --- | --- |
| GO:0042545 | P | cell wall modification | 5 | 187 | 178 | 144935 | 4.50E-06 |
| GO:0071555 | P | cell wall organization | 6 | 187 | 386 | 144935 | 1.40E-05 |
| GO:0071554 | P | cell wall organization or biogenesis | 7 | 187 | 660 | 144935 | 2.90E-05 |
| GO:0006633 | P | fatty acid biosynthetic process | 6 | 187 | 489 | 144935 | 5.00E-05 |
| GO:0006952 | P | defense response | 5 | 187 | 362 | 144935 | 0.00012 |
| GO:0006631 | P | fatty acid metabolic process | 6 | 187 | 735 | 144935 | 0.00044 |
| GO:0016053 | P | organic acid biosynthetic process | 7 | 187 | 1233 | 144935 | 0.0012 |
| GO:0046394 | P | carboxylic acid biosynthetic process | 7 | 187 | 1233 | 144935 | 0.0012 |
| GO:0032787 | P | monocarboxylic acid metabolic process | 6 | 187 | 1113 | 144935 | 0.0035 |
| GO:0008610 | P | lipid biosynthetic process | 7 | 187 | 1647 | 144935 | 0.006 |
| GO:0044255 | P | cellular lipid metabolic process | 8 | 187 | 2348 | 144935 | 0.012 |
| GO:0044283 | P | small molecule biosynthetic process | 7 | 187 | 2069 | 144935 | 0.019 |
| GO:0016192 | P | vesicle-mediated transport | 6 | 187 | 1653 | 144935 | 0.021 |
| GO:0043687 | P | post-translational protein modification | 28 | 187 | 14897 | 144935 | 0.028 |
| GO:0006468 | P | protein amino acid phosphorylation | 26 | 187 | 13679 | 144935 | 0.03 |
| GO:0006629 | P | lipid metabolic process | 10 | 187 | 4142 | 144935 | 0.044 |
| GO:0006464 | P | protein modification process | 28 | 187 | 16098 | 144935 | 0.063 |
| GO:0006796 | P | phosphate metabolic process | 27 | 187 | 15620 | 144935 | 0.072 |
| GO:0006793 | P | phosphorus metabolic process | 27 | 187 | 15620 | 144935 | 0.072 |
| GO:0016310 | P | phosphorylation | 26 | 187 | 15144 | 144935 | 0.081 |
| GO:0006950 | P | response to stress | 8 | 187 | 4184 | 144935 | 0.18 |
| GO:0019752 | P | carboxylic acid metabolic process | 7 | 187 | 3777 | 144935 | 0.22 |
| GO:0042180 | P | cellular ketone metabolic process | 7 | 187 | 3797 | 144935 | 0.22 |
| GO:0044249 | P | cellular biosynthetic process | 12 | 187 | 18890 | 144935 | 1 |
| GO:0044281 | P | small molecule metabolic process | 9 | 187 | 8653 | 144935 | 0.79 |
| GO:0050789 | P | regulation of biological process | 5 | 187 | 9518 | 144935 | 0.99 |
| GO:0044267 | P | cellular protein metabolic process | 30 | 187 | 23103 | 144935 | 0.52 |
| GO:0044260 | P | cellular macromolecule metabolic process | 33 | 187 | 38134 | 144935 | 1 |
| GO:0016043 | P | cellular component organization | 5 | 187 | 4641 | 144935 | 0.72 |
| GO:0051179 | P | localization | 9 | 187 | 12991 | 144935 | 0.99 |
| GO:0065007 | P | biological regulation | 7 | 187 | 10277 | 144935 | 0.98 |
| GO:0006508 | P | proteolysis | 9 | 187 | 4923 | 144935 | 0.19 |
| GO:0009987 | P | cellular process | 62 | 187 | 64217 | 144935 | 1 |
| GO:0009058 | P | biosynthetic process | 13 | 187 | 20626 | 144935 | 1 |
| GO:0006810 | P | transport | 9 | 187 | 12862 | 144935 | 0.99 |
| GO:0043412 | P | macromolecule modification | 28 | 187 | 17042 | 144935 | 0.11 |
| GO:0008152 | P | metabolic process | 83 | 187 | 73359 | 144935 | 0.96 |
| GO:0043436 | P | oxoacid metabolic process | 7 | 187 | 3777 | 144935 | 0.22 |
| GO:0051234 | P | establishment of localization | 9 | 187 | 12862 | 144935 | 0.99 |
| GO:0009056 | P | catabolic process | 6 | 187 | 3313 | 144935 | 0.26 |
| GO:0009057 | P | macromolecule catabolic process | 6 | 187 | 2625 | 144935 | 0.13 |
| GO:0055114 | P | oxidation reduction | 16 | 187 | 11488 | 144935 | 0.41 |
| GO:0044238 | P | primary metabolic process | 62 | 187 | 53794 | 144935 | 0.88 |
| GO:0005975 | P | carbohydrate metabolic process | 13 | 187 | 7421 | 144935 | 0.16 |
| GO:0006082 | P | organic acid metabolic process | 7 | 187 | 3796 | 144935 | 0.22 |
| GO:0019538 | P | protein metabolic process | 36 | 187 | 26987 | 144935 | 0.44 |
| GO:0050896 | P | response to stimulus | 10 | 187 | 5156 | 144935 | 0.13 |
| GO:0044237 | P | cellular metabolic process | 46 | 187 | 49113 | 144935 | 1 |
| GO:0043170 | P | macromolecule metabolic process | 43 | 187 | 42476 | 144935 | 0.98 |
| GO:0030599 | F | pectinesterase activity | 12 | 187 | 269 | 144935 | 4.30E-15 |
| GO:0004857 | F | enzyme inhibitor activity | 11 | 187 | 345 | 144935 | 2.00E-12 |
| GO:0030234 | F | enzyme regulator activity | 17 | 187 | 1452 | 144935 | 1.20E-11 |
| GO:0004091 | F | carboxylesterase activity | 12 | 187 | 794 | 144935 | 8.70E-10 |
| GO:0016788 | F | hydrolase activity, acting on ester bonds | 16 | 187 | 3945 | 144935 | 6.30E-05 |
| GO:0046872 | F | metal ion binding | 36 | 187 | 15190 | 144935 | 0.00025 |
| GO:0005083 | F | small GTPase regulator activity | 6 | 187 | 665 | 144935 | 0.00026 |
| GO:0043167 | F | ion binding | 36 | 187 | 15344 | 144935 | 0.0003 |
| GO:0043169 | F | cation binding | 36 | 187 | 15343 | 144935 | 0.0003 |
| GO:0030695 | F | GTPase regulator activity | 6 | 187 | 708 | 144935 | 0.00036 |
| GO:0060589 | F | nucleoside-triphosphatase regulator activity | 6 | 187 | 748 | 144935 | 0.00048 |
| GO:0046914 | F | transition metal ion binding | 26 | 187 | 10265 | 144935 | 0.00079 |
| GO:0005506 | F | iron ion binding | 12 | 187 | 3588 | 144935 | 0.0026 |
| GO:0016747 | F | transferase activity, transferring acyl groups other than amino-acyl groups | 6 | 187 | 1445 | 144935 | 0.012 |
| GO:0005509 | F | calcium ion binding | 6 | 187 | 1474 | 144935 | 0.013 |
| GO:0016746 | F | transferase activity | 7 | 187 | 2002 | 144935 | 0.016 |
| GO:0020037 | F | heme binding | 9 | 187 | 3260 | 144935 | 0.027 |
| GO:0016773 | F | phosphotransferase activity, alcohol group as acceptor | 29 | 187 | 15564 | 144935 | 0.028 |
| GO:0004553 | F | hydrolase activity, hydrolyzing O-glycosyl compounds | 9 | 187 | 3355 | 144935 | 0.031 |
| GO:0004672 | F | protein kinase activity | 26 | 187 | 13823 | 144935 | 0.033 |
| GO:0016740 | F | transferase activity | 47 | 187 | 28658 | 144935 | 0.043 |
| GO:0046906 | F | tetrapyrrole binding | 9 | 187 | 3528 | 144935 | 0.041 |
| GO:0016798 | F | hydrolase activity, acting on glycosyl bonds | 9 | 187 | 3571 | 144935 | 0.043 |
| GO:0016787 | F | hydrolase activity | 36 | 187 | 21923 | 144935 | 0.074 |
| GO:0016301 | F | kinase activity | 27 | 187 | 15659 | 144935 | 0.073 |
| GO:0017171 | F | serine hydrolase activity | 5 | 187 | 1767 | 144935 | 0.08 |
| GO:0008236 | F | serine-type peptidase activity | 5 | 187 | 1767 | 144935 | 0.08 |
| GO:0004175 | F | endopeptidase activity | 5 | 187 | 2097 | 144935 | 0.14 |
| GO:0016772 | F | transferase activity, transferring phosphorus-containing groups | 30 | 187 | 19025 | 144935 | 0.14 |
| GO:0005515 | F | protein binding | 33 | 187 | 21247 | 144935 | 0.15 |
| GO:0000166 | F | nucleotide binding | 35 | 187 | 29819 | 144935 | 0.76 |
| GO:0008270 | F | zinc ion binding | 10 | 187 | 5778 | 144935 | 0.22 |
| GO:0005488 | F | binding | 100 | 187 | 78438 | 144935 | 0.6 |
| GO:0017076 | F | purine nucleotide binding | 32 | 187 | 28306 | 144935 | 0.82 |
| GO:0016491 | F | oxidoreductase activity | 16 | 187 | 12358 | 144935 | 0.53 |
| GO:0005524 | F | ATP binding | 25 | 187 | 21555 | 144935 | 0.75 |
| GO:0003824 | F | catalytic activity | 101 | 187 | 73079 | 144935 | 0.18 |
| GO:0032559 | F | adenyl ribonucleotide binding | 29 | 187 | 24855 | 144935 | 0.75 |
| GO:0032555 | F | purine ribonucleotide binding | 30 | 187 | 27034 | 144935 | 0.84 |
| GO:0032553 | F | ribonucleotide binding | 30 | 187 | 27034 | 144935 | 0.84 |
| GO:0016705 | F | oxidoreductase activity | 6 | 187 | 3282 | 144935 | 0.25 |
| GO:0030554 | F | adenyl nucleotide binding | 31 | 187 | 26095 | 144935 | 0.72 |
| GO:0008233 | F | peptidase activity | 6 | 187 | 4270 | 144935 | 0.47 |
| GO:0001883 | F | purine nucleoside binding | 31 | 187 | 26095 | 144935 | 0.72 |
| GO:0001882 | F | nucleoside binding | 31 | 187 | 26393 | 144935 | 0.75 |
| GO:0070011 | F | peptidase activity, acting on L-amino acid peptides | 6 | 187 | 4114 | 144935 | 0.44 |
| GO:0030312 | C | external encapsulating structure | 5 | 187 | 288 | 144935 | 4.30E-05 |
| GO:0005618 | C | cell wall | 5 | 187 | 288 | 144935 | 4.30E-05 |
| GO:0016021 | C | integral to membrane | 12 | 187 | 7655 | 144935 | 0.29 |
| GO:0016020 | C | membrane | 24 | 187 | 19834 | 144935 | 0.66 |
| GO:0043234 | C | protein complex | 6 | 187 | 8713 | 144935 | 0.97 |
| GO:0043232 | C | intracellular non-membrane-bounded organelle | 5 | 187 | 6514 | 144935 | 0.93 |
| GO:0044424 | C | intracellular part | 13 | 187 | 20029 | 144935 | 1 |
| GO:0044425 | C | membrane part | 14 | 187 | 10360 | 144935 | 0.47 |
| GO:0043229 | C | intracellular organelle | 9 | 187 | 15014 | 144935 | 1 |
| GO:0043228 | C | non-membrane-bounded organelle | 5 | 187 | 6514 | 144935 | 0.93 |
| GO:0005622 | C | intracellular | 13 | 187 | 20824 | 144935 | 1 |
| GO:0005856 | C | cytoskeleton | 5 | 187 | 2452 | 144935 | 0.21 |
| GO:0031224 | C | intrinsic to membrane | 12 | 187 | 7781 | 144935 | 0.3 |
| GO:0032991 | C | macromolecular complex | 6 | 187 | 12676 | 144935 | 1 |
| GO:0044464 | C | cell part | 41 | 187 | 37668 | 144935 | 0.91 |
| GO:0005623 | C | cell | 41 | 187 | 37668 | 144935 | 0.91 |
| GO:0043226 | C | organelle | 9 | 187 | 15014 | 144935 | 1 |

*Query item denotes the number of genes in input list for a GO Accession number; query total denotes the total number of gene in our query list; BG item denotes total number of genes in background or reference for a GO Accession number; and BG total represents total number of genes in background or reference. P = biological process; F = molecular function; C = cellular components and Term = predicted function/process associated with a particular Accession number.

**Supplementary Table 8.** Enrichment of GO term that represents down-regulated genes common to 12 h and 48 h upon *Fusarium* inoculations.

| **GO accession** | **term type** | **Term** | **query item** | **query total** | **bgitem** | **bgtotal** | **pvalue** |
| --- | --- | --- | --- | --- | --- | --- | --- |
| GO:0006468 | P | protein amino acid phosphorylation | 39 | 138 | 13679 | 144935 | 2.90E-10 |
| GO:0043687 | P | post-translational protein modification | 39 | 138 | 14897 | 144935 | 3.30E-09 |
| GO:0016310 | P | phosphorylation | 39 | 138 | 15144 | 144935 | 5.20E-09 |
| GO:0006796 | P | phosphate metabolic process | 39 | 138 | 15620 | 144935 | 1.20E-08 |
| GO:0006793 | P | phosphorus metabolic process | 39 | 138 | 15620 | 144935 | 1.20E-08 |
| GO:0006464 | P | protein modification process | 39 | 138 | 16098 | 144935 | 2.80E-08 |
| GO:0043412 | P | macromolecule modification | 39 | 138 | 17042 | 144935 | 1.30E-07 |
| GO:0044267 | P | cellular protein metabolic process | 41 | 138 | 23103 | 144935 | 3.70E-05 |
| GO:0019538 | P | protein metabolic process | 44 | 138 | 26987 | 144935 | 0.00013 |
| GO:0010468 | P | regulation of gene expression | 16 | 138 | 5958 | 144935 | 0.00019 |
| GO:0031326 | P | regulation of cellular biosynthetic process | 15 | 138 | 5560 | 144935 | 0.00029 |
| GO:0060255 | P | regulation of macromolecule metabolic process | 16 | 138 | 6139 | 144935 | 0.00027 |
| GO:0009889 | P | regulation of biosynthetic process | 15 | 138 | 5560 | 144935 | 0.00029 |
| GO:0010556 | P | regulation of macromolecule biosynthetic process | 15 | 138 | 5560 | 144935 | 0.00029 |
| GO:0044260 | P | cellular macromolecule metabolic process | 55 | 138 | 38134 | 144935 | 0.00036 |
| GO:0080090 | P | regulation of primary metabolic process | 15 | 138 | 5734 | 144935 | 0.0004 |
| GO:0019222 | P | regulation of metabolic process | 16 | 138 | 6551 | 144935 | 0.00054 |
| GO:0043170 | P | macromolecule metabolic process | 59 | 138 | 42476 | 144935 | 0.00054 |
| GO:0031323 | P | regulation of cellular metabolic process | 15 | 138 | 6059 | 144935 | 0.00071 |
| GO:0051252 | P | regulation of RNA metabolic process | 14 | 138 | 5448 | 144935 | 0.00075 |
| GO:0006355 | P | regulation of transcription, DNA-dependent | 14 | 138 | 5448 | 144935 | 0.00075 |
| GO:0045449 | P | regulation of transcription | 14 | 138 | 5458 | 144935 | 0.00077 |
| GO:0019219 | P | regulation of nucleobase, nucleoside, nucleotide and nucleic acid metabolic process | 14 | 138 | 5568 | 144935 | 0.00093 |
| GO:0051171 | P | regulation of nitrogen compound metabolic process | 14 | 138 | 5568 | 144935 | 0.00093 |
| GO:0008152 | P | metabolic process | 86 | 138 | 73359 | 144935 | 0.0037 |
| GO:0032774 | P | RNA biosynthetic process | 14 | 138 | 7176 | 144935 | 0.0088 |
| GO:0006350 | P | transcription | 14 | 138 | 7152 | 144935 | 0.0086 |
| GO:0006351 | P | transcription, DNA-dependent | 14 | 138 | 7146 | 144935 | 0.0085 |
| GO:0065007 | P | biological regulation | 17 | 138 | 10277 | 144935 | 0.019 |
| GO:0050789 | P | regulation of biological process | 16 | 138 | 9518 | 144935 | 0.019 |
| GO:0050794 | P | regulation of cellular process | 15 | 138 | 8997 | 144935 | 0.025 |
| GO:0044238 | P | primary metabolic process | 62 | 138 | 53794 | 144935 | 0.036 |
| GO:0044237 | P | cellular metabolic process | 57 | 138 | 49113 | 144935 | 0.042 |
| GO:0042221 | P | response to chemical stimulus | 5 | 138 | 2032 | 144935 | 0.046 |
| GO:0006950 | P | response to stress | 7 | 138 | 4184 | 144935 | 0.11 |
| GO:0050896 | P | response to stimulus | 8 | 138 | 5156 | 144935 | 0.12 |
| GO:0055114 | P | oxidation reduction | 15 | 138 | 11488 | 144935 | 0.13 |
| GO:0016070 | P | RNA metabolic process | 14 | 138 | 10967 | 144935 | 0.16 |
| GO:0044249 | P | cellular biosynthetic process | 15 | 138 | 18890 | 144935 | 0.81 |
| GO:0006807 | P | nitrogen compound metabolic process | 14 | 138 | 18913 | 144935 | 0.88 |
| GO:0023052 | P | signaling | 5 | 138 | 3274 | 144935 | 0.2 |
| GO:0034645 | P | cellular macromolecule biosynthetic process | 15 | 138 | 14577 | 144935 | 0.42 |
| GO:0010467 | P | gene expression | 16 | 138 | 14438 | 144935 | 0.3 |
| GO:0006139 | P | nucleobase, nucleoside, nucleotide and nucleic acid metabolic process | 14 | 138 | 16571 | 144935 | 0.72 |
| GO:0009987 | P | cellular process | 62 | 138 | 64217 | 144935 | 0.47 |
| GO:0009058 | P | biosynthetic process | 15 | 138 | 20626 | 144935 | 0.9 |
| GO:0009059 | P | macromolecule biosynthetic process | 15 | 138 | 14608 | 144935 | 0.42 |
| GO:0004672 | F | protein kinase activity | 39 | 138 | 13823 | 144935 | 3.90E-10 |
| GO:0016301 | F | kinase activity | 39 | 138 | 15659 | 144935 | 1.30E-08 |
| GO:0016773 | F | phosphotransferase activity, alcohol group as acceptor | 39 | 138 | 15564 | 144935 | 1.10E-08 |
| GO:0030528 | F | transcription regulator activity | 16 | 138 | 3116 | 144935 | 6.00E-08 |
| GO:0003700 | F | transcription factor activity | 14 | 138 | 2732 | 144935 | 4.30E-07 |
| GO:0016772 | F | transferase activity, transferring phosphorus-containing groups | 39 | 138 | 19025 | 144935 | 2.10E-06 |
| GO:0016740 | F | transferase activity | 45 | 138 | 28658 | 144935 | 0.00026 |
| GO:0020037 | F | heme binding | 11 | 138 | 3260 | 144935 | 0.00032 |
| GO:0046906 | F | tetrapyrrole binding | 11 | 138 | 3528 | 144935 | 0.00061 |
| GO:0005506 | F | iron ion binding | 11 | 138 | 3588 | 144935 | 0.0007 |
| GO:0005488 | F | binding | 93 | 138 | 78438 | 144935 | 0.001 |
| GO:0016835 | F | carbon-oxygen lyase activity | 5 | 138 | 810 | 144935 | 0.0012 |
| GO:0030246 | F | carbohydrate binding | 5 | 138 | 917 | 144935 | 0.002 |
| GO:0005509 | F | calcium ion binding | 6 | 138 | 1474 | 144935 | 0.0031 |
| GO:0043167 | F | ion binding | 24 | 138 | 15344 | 144935 | 0.01 |
| GO:0043169 | F | cation binding | 24 | 138 | 15343 | 144935 | 0.01 |
| GO:0046872 | F | metal ion binding | 24 | 138 | 15190 | 144935 | 0.0092 |
| GO:0043565 | F | sequence-specific DNA binding | 6 | 138 | 1851 | 144935 | 0.009 |
| GO:0016705 | F | oxidoreductase activity, acting on paired donors, with incorporation or reduction of molecular oxygen | 8 | 138 | 3282 | 144935 | 0.014 |
| GO:0032559 | F | adenyl ribonucleotide binding | 34 | 138 | 24855 | 144935 | 0.016 |
| GO:0001883 | F | purine nucleoside binding | 35 | 138 | 26095 | 144935 | 0.019 |
| GO:0030554 | F | adenyl nucleotide binding | 35 | 138 | 26095 | 144935 | 0.019 |
| GO:0001882 | F | nucleoside binding | 35 | 138 | 26393 | 144935 | 0.023 |
| GO:0005524 | F | ATP binding | 29 | 138 | 21555 | 144935 | 0.032 |
| GO:0000166 | F | nucleotide binding | 37 | 138 | 29819 | 144935 | 0.047 |
| GO:0003824 | F | catalytic activity | 80 | 138 | 73079 | 144935 | 0.046 |
| GO:0032555 | F | purine ribonucleotide binding | 34 | 138 | 27034 | 144935 | 0.049 |
| GO:0032553 | F | ribonucleotide binding | 34 | 138 | 27034 | 144935 | 0.049 |
| GO:0017076 | F | purine nucleotide binding | 35 | 138 | 28306 | 144935 | 0.056 |
| GO:0005515 | F | protein binding | 27 | 138 | 21247 | 144935 | 0.07 |
| GO:0003677 | F | DNA binding | 14 | 138 | 9698 | 144935 | 0.079 |
| GO:0016829 | F | lyase activity | 5 | 138 | 2375 | 144935 | 0.078 |
| GO:0016491 | F | oxidoreductase activity | 16 | 138 | 12358 | 144935 | 0.13 |
| GO:0046914 | F | transition metal ion binding | 13 | 138 | 10265 | 144935 | 0.18 |
| GO:0016798 | F | hydrolase activity, acting on glycosyl bonds | 5 | 138 | 3571 | 144935 | 0.25 |
| GO:0043531 | F | ADP binding | 5 | 138 | 3362 | 144935 | 0.22 |
| GO:0003676 | F | nucleic acid binding | 14 | 138 | 18400 | 144935 | 0.85 |
| GO:0016787 | F | hydrolase activity | 11 | 138 | 21923 | 144935 | 1 |
| GO:0016020 | C | membrane | 11 | 138 | 19834 | 144935 | 0.99 |
| GO:0044464 | C | cell part | 13 | 138 | 37668 | 144935 | 1 |
| GO:0005623 | C | cell | 13 | 138 | 37668 | 144935 | 1 |

*Query item denotes the number of genes in input list for a GO Accession number; query total denotes the total number of gene in our query list; BG item denotes total number of genes in background or reference for a GO Accession number; and BG total represents total number of genes in background or reference. P = biological process; F = molecular function; C = cellular components and Term = predicted function/process associated with a particular Accession number.

**Supplementary Table 9.** Enrichment of GO terms that represents up-regulated genes unique to 12 h upon *Fusarium* inoculations.

| GO term | Term type | Term | Number in input list | Number in BG/Ref | p-value |
| --- | --- | --- | --- | --- | --- |
| GO:0071555 | P | cell wall organization | 6 | 386 | 4.00E-05 |
| GO:0006979 | P | response to oxidative stress | 8 | 1064 | 0.00032 |
| GO:0071554 | P | cell wall organization or biogenesis | 6 | 660 | 0.00069 |
| GO:0065009 | P | regulation of molecular function | 7 | 1155 | 0.0025 |
| GO:0050790 | P | regulation of catalytic activity | 7 | 1145 | 0.0024 |
| GO:0016310 | P | phosphorylation | 37 | 15144 | 0.0044 |
| GO:0006073 | P | cellular glucan metabolic process | 5 | 746 | 0.0069 |
| GO:0006468 | P | protein amino acid phosphorylation | 34 | 13679 | 0.005 |
| GO:0043687 | P | post-translational protein modification | 37 | 14897 | 0.0034 |
| GO:0044042 | P | glucan metabolic process | 5 | 746 | 0.0069 |
| GO:0005975 | P | carbohydrate metabolic process | 21 | 7421 | 0.0069 |
| GO:0006796 | P | phosphate metabolic process | 37 | 15620 | 0.0072 |
| GO:0006793 | P | phosphorus metabolic process | 37 | 15620 | 0.0072 |
| GO:0005976 | P | polysaccharide metabolic process | 6 | 1107 | 0.0086 |
| GO:0044264 | P | cellular polysaccharide metabolic process | 5 | 854 | 0.012 |
| GO:0006464 | P | protein modification process | 37 | 16098 | 0.011 |
| GO:0042221 | P | response to chemical stimulus | 8 | 2032 | 0.016 |
| GO:0006950 | P | response to stress | 13 | 4184 | 0.016 |
| GO:0043412 | P | macromolecule modification | 37 | 17042 | 0.026 |
| GO:0050896 | P | response to stimulus | 13 | 5156 | 0.064 |
| GO:0006810 | P | transport | 16 | 12862 | 0.86 |
| GO:0044249 | P | cellular biosynthetic process | 8 | 18890 | 1 |
| GO:0006807 | P | nitrogen compound metabolic process | 6 | 18913 | 1 |
| GO:0023052 | P | signaling | 6 | 3274 | 0.41 |
| GO:0034645 | P | cellular macromolecule biosynthetic process | 5 | 14577 | 1 |
| GO:0050789 | P | regulation of biological process | 8 | 9518 | 0.98 |
| GO:0044267 | P | cellular protein metabolic process | 39 | 23103 | 0.33 |
| GO:0044262 | P | cellular carbohydrate metabolic process | 8 | 3762 | 0.24 |
| GO:0044260 | P | cellular macromolecule metabolic process | 48 | 38134 | 0.97 |
| GO:0016043 | P | cellular component organization | 6 | 4641 | 0.74 |
| GO:0051179 | P | localization | 16 | 12991 | 0.87 |
| GO:0065007 | P | biological regulation | 12 | 10277 | 0.89 |
| GO:0006508 | P | proteolysis | 8 | 4923 | 0.51 |
| GO:0006811 | P | ion transport | 6 | 3014 | 0.33 |
| GO:0006996 | P | organelle organization | 5 | 3231 | 0.57 |
| GO:0009987 | P | cellular process | 86 | 64217 | 0.98 |
| GO:0006629 | P | lipid metabolic process | 8 | 4142 | 0.32 |
| GO:0006812 | P | cation transport | 6 | 2427 | 0.18 |
| GO:0050794 | P | regulation of cellular process | 8 | 8997 | 0.97 |
| GO:0009058 | P | biosynthetic process | 11 | 20626 | 1 |
| GO:0009059 | P | macromolecule biosynthetic process | 5 | 14608 | 1 |
| GO:0008152 | P | metabolic process | 114 | 73359 | 0.57 |
| GO:0051234 | P | establishment of localization | 16 | 12862 | 0.86 |
| GO:0055114 | P | oxidation reduction | 22 | 11488 | 0.19 |
| GO:0044238 | P | primary metabolic process | 80 | 53794 | 0.74 |
| GO:0055085 | P | transmembrane transport | 9 | 6293 | 0.66 |
| GO:0019538 | P | protein metabolic process | 46 | 26987 | 0.29 |
| GO:0044237 | P | cellular metabolic process | 65 | 49113 | 0.96 |
| GO:0043170 | P | macromolecule metabolic process | 56 | 42476 | 0.95 |
| GO:0030599 | F | pectinesterase activity | 8 | 269 | 1.70E-08 |
| GO:0004857 | F | enzyme inhibitor activity | 8 | 345 | 1.10E-07 |
| GO:0030234 | F | enzyme regulator activity | 12 | 1452 | 4.20E-06 |
| GO:0005509 | F | calcium ion binding | 11 | 1474 | 2.70E-05 |
| GO:0004091 | F | carboxylesterase activity | 8 | 794 | 4.50E-05 |
| GO:0004553 | F | hydrolase activity, hydrolyzing O-glycosyl compounds | 16 | 3355 | 9.60E-05 |
| GO:0016798 | F | hydrolase activity, acting on glycosyl bonds | 16 | 3571 | 0.00019 |
| GO:0016684 | F | oxidoreductase activity, acting on peroxide as acceptor | 8 | 1097 | 0.00039 |
| GO:0004601 | F | peroxidase activity | 8 | 1097 | 0.00039 |
| GO:0016209 | F | antioxidant activity | 8 | 1149 | 0.00053 |
| GO:0046872 | F | metal ion binding | 39 | 15190 | 0.0014 |
| GO:0043169 | F | cation binding | 39 | 15343 | 0.0017 |
| GO:0043167 | F | ion binding | 39 | 15344 | 0.0017 |
| GO:0004672 | F | protein kinase activity | 34 | 13823 | 0.0058 |
| GO:0016301 | F | kinase activity | 37 | 15659 | 0.0075 |
| GO:0003824 | F | catalytic activity | 133 | 73079 | 0.0082 |
| GO:0016773 | F | phosphotransferase activity, alcohol group as acceptor | 36 | 15564 | 0.011 |
| GO:0005506 | F | iron ion binding | 12 | 3588 | 0.012 |
| GO:0020037 | F | heme binding | 11 | 3260 | 0.015 |
| GO:0009055 | F | electron carrier activity | 5 | 955 | 0.018 |
| GO:0016788 | F | hydrolase activity, acting on ester bonds | 12 | 3945 | 0.023 |
| GO:0046906 | F | tetrapyrrole binding | 11 | 3528 | 0.025 |
| GO:0050662 | F | coenzyme binding | 8 | 2355 | 0.034 |
| GO:0016740 | F | transferase activity | 56 | 28658 | 0.041 |
| GO:0016746 | F | transferase activity, transferring acyl groups | 7 | 2002 | 0.04 |
| GO:0048037 | F | cofactor binding | 10 | 3366 | 0.041 |
| GO:0046914 | F | transition metal ion binding | 23 | 10265 | 0.054 |
| GO:0016772 | F | transferase activity, transferring phosphorus-containing groups | 38 | 19025 | 0.069 |
| GO:0016747 | F | transferase activity, transferring acyl groups other than amino-acyl groups | 5 | 1445 | 0.079 |
| GO:0016787 | F | hydrolase activity | 42 | 21923 | 0.095 |
| GO:0016491 | F | oxidoreductase activity | 25 | 12358 | 0.11 |
| GO:0008270 | F | zinc ion binding | 7 | 5778 | 0.8 |
| GO:0000166 | F | nucleotide binding | 43 | 29819 | 0.75 |
| GO:0016757 | F | transferase activity, transferring glycosyl groups | 7 | 4235 | 0.5 |
| GO:0016758 | F | transferase activity, transferring hexosyl groups | 7 | 3493 | 0.31 |
| GO:0005488 | F | binding | 119 | 78438 | 0.72 |
| GO:0003676 | F | nucleic acid binding | 8 | 18400 | 1 |
| GO:0003677 | F | DNA binding | 5 | 9698 | 1 |
| GO:0017076 | F | purine nucleotide binding | 40 | 28306 | 0.79 |
| GO:0005524 | F | ATP binding | 32 | 21555 | 0.66 |
| GO:0022892 | F | substrate-specific transporter activity | 5 | 4138 | 0.78 |
| GO:0015075 | F | ion transmembrane transporter activity | 5 | 3323 | 0.6 |
| GO:0032559 | F | adenyl ribonucleotide binding | 33 | 24855 | 0.87 |
| GO:0032555 | F | purine ribonucleotide binding | 35 | 27034 | 0.91 |
| GO:0032553 | F | ribonucleotide binding | 35 | 27034 | 0.91 |
| GO:0016705 | F | oxidoreductase activity, acting on paired donors, with incorporation or reduction of molecular oxygen | 7 | 3282 | 0.26 |
| GO:0022891 | F | substrate-specific transmembrane transporter activity | 5 | 3541 | 0.65 |
| GO:0005215 | F | transporter activity | 8 | 8389 | 0.95 |
| GO:0008324 | F | cation transmembrane transporter activity | 5 | 2580 | 0.38 |
| GO:0030554 | F | adenyl nucleotide binding | 38 | 26095 | 0.72 |
| GO:0005515 | F | protein binding | 39 | 21247 | 0.16 |
| GO:0008233 | F | peptidase activity | 9 | 4270 | 0.23 |
| GO:0004175 | F | endopeptidase activity | 5 | 2097 | 0.23 |
| GO:0001883 | F | purine nucleoside binding | 38 | 26095 | 0.72 |
| GO:0001882 | F | nucleoside binding | 39 | 26393 | 0.68 |
| GO:0022857 | F | transmembrane transporter activity | 7 | 6212 | 0.86 |
| GO:0070011 | F | peptidase activity, acting on L-amino acid peptides | 9 | 4114 | 0.2 |
| GO:0030312 | C | external encapsulating structure | 7 | 288 | 5.10E-07 |
| GO:0005618 | C | cell wall | 7 | 288 | 5.10E-07 |
| GO:0016021 | C | integral to membrane | 10 | 7655 | 0.76 |
| GO:0016020 | C | membrane | 25 | 19834 | 0.9 |
| GO:0043234 | C | protein complex | 8 | 8713 | 0.97 |
| GO:0044424 | C | intracellular part | 13 | 20029 | 1 |
| GO:0044422 | C | organelle part | 5 | 7005 | 0.99 |
| GO:0043229 | C | intracellular organelle | 8 | 15014 | 1 |
| GO:0005622 | C | intracellular | 13 | 20824 | 1 |
| GO:0044425 | C | membrane part | 13 | 10360 | 0.83 |
| GO:0031224 | C | intrinsic to membrane | 11 | 7781 | 0.68 |
| GO:0032991 | C | macromolecular complex | 10 | 12676 | 1 |
| GO:0044464 | C | cell part | 44 | 37668 | 0.99 |
| GO:0005623 | C | cell | 44 | 37668 | 0.99 |
| GO:0043226 | C | organelle | 8 | 15014 | 1 |
| GO:0044446 | C | intracellular organelle part | 5 | 7005 | 0.99 |

*BG numbers represents number of genes in background or reference. P = biological process; F = molecular function; C = cellular components and Term = predicted function/process associated with a particular Accession number.

**Supplementary Table 10.** Enrichment of GO terms that represents down-regulated genes unique to 12 h after *Fusarium* inoculations.

| GO term | Term type | Term | Number in input list | Number in BG/Ref | p-value |
| --- | --- | --- | --- | --- | --- |
| GO:0000160 | P | two-component signal transduction system (phosphorelay) | 15 | 272 | 7.90E-20 |
| GO:0023046 | P | signaling process | 16 | 2390 | 1.30E-07 |
| GO:0023060 | P | signal transmission | 16 | 2390 | 1.30E-07 |
| GO:0023052 | P | signaling | 17 | 3274 | 1.70E-06 |
| GO:0006508 | P | proteolysis | 13 | 4923 | 0.013 |
| GO:0032774 | P | RNA biosynthetic process | 12 | 7176 | 0.22 |
| GO:0019222 | P | regulation of metabolic process | 13 | 6551 | 0.086 |
| GO:0016310 | P | phosphorylation | 11 | 15144 | 0.99 |
| GO:0031323 | P | regulation of cellular metabolic process | 12 | 6059 | 0.098 |
| GO:0044249 | P | cellular biosynthetic process | 14 | 18890 | 0.99 |
| GO:0006807 | P | nitrogen compound metabolic process | 15 | 18913 | 0.99 |
| GO:0045449 | P | regulation of transcription | 12 | 5458 | 0.054 |
| GO:0034645 | P | cellular macromolecule biosynthetic process | 13 | 14577 | 0.95 |
| GO:0050789 | P | regulation of biological process | 14 | 9518 | 0.35 |
| GO:0080090 | P | regulation of primary metabolic process | 12 | 5734 | 0.072 |
| GO:0044267 | P | cellular protein metabolic process | 15 | 23103 | 1 |
| GO:0044260 | P | cellular macromolecule metabolic process | 29 | 38134 | 1 |
| GO:0060255 | P | regulation of macromolecule metabolic process | 13 | 6139 | 0.058 |
| GO:0010467 | P | gene expression | 14 | 14438 | 0.9 |
| GO:0065007 | P | biological regulation | 16 | 10277 | 0.26 |
| GO:0031326 | P | regulation of cellular biosynthetic process | 12 | 5560 | 0.06 |
| GO:0010468 | P | regulation of gene expression | 13 | 5958 | 0.048 |
| GO:0006468 | P | protein amino acid phosphorylation | 11 | 13679 | 0.97 |
| GO:0009987 | P | cellular process | 43 | 64217 | 1 |
| GO:0006629 | P | lipid metabolic process | 7 | 4142 | 0.29 |
| GO:0006139 | P | nucleobase, nucleoside, nucleotide and nucleic acid metabolic process | 14 | 16571 | 0.97 |
| GO:0019219 | P | regulation of nucleobase, nucleoside, nucleotide and nucleic acid metabolic process | 12 | 5568 | 0.06 |
| GO:0043687 | P | post-translational protein modification | 11 | 14897 | 0.99 |
| GO:0006810 | P | transport | 5 | 12862 | 1 |
| GO:0009889 | P | regulation of biosynthetic process | 12 | 5560 | 0.06 |
| GO:0006464 | P | protein modification process | 11 | 16098 | 1 |
| GO:0050794 | P | regulation of cellular process | 13 | 8997 | 0.39 |
| GO:0009058 | P | biosynthetic process | 15 | 20626 | 1 |
| GO:0043412 | P | macromolecule modification | 11 | 17042 | 1 |
| GO:0051171 | P | regulation of nitrogen compound metabolic process | 12 | 5568 | 0.06 |
| GO:0008152 | P | metabolic process | 89 | 73359 | 0.83 |
| GO:0051234 | P | establishment of localization | 5 | 12862 | 1 |
| GO:0009059 | P | macromolecule biosynthetic process | 13 | 14608 | 0.95 |
| GO:0051179 | P | localization | 5 | 12991 | 1 |
| GO:0055114 | P | oxidation reduction | 21 | 11488 | 0.071 |
| GO:0016070 | P | RNA metabolic process | 13 | 10967 | 0.67 |
| GO:0044238 | P | primary metabolic process | 59 | 53794 | 0.96 |
| GO:0005975 | P | carbohydrate metabolic process | 13 | 7421 | 0.17 |
| GO:0019538 | P | protein metabolic process | 24 | 26987 | 0.99 |
| GO:0050896 | P | response to stimulus | 7 | 5156 | 0.5 |
| GO:0051252 | P | regulation of RNA metabolic process | 12 | 5448 | 0.053 |
| GO:0006950 | P | response to stress | 6 | 4184 | 0.46 |
| GO:0044237 | P | cellular metabolic process | 35 | 49113 | 1 |
| GO:0043170 | P | macromolecule metabolic process | 39 | 42476 | 1 |
| GO:0006355 | P | regulation of transcription, DNA-dependent | 12 | 5448 | 0.053 |
| GO:0006796 | P | phosphate metabolic process | 11 | 15620 | 0.99 |
| GO:0010556 | P | regulation of macromolecule biosynthetic process | 12 | 5560 | 0.06 |
| GO:0006350 | P | transcription | 12 | 7152 | 0.22 |
| GO:0006351 | P | transcription, DNA-dependent | 12 | 7146 | 0.22 |
| GO:0006793 | P | phosphorus metabolic process | 11 | 15620 | 0.99 |
| GO:0060089 | F | molecular transducer activity | 16 | 789 | 1.70E-14 |
| GO:0004871 | F | signal transducer activity | 16 | 789 | 1.70E-14 |
| GO:0004857 | F | enzyme inhibitor activity | 10 | 345 | 5.50E-11 |
| GO:0000156 | F | two-component response regulator activity | 8 | 206 | 5.20E-10 |
| GO:0030599 | F | pectinesterase activity | 8 | 269 | 3.90E-09 |
| GO:0030528 | F | transcription regulator activity | 19 | 3116 | 3.50E-08 |
| GO:0004091 | F | carboxylesterase activity | 8 | 794 | 1.20E-05 |
| GO:0030234 | F | enzyme regulator activity | 10 | 1452 | 2.50E-05 |
| GO:0003682 | F | chromatin binding | 10 | 1546 | 4.20E-05 |
| GO:0016705 | F | oxidoreductase activity, acting on paired donors, with incorporation or reduction of molecular oxygen | 14 | 3282 | 0.00011 |
| GO:0043565 | F | sequence-specific DNA binding | 10 | 1851 | 0.00018 |
| GO:0016788 | F | hydrolase activity, acting on ester bonds | 14 | 3945 | 0.00072 |
| GO:0003700 | F | transcription factor activity | 11 | 2732 | 0.00099 |
| GO:0020037 | F | heme binding | 11 | 3260 | 0.0038 |
| GO:0046906 | F | tetrapyrrole binding | 11 | 3528 | 0.0068 |
| GO:0005506 | F | iron ion binding | 11 | 3588 | 0.0077 |
| GO:0004553 | F | hydrolase activity, hydrolyzing O-glycosyl compounds | 10 | 3355 | 0.013 |
| GO:0003677 | F | DNA binding | 21 | 9698 | 0.015 |
| GO:0016798 | F | hydrolase activity, acting on glycosyl bonds | 10 | 3571 | 0.019 |
| GO:0016747 | F | transferase activity, transferring acyl groups other than amino-acyl groups | 5 | 1445 | 0.041 |
| GO:0046983 | F | protein dimerization activity | 5 | 1780 | 0.084 |
| GO:0016491 | F | oxidoreductase activity | 22 | 12358 | 0.081 |
| GO:0070011 | F | peptidase activity, acting on L-amino acid peptides | 9 | 4114 | 0.089 |
| GO:0043169 | F | cation binding | 23 | 15343 | 0.26 |
| GO:0000166 | F | nucleotide binding | 19 | 29819 | 1 |
| GO:0016740 | F | transferase activity | 30 | 28658 | 0.92 |
| GO:0016746 | F | transferase activity, transferring acyl groups | 5 | 2002 | 0.12 |
| GO:0046872 | F | metal ion binding | 23 | 15190 | 0.25 |
| GO:0043531 | F | ADP binding | 5 | 3362 | 0.44 |
| GO:0016757 | F | transferase activity, transferring glycosyl groups | 6 | 4235 | 0.47 |
| GO:0016758 | F | transferase activity, transferring hexosyl groups | 5 | 3493 | 0.48 |
| GO:0005488 | F | binding | 98 | 78438 | 0.73 |
| GO:0003676 | F | nucleic acid binding | 24 | 18400 | 0.52 |
| GO:0017076 | F | purine nucleotide binding | 17 | 28306 | 1 |
| GO:0005524 | F | ATP binding | 8 | 21555 | 1 |
| GO:0016787 | F | hydrolase activity | 35 | 21923 | 0.11 |
| GO:0016301 | F | kinase activity | 12 | 15659 | 0.99 |
| GO:0003824 | F | catalytic activity | 92 | 73079 | 0.68 |
| GO:0016773 | F | phosphotransferase activity, alcohol group as acceptor | 13 | 15564 | 0.97 |
| GO:0016772 | F | transferase activity, transferring phosphorus-containing groups | 14 | 19025 | 1 |
| GO:0032559 | F | adenyl ribonucleotide binding | 13 | 24855 | 1 |
| GO:0032555 | F | purine ribonucleotide binding | 13 | 27034 | 1 |
| GO:0032553 | F | ribonucleotide binding | 13 | 27034 | 1 |
| GO:0043167 | F | ion binding | 23 | 15344 | 0.26 |
| GO:0048037 | F | cofactor binding | 5 | 3366 | 0.44 |
| GO:0030554 | F | adenyl nucleotide binding | 17 | 26095 | 1 |
| GO:0005515 | F | protein binding | 28 | 21247 | 0.5 |
| GO:0008233 | F | peptidase activity | 9 | 4270 | 0.11 |
| GO:0004175 | F | endopeptidase activity | 5 | 2097 | 0.14 |
| GO:0001883 | F | purine nucleoside binding | 17 | 26095 | 1 |
| GO:0001882 | F | nucleoside binding | 17 | 26393 | 1 |
| GO:0050662 | F | coenzyme binding | 5 | 2355 | 0.19 |
| GO:0004672 | F | protein kinase activity | 11 | 13823 | 0.97 |
| GO:0046914 | F | transition metal ion binding | 18 | 10265 | 0.12 |
| GO:0016020 | C | membrane | 13 | 19834 | 1 |
| GO:0043231 | C | intracellular membrane-bounded organelle | 6 | 8850 | 0.98 |
| GO:0044424 | C | intracellular part | 8 | 20029 | 1 |
| GO:0044422 | C | organelle part | 5 | 7005 | 0.95 |
| GO:0043229 | C | intracellular organelle | 8 | 15014 | 1 |
| GO:0005622 | C | intracellular | 10 | 20824 | 1 |
| GO:0043227 | C | membrane-bounded organelle | 8 | 8922 | 0.9 |
| GO:0044446 | C | intracellular organelle part | 5 | 7005 | 0.95 |
| GO:0005634 | C | nucleus | 6 | 6703 | 0.87 |
| GO:0032991 | C | macromolecular complex | 5 | 12676 | 1 |
| GO:0044464 | C | cell part | 23 | 37668 | 1 |
| GO:0005623 | C | cell | 23 | 37668 | 1 |
| GO:0043226 | C | organelle | 8 | 15014 | 1 |

*BG numbers represents number of genes in background or reference. P = biological process; F = molecular function; C = cellular components and Term = predicted function/process associated with a particular Accession number.

**Supplementary Table 11.** Enrichment of GO terms that represents up-regulated genes unique to 48 h upon *Fusarium* inoculations.

| **GO term** | **Term type** | **Term** | **Number in input list** | **Number in BG/Ref** | **p-value** |
| --- | --- | --- | --- | --- | --- |
| GO:0015979 | P | photosynthesis | 42 | 1157 | 3.50E-23 |
| GO:0009765 | P | photosynthesis, light harvesting | 20 | 216 | 5.40E-19 |
| GO:0019684 | P | photosynthesis, light reaction | 23 | 609 | 1.20E-13 |
| GO:0006091 | P | generation of precursor metabolites and energy | 29 | 2402 | 8.90E-06 |
| GO:0071103 | P | DNA conformation change | 14 | 856 | 9.70E-05 |
| GO:0044092 | P | negative regulation of molecular function | 6 | 189 | 0.00038 |
| GO:0043086 | P | negative regulation of catalytic activity | 6 | 189 | 0.00038 |
| GO:0008152 | P | metabolic process | 393 | 73359 | 0.00077 |
| GO:0006284 | P | base-excision repair | 5 | 155 | 0.0011 |
| GO:0006323 | P | DNA packaging | 10 | 627 | 0.0011 |
| GO:0034728 | P | nucleosome organization | 9 | 541 | 0.0015 |
| GO:0031497 | P | chromatin assembly | 9 | 541 | 0.0015 |
| GO:0006334 | P | nucleosome assembly | 9 | 541 | 0.0015 |
| GO:0006333 | P | chromatin assembly or disassembly | 9 | 554 | 0.0017 |
| GO:0065004 | P | protein-DNA complex assembly | 9 | 597 | 0.0028 |
| GO:0055114 | P | oxidation reduction | 75 | 11488 | 0.0042 |
| GO:0000079 | P | regulation of cyclin-dependent protein kinase activity | 6 | 310 | 0.0043 |
| GO:0006260 | P | DNA replication | 12 | 1137 | 0.01 |
| GO:0065009 | P | regulation of molecular function | 12 | 1155 | 0.011 |
| GO:0050790 | P | regulation of catalytic activity | 12 | 1145 | 0.011 |
| GO:0007049 | P | cell cycle | 12 | 1154 | 0.011 |
| GO:0006325 | P | chromatin organization | 11 | 1039 | 0.013 |
| GO:0006629 | P | lipid metabolic process | 30 | 4142 | 0.018 |
| GO:0022607 | P | cellular component assembly | 17 | 2022 | 0.02 |
| GO:0022402 | P | cell cycle process | 7 | 569 | 0.022 |
| GO:0034622 | P | cellular macromolecular complex assembly | 12 | 1330 | 0.029 |
| GO:0019220 | P | regulation of phosphate metabolic process | 6 | 513 | 0.039 |
| GO:0045859 | P | regulation of protein kinase activity | 6 | 510 | 0.038 |
| GO:0042325 | P | regulation of phosphorylation | 6 | 510 | 0.038 |
| GO:0043549 | P | regulation of kinase activity | 6 | 510 | 0.038 |
| GO:0065003 | P | macromolecular complex assembly | 15 | 1833 | 0.034 |
| GO:0006952 | P | defense response | 5 | 362 | 0.032 |
| GO:0051338 | P | regulation of transferase activity | 6 | 510 | 0.038 |
| GO:0051174 | P | regulation of phosphorus metabolic process | 6 | 513 | 0.039 |
| GO:0051726 | P | regulation of cell cycle | 6 | 518 | 0.041 |
| GO:0006259 | P | DNA metabolic process | 26 | 3762 | 0.043 |
| GO:0080090 | P | regulation of primary metabolic process | 27 | 5734 | 0.56 |
| GO:0019222 | P | regulation of metabolic process | 40 | 6551 | 0.072 |
| GO:0007165 | P | signal transduction | 6 | 2146 | 0.94 |
| GO:0044282 | P | small molecule catabolic process | 7 | 991 | 0.2 |
| GO:0044283 | P | small molecule biosynthetic process | 7 | 2069 | 0.86 |
| GO:0051716 | P | cellular response to stimulus | 8 | 2079 | 0.78 |
| GO:0055085 | P | transmembrane transport | 32 | 6293 | 0.39 |
| GO:0006281 | P | DNA repair | 8 | 2064 | 0.77 |
| GO:0060255 | P | regulation of macromolecule metabolic process | 33 | 6139 | 0.27 |
| GO:0043436 | P | oxoacid metabolic process | 10 | 3777 | 0.99 |
| GO:0046483 | P | heterocycle metabolic process | 7 | 2215 | 0.9 |
| GO:0019538 | P | protein metabolic process | 78 | 26987 | 1 |
| GO:0016052 | P | carbohydrate catabolic process | 6 | 1105 | 0.43 |
| GO:0016053 | P | organic acid biosynthetic process | 6 | 1233 | 0.54 |
| GO:0033554 | P | cellular response to stress | 8 | 2073 | 0.77 |
| GO:0044281 | P | small molecule metabolic process | 25 | 8653 | 1 |
| GO:0019320 | P | hexose catabolic process | 5 | 808 | 0.35 |
| GO:0034645 | P | cellular macromolecule biosynthetic process | 47 | 14577 | 1 |
| GO:0006807 | P | nitrogen compound metabolic process | 76 | 18913 | 0.96 |
| GO:0050789 | P | regulation of biological process | 49 | 9518 | 0.32 |
| GO:0044267 | P | cellular protein metabolic process | 54 | 23103 | 1 |
| GO:0044265 | P | cellular macromolecule catabolic process | 7 | 2278 | 0.92 |
| GO:0044262 | P | cellular carbohydrate metabolic process | 11 | 3762 | 0.97 |
| GO:0044260 | P | cellular macromolecule metabolic process | 121 | 38134 | 1 |
| GO:0016043 | P | cellular component organization | 21 | 4641 | 0.63 |
| GO:0065007 | P | biological regulation | 56 | 10277 | 0.17 |
| GO:0006812 | P | cation transport | 10 | 2427 | 0.72 |
| GO:0006810 | P | transport | 68 | 12862 | 0.21 |
| GO:0009308 | P | amine metabolic process | 7 | 2665 | 0.97 |
| GO:0019318 | P | hexose metabolic process | 5 | 1245 | 0.71 |
| GO:0006811 | P | ion transport | 14 | 3014 | 0.58 |
| GO:0009889 | P | regulation of biosynthetic process | 27 | 5560 | 0.49 |
| GO:0050794 | P | regulation of cellular process | 43 | 8997 | 0.52 |
| GO:0043412 | P | macromolecule modification | 51 | 17042 | 1 |
| GO:0051234 | P | establishment of localization | 68 | 12862 | 0.21 |
| GO:0016070 | P | RNA metabolic process | 35 | 10967 | 1 |
| GO:0044271 | P | cellular nitrogen compound biosynthetic process | 6 | 1441 | 0.69 |
| GO:0046394 | P | carboxylic acid biosynthetic process | 6 | 1233 | 0.54 |
| GO:0050896 | P | response to stimulus | 27 | 5156 | 0.34 |
| GO:0006950 | P | response to stress | 23 | 4184 | 0.28 |
| GO:0006355 | P | regulation of transcription, DNA-dependent | 26 | 5448 | 0.53 |
| GO:0010556 | P | regulation of macromolecule biosynthetic process | 27 | 5560 | 0.49 |
| GO:0006350 | P | transcription | 31 | 7152 | 0.74 |
| GO:0006351 | P | transcription, DNA-dependent | 31 | 7146 | 0.73 |
| GO:0006519 | P | cellular amino acid and derivative metabolic process | 8 | 2606 | 0.93 |
| GO:0032774 | P | RNA biosynthetic process | 31 | 7176 | 0.74 |
| GO:0070271 | P | protein complex biogenesis | 5 | 1122 | 0.62 |
| GO:0016310 | P | phosphorylation | 50 | 15144 | 1 |
| GO:0044248 | P | cellular catabolic process | 12 | 2887 | 0.73 |
| GO:0044249 | P | cellular biosynthetic process | 63 | 18890 | 1 |
| GO:0034641 | P | cellular nitrogen compound metabolic process | 11 | 3316 | 0.92 |
| GO:0023052 | P | signaling | 12 | 3274 | 0.86 |
| GO:0006139 | P | nucleobase, nucleoside, nucleotide and nucleic acid metabolic process | 66 | 16571 | 0.95 |
| GO:0009057 | P | macromolecule catabolic process | 8 | 2625 | 0.93 |
| GO:0043933 | P | macromolecular complex subunit organization | 15 | 2087 | 0.082 |
| GO:0045449 | P | regulation of transcription | 26 | 5458 | 0.54 |
| GO:0006508 | P | proteolysis | 26 | 4923 | 0.33 |
| GO:0044275 | P | cellular carbohydrate catabolic process | 5 | 891 | 0.42 |
| GO:0043687 | P | post-translational protein modification | 46 | 14897 | 1 |
| GO:0009987 | P | cellular process | 271 | 64217 | 1 |
| GO:0006974 | P | response to DNA damage stimulus | 8 | 2067 | 0.77 |
| GO:0044106 | P | cellular amine metabolic process | 7 | 2394 | 0.94 |
| GO:0023046 | P | signaling process | 10 | 2390 | 0.71 |
| GO:0030001 | P | metal ion transport | 8 | 972 | 0.099 |
| GO:0044255 | P | cellular lipid metabolic process | 8 | 2348 | 0.87 |
| GO:0006979 | P | response to oxidative stress | 6 | 1064 | 0.4 |
| GO:0042180 | P | cellular ketone metabolic process | 10 | 3797 | 0.99 |
| GO:0006082 | P | organic acid metabolic process | 10 | 3796 | 0.99 |
| GO:0005996 | P | monosaccharide metabolic process | 5 | 1329 | 0.76 |
| GO:0051252 | P | regulation of RNA metabolic process | 26 | 5448 | 0.53 |
| GO:0043170 | P | macromolecule metabolic process | 149 | 42476 | 1 |
| GO:0034621 | P | cellular macromolecular complex subunit organization | 12 | 1584 | 0.083 |
| GO:0006006 | P | glucose metabolic process | 5 | 968 | 0.49 |
| GO:0006007 | P | glucose catabolic process | 5 | 808 | 0.35 |
| GO:0046365 | P | monosaccharide catabolic process | 5 | 808 | 0.35 |
| GO:0031326 | P | regulation of cellular biosynthetic process | 27 | 5560 | 0.49 |
| GO:0031323 | P | regulation of cellular metabolic process | 34 | 6059 | 0.19 |
| GO:0019752 | P | carboxylic acid metabolic process | 10 | 3777 | 0.99 |
| GO:0022403 | P | cell cycle phase | 6 | 555 | 0.054 |
| GO:0006096 | P | glycolysis | 5 | 668 | 0.22 |
| GO:0006520 | P | cellular amino acid metabolic process | 7 | 2251 | 0.91 |
| GO:0071554 | P | cell wall organization or biogenesis | 5 | 660 | 0.21 |
| GO:0010467 | P | gene expression | 43 | 14438 | 1 |
| GO:0010468 | P | regulation of gene expression | 32 | 5958 | 0.28 |
| GO:0006468 | P | protein amino acid phosphorylation | 44 | 13679 | 1 |
| GO:0046164 | P | alcohol catabolic process | 5 | 891 | 0.42 |
| GO:0000278 | P | mitotic cell cycle | 5 | 437 | 0.062 |
| GO:0019219 | P | regulation of nucleobase, nucleoside, nucleotide and nucleic acid metabolic process | 27 | 5568 | 0.5 |
| GO:0006461 | P | protein complex assembly | 5 | 1122 | 0.62 |
| GO:0006464 | P | protein modification process | 46 | 16098 | 1 |
| GO:0023060 | P | signal transmission | 10 | 2390 | 0.71 |
| GO:0009058 | P | biosynthetic process | 70 | 20626 | 1 |
| GO:0009059 | P | macromolecule biosynthetic process | 47 | 14608 | 1 |
| GO:0051171 | P | regulation of nitrogen compound metabolic process | 27 | 5568 | 0.5 |
| GO:0042221 | P | response to chemical stimulus | 10 | 2032 | 0.51 |
| GO:0009056 | P | catabolic process | 14 | 3313 | 0.71 |
| GO:0051179 | P | localization | 68 | 12991 | 0.23 |
| GO:0008610 | P | lipid biosynthetic process | 7 | 1647 | 0.67 |
| GO:0006996 | P | organelle organization | 16 | 3231 | 0.48 |
| GO:0044238 | P | primary metabolic process | 215 | 53794 | 1 |
| GO:0007017 | P | microtubule-based process | 9 | 1727 | 0.44 |
| GO:0005975 | P | carbohydrate metabolic process | 38 | 7421 | 0.36 |
| GO:0051276 | P | chromosome organization | 12 | 1518 | 0.066 |
| GO:0005976 | P | polysaccharide metabolic process | 5 | 1107 | 0.61 |
| GO:0007018 | P | microtubule-based movement | 7 | 981 | 0.19 |
| GO:0044237 | P | cellular metabolic process | 202 | 49113 | 1 |
| GO:0006066 | P | alcohol metabolic process | 6 | 1800 | 0.86 |
| GO:0006796 | P | phosphate metabolic process | 50 | 15620 | 1 |
| GO:0044085 | P | cellular component biogenesis | 19 | 2619 | 0.052 |
| GO:0006793 | P | phosphorus metabolic process | 50 | 15620 | 1 |
| GO:0016705 | F | oxidoreductase activity, acting on paired donors, with incorporation or reduction of molecular oxygen | 38 | 3282 | 1.00E-06 |
| GO:0020037 | F | heme binding | 37 | 3260 | 2.20E-06 |
| GO:0005506 | F | iron ion binding | 39 | 3588 | 3.20E-06 |
| GO:0046906 | F | tetrapyrrole binding | 37 | 3528 | 1.20E-05 |
| GO:0019104 | F | DNA N-glycosylase activity | 5 | 60 | 1.60E-05 |
| GO:0003677 | F | DNA binding | 75 | 9698 | 3.70E-05 |
| GO:0004332 | F | fructose-bisphosphate aldolase activity | 5 | 102 | 0.00017 |
| GO:0016832 | F | aldehyde-lyase activity | 5 | 130 | 0.0005 |
| GO:0016799 | F | hydrolase activity, hydrolyzing N-glycosyl compounds | 5 | 129 | 0.00048 |
| GO:0042802 | F | identical protein binding | 6 | 223 | 0.00087 |
| GO:0016491 | F | oxidoreductase activity | 83 | 12358 | 0.0012 |
| GO:0016798 | F | hydrolase activity, acting on glycosyl bonds | 31 | 3571 | 0.0014 |
| GO:0008374 | F | O-acyltransferase activity | 6 | 269 | 0.0022 |
| GO:0016747 | F | transferase activity, transferring acyl groups other than amino-acyl groups | 16 | 1445 | 0.0021 |
| GO:0016746 | F | transferase activity, transferring acyl groups | 20 | 2002 | 0.002 |
| GO:0008415 | F | acyltransferase activity | 10 | 680 | 0.002 |
| GO:0019901 | F | protein kinase binding | 6 | 310 | 0.0043 |
| GO:0019900 | F | kinase binding | 6 | 310 | 0.0043 |
| GO:0004185 | F | serine-type carboxypeptidase activity | 9 | 640 | 0.0044 |
| GO:0046914 | F | transition metal ion binding | 68 | 10265 | 0.0046 |
| GO:0008236 | F | serine-type peptidase activity | 17 | 1767 | 0.0061 |
| GO:0004091 | F | carboxylesterase activity | 10 | 794 | 0.0058 |
| GO:0017171 | F | serine hydrolase activity | 17 | 1767 | 0.0061 |
| GO:0070008 | F | serine-type exopeptidase activity | 9 | 682 | 0.0065 |
| GO:0004180 | F | carboxypeptidase activity | 9 | 691 | 0.007 |
| GO:0004857 | F | enzyme inhibitor activity | 6 | 345 | 0.0071 |
| GO:0030599 | F | pectinesterase activity | 5 | 269 | 0.01 |
| GO:0004553 | F | hydrolase activity, hydrolyzing O-glycosyl compounds | 26 | 3355 | 0.013 |
| GO:0005507 | F | copper ion binding | 8 | 638 | 0.013 |
| GO:0043169 | F | cation binding | 90 | 15343 | 0.026 |
| GO:0008238 | F | exopeptidase activity | 9 | 863 | 0.025 |
| GO:0043167 | F | ion binding | 90 | 15344 | 0.026 |
| GO:0030528 | F | transcription regulator activity | 23 | 3116 | 0.03 |
| GO:0043565 | F | sequence-specific DNA binding | 15 | 1851 | 0.036 |
| GO:0060089 | F | molecular transducer activity | 8 | 789 | 0.039 |
| GO:0004871 | F | signal transducer activity | 8 | 789 | 0.039 |
| GO:0019899 | F | enzyme binding | 6 | 519 | 0.041 |
| GO:0009055 | F | electron carrier activity | 9 | 955 | 0.043 |
| GO:0016788 | F | hydrolase activity, acting on ester bonds | 27 | 3945 | 0.043 |
| GO:0046872 | F | metal ion binding | 87 | 15190 | 0.046 |
| GO:0016787 | F | hydrolase activity | 121 | 21923 | 0.051 |
| GO:0004252 | F | serine-type endopeptidase activity | 7 | 750 | 0.072 |
| GO:0070001 | F | aspartic-type peptidase activity | 5 | 529 | 0.11 |
| GO:0022857 | F | transmembrane transporter activity | 20 | 6212 | 0.98 |
| GO:0016740 | F | transferase activity | 112 | 28658 | 0.99 |
| GO:0016741 | F | transferase activity, transferring one-carbon groups | 12 | 2527 | 0.55 |
| GO:0016209 | F | antioxidant activity | 6 | 1149 | 0.47 |
| GO:0003682 | F | chromatin binding | 12 | 1546 | 0.073 |
| GO:0004386 | F | helicase activity | 9 | 2573 | 0.87 |
| GO:0043531 | F | ADP binding | 22 | 3362 | 0.091 |
| GO:0008324 | F | cation transmembrane transporter activity | 6 | 2580 | 0.98 |
| GO:0016818 | F | hydrolase activity, acting on acid anhydrides, in phosphorus-containing anhydrides | 22 | 7938 | 1 |
| GO:0016614 | F | oxidoreductase activity, acting on CH-OH group of donors | 7 | 1635 | 0.67 |
| GO:0016616 | F | oxidoreductase activity, acting on the CH-OH group of donors, NAD or NADP as acceptor | 6 | 1503 | 0.72 |
| GO:0005524 | F | ATP binding | 69 | 21555 | 1 |
| GO:0016758 | F | transferase activity, transferring hexosyl groups | 19 | 3493 | 0.32 |
| GO:0016830 | F | carbon-carbon lyase activity | 8 | 1109 | 0.17 |
| GO:0015631 | F | tubulin binding | 8 | 1090 | 0.16 |
| GO:0016817 | F | hydrolase activity, acting on acid anhydrides | 22 | 8171 | 1 |
| GO:0005488 | F | binding | 340 | 78438 | 1 |
| GO:0003676 | F | nucleic acid binding | 81 | 18400 | 0.8 |
| GO:0003678 | F | DNA helicase activity | 5 | 500 | 0.095 |
| GO:0008168 | F | methyltransferase activity | 12 | 2504 | 0.54 |
| GO:0042623 | F | ATPase activity, coupled | 5 | 2910 | 1 |
| GO:0008017 | F | microtubule binding | 8 | 1090 | 0.16 |
| GO:0017076 | F | purine nucleotide binding | 101 | 28306 | 1 |
| GO:0016684 | F | oxidoreductase activity, acting on peroxide as acceptor | 6 | 1097 | 0.43 |
| GO:0003774 | F | motor activity | 7 | 1259 | 0.4 |
| GO:0003777 | F | microtubule motor activity | 7 | 981 | 0.19 |
| GO:0003824 | F | catalytic activity | 350 | 73079 | 0.5 |
| GO:0016779 | F | nucleotidyltransferase activity | 5 | 2721 | 1 |
| GO:0022892 | F | substrate-specific transporter activity | 13 | 4138 | 0.96 |
| GO:0016772 | F | transferase activity, transferring phosphorus-containing groups | 53 | 19025 | 1 |
| GO:0015075 | F | ion transmembrane transporter activity | 11 | 3323 | 0.92 |
| GO:0032555 | F | purine ribonucleotide binding | 94 | 27034 | 1 |
| GO:0046983 | F | protein dimerization activity | 9 | 1780 | 0.48 |
| GO:0016757 | F | transferase activity, transferring glycosyl groups | 20 | 4235 | 0.55 |
| GO:0032553 | F | ribonucleotide binding | 94 | 27034 | 1 |
| GO:0000166 | F | nucleotide binding | 102 | 29819 | 1 |
| GO:0016706 | F | oxidoreductase activity, acting on paired donors, with incorporation or reduction of molecular oxygen, 2-oxoglutarate as one donor, and incorporation of one atom each of oxygen into both donors | 6 | 767 | 0.17 |
| GO:0016874 | F | ligase activity | 6 | 3092 | 1 |
| GO:0016829 | F | lyase activity | 13 | 2375 | 0.35 |
| GO:0008270 | F | zinc ion binding | 20 | 5778 | 0.95 |
| GO:0016301 | F | kinase activity | 47 | 15659 | 1 |
| GO:0005509 | F | calcium ion binding | 9 | 1474 | 0.28 |
| GO:0015297 | F | antiporter activity | 5 | 1166 | 0.66 |
| GO:0015291 | F | secondary active transmembrane transporter activity | 7 | 1320 | 0.44 |
| GO:0008233 | F | peptidase activity | 24 | 4270 | 0.24 |
| GO:0005215 | F | transporter activity | 49 | 8389 | 0.09 |
| GO:0016853 | F | isomerase activity | 7 | 1468 | 0.55 |
| GO:0030554 | F | adenyl nucleotide binding | 98 | 26095 | 1 |
| GO:0016773 | F | phosphotransferase activity, alcohol group as acceptor | 47 | 15564 | 1 |
| GO:0004190 | F | aspartic-type endopeptidase activity | 5 | 529 | 0.11 |
| GO:0005515 | F | protein binding | 97 | 21247 | 0.7 |
| GO:0016835 | F | carbon-oxygen lyase activity | 5 | 810 | 0.35 |
| GO:0051536 | F | iron-sulfur cluster binding | 5 | 564 | 0.14 |
| GO:0008092 | F | cytoskeletal protein binding | 9 | 1429 | 0.25 |
| GO:0004175 | F | endopeptidase activity | 13 | 2097 | 0.21 |
| GO:0016887 | F | ATPase activity | 8 | 4231 | 1 |
| GO:0030246 | F | carbohydrate binding | 6 | 917 | 0.28 |
| GO:0001883 | F | purine nucleoside binding | 98 | 26095 | 1 |
| GO:0001882 | F | nucleoside binding | 98 | 26393 | 1 |
| GO:0050662 | F | coenzyme binding | 12 | 2355 | 0.45 |
| GO:0050660 | F | FAD binding | 6 | 843 | 0.22 |
| GO:0051540 | F | metal cluster binding | 5 | 564 | 0.14 |
| GO:0016462 | F | pyrophosphatase activity | 22 | 7823 | 1 |
| GO:0005198 | F | structural molecule activity | 6 | 3354 | 1 |
| GO:0070011 | F | peptidase activity, acting on L-amino acid peptides | 24 | 4114 | 0.19 |
| GO:0004672 | F | protein kinase activity | 45 | 13823 | 1 |
| GO:0032559 | F | adenyl ribonucleotide binding | 91 | 24855 | 1 |
| GO:0022804 | F | active transmembrane transporter activity | 9 | 2735 | 0.91 |
| GO:0030234 | F | enzyme regulator activity | 10 | 1452 | 0.16 |
| GO:0017111 | F | nucleoside-triphosphatase activity | 22 | 7688 | 1 |
| GO:0016879 | F | ligase activity, forming carbon-nitrogen bonds | 5 | 1830 | 0.94 |
| GO:0022891 | F | substrate-specific transmembrane transporter activity | 12 | 3541 | 0.92 |
| GO:0003700 | F | transcription factor activity | 18 | 2732 | 0.11 |
| GO:0004601 | F | peroxidase activity | 6 | 1097 | 0.43 |
| GO:0048037 | F | cofactor binding | 16 | 3366 | 0.54 |
| GO:0034357 | C | photosynthetic membrane | 20 | 835 | 9.80E-09 |
| GO:0009521 | C | photosystem | 20 | 788 | 3.80E-09 |
| GO:0009579 | C | thylakoid | 21 | 893 | 5.70E-09 |
| GO:0009522 | C | photosystem I | 9 | 113 | 9.00E-09 |
| GO:0044436 | C | thylakoid part | 11 | 280 | 2.10E-07 |
| GO:0009654 | C | oxygen evolving complex | 8 | 166 | 2.20E-06 |
| GO:0009523 | C | photosystem II | 11 | 407 | 6.80E-06 |
| GO:0009507 | C | chloroplast | 6 | 109 | 2.10E-05 |
| GO:0009536 | C | plastid | 6 | 111 | 2.30E-05 |
| GO:0019898 | C | extrinsic to membrane | 6 | 146 | 9.80E-05 |
| GO:0044427 | C | chromosomal part | 14 | 978 | 0.00036 |
| GO:0016020 | C | membrane | 127 | 19834 | 0.00039 |
| GO:0005694 | C | chromosome | 15 | 1156 | 0.00062 |
| GO:0000786 | C | nucleosome | 9 | 506 | 0.00093 |
| GO:0032993 | C | protein-DNA complex | 9 | 506 | 0.00093 |
| GO:0000785 | C | chromatin | 9 | 584 | 0.0024 |
| GO:0030312 | C | external encapsulating structure | 5 | 288 | 0.014 |
| GO:0005618 | C | cell wall | 5 | 288 | 0.014 |
| GO:0044464 | C | cell part | 201 | 37668 | 0.04 |
| GO:0005623 | C | cell | 201 | 37668 | 0.04 |
| GO:0005634 | C | nucleus | 41 | 6703 | 0.068 |
| GO:0031224 | C | intrinsic to membrane | 35 | 7781 | 0.67 |
| GO:0016021 | C | integral to membrane | 34 | 7655 | 0.69 |
| GO:0043234 | C | protein complex | 39 | 8713 | 0.69 |
| GO:0043231 | C | intracellular membrane-bounded organelle | 47 | 8850 | 0.25 |
| GO:0044428 | C | nuclear part | 6 | 2015 | 0.92 |
| GO:0044422 | C | organelle part | 37 | 7005 | 0.29 |
| GO:0043229 | C | intracellular organelle | 73 | 15014 | 0.46 |
| GO:0005622 | C | intracellular | 91 | 20824 | 0.84 |
| GO:0043227 | C | membrane-bounded organelle | 50 | 8922 | 0.14 |
| GO:0005875 | C | microtubule associated complex | 8 | 1104 | 0.16 |
| GO:0044430 | C | cytoskeletal part | 9 | 2193 | 0.72 |
| GO:0044425 | C | membrane part | 54 | 10360 | 0.28 |
| GO:0044444 | C | cytoplasmic part | 9 | 6500 | 1 |
| GO:0005871 | C | kinesin complex | 7 | 973 | 0.19 |
| GO:0005737 | C | cytoplasm | 13 | 8179 | 1 |
| GO:0043232 | C | intracellular non-membrane-bounded organelle | 26 | 6514 | 0.85 |
| GO:0032991 | C | macromolecular complex | 51 | 12676 | 0.92 |
| GO:0043228 | C | non-membrane-bounded organelle | 26 | 6514 | 0.85 |
| GO:0044424 | C | intracellular part | 89 | 20029 | 0.79 |
| GO:0015630 | C | microtubule cytoskeleton | 8 | 1554 | 0.47 |
| GO:0044446 | C | intracellular organelle part | 37 | 7005 | 0.29 |
| GO:0005856 | C | cytoskeleton | 9 | 2452 | 0.83 |
| GO:0043226 | C | organelle | 73 | 15014 | 0.46 |

*BG numbers represents number of genes in background or reference. P = biological process; F = molecular function; C = cellular components and Term = predicted function/process associated with a particular Accession number.

**Supplementary Table 12.** Enrichment of GO terms that represents down-regulated genes unique to 48 h upon *Fusarium* inoculations.

| GO term | Term type | Term | Number in input list | Number in BG/Ref | p-value |
| --- | --- | --- | --- | --- | --- |
| GO:0006468 | P | protein amino acid phosphorylation | 422 | 13679 | 1.30E-39 |
| GO:0043687 | P | post-translational protein modification | 437 | 14897 | 3.60E-36 |
| GO:0006464 | P | protein modification process | 450 | 16098 | 1.10E-32 |
| GO:0016310 | P | phosphorylation | 425 | 15144 | 4.80E-31 |
| GO:0006796 | P | phosphate metabolic process | 427 | 15620 | 6.90E-29 |
| GO:0006793 | P | phosphorus metabolic process | 427 | 15620 | 6.90E-29 |
| GO:0043412 | P | macromolecule modification | 451 | 17042 | 1.00E-27 |
| GO:0051704 | P | multi-organism process | 53 | 593 | 2.10E-22 |
| GO:0009875 | P | pollen-pistil interaction | 48 | 548 | 4.20E-20 |
| GO:0008037 | P | cell recognition | 48 | 548 | 4.20E-20 |
| GO:0048544 | P | recognition of pollen | 48 | 548 | 4.20E-20 |
| GO:0009856 | P | pollination | 48 | 548 | 4.20E-20 |
| GO:0000003 | P | reproduction | 48 | 603 | 1.50E-18 |
| GO:0022414 | P | reproductive process | 48 | 603 | 1.50E-18 |
| GO:0032501 | P | multicellular organismal process | 50 | 778 | 9.60E-16 |
| GO:0044267 | P | cellular protein metabolic process | 494 | 23103 | 8.00E-13 |
| GO:0007154 | P | cell communication | 54 | 1083 | 1.10E-12 |
| GO:0008152 | P | metabolic process | 1303 | 73359 | 3.60E-10 |
| GO:0019538 | P | protein metabolic process | 536 | 26987 | 4.80E-09 |
| GO:0055114 | P | oxidation reduction | 257 | 11488 | 1.80E-08 |
| GO:0010556 | P | regulation of macromolecule biosynthetic process | 142 | 5560 | 3.70E-08 |
| GO:0009889 | P | regulation of biosynthetic process | 142 | 5560 | 3.70E-08 |
| GO:0031326 | P | regulation of cellular biosynthetic process | 142 | 5560 | 3.70E-08 |
| GO:0006355 | P | regulation of transcription, DNA-dependent | 139 | 5448 | 5.40E-08 |
| GO:0051252 | P | regulation of RNA metabolic process | 139 | 5448 | 5.40E-08 |
| GO:0045449 | P | regulation of transcription | 139 | 5458 | 6.00E-08 |
| GO:0051171 | P | regulation of nitrogen compound metabolic process | 140 | 5568 | 1.10E-07 |
| GO:0019219 | P | regulation of nucleobase, nucleoside, nucleotide and nucleic acid metabolic process | 140 | 5568 | 1.10E-07 |
| GO:0080090 | P | regulation of primary metabolic process | 142 | 5734 | 2.00E-07 |
| GO:0042221 | P | response to chemical stimulus | 65 | 2032 | 2.00E-07 |
| GO:0031323 | P | regulation of cellular metabolic process | 143 | 6059 | 2.30E-06 |
| GO:0010468 | P | regulation of gene expression | 141 | 5958 | 2.40E-06 |
| GO:0016998 | P | cell wall macromolecule catabolic process | 13 | 165 | 5.00E-06 |
| GO:0060255 | P | regulation of macromolecule metabolic process | 142 | 6139 | 6.80E-06 |
| GO:0016567 | P | protein ubiquitination | 19 | 390 | 2.90E-05 |
| GO:0006979 | P | response to oxidative stress | 36 | 1064 | 3.30E-05 |
| GO:0032446 | P | protein modification by small protein conjugation | 19 | 397 | 3.60E-05 |
| GO:0044036 | P | cell wall macromolecule metabolic process | 13 | 204 | 4.10E-05 |
| GO:0070647 | P | protein modification by small protein conjugation or removal | 20 | 445 | 5.30E-05 |
| GO:0019222 | P | regulation of metabolic process | 143 | 6551 | 9.10E-05 |
| GO:0006855 | P | multidrug transport | 25 | 685 | 0.00017 |
| GO:0042493 | P | response to drug | 25 | 685 | 0.00017 |
| GO:0015893 | P | drug transport | 25 | 685 | 0.00017 |
| GO:0009072 | P | aromatic amino acid family metabolic process | 12 | 249 | 0.00088 |
| GO:0044260 | P | cellular macromolecule metabolic process | 666 | 38134 | 0.0011 |
| GO:0006026 | P | aminoglycan catabolic process | 8 | 125 | 0.0012 |
| GO:0006030 | P | chitin metabolic process | 8 | 125 | 0.0012 |
| GO:0006032 | P | chitin catabolic process | 8 | 125 | 0.0012 |
| GO:0006351 | P | transcription, DNA-dependent | 145 | 7146 | 0.0015 |
| GO:0006350 | P | transcription | 145 | 7152 | 0.0016 |
| GO:0032774 | P | RNA biosynthetic process | 145 | 7176 | 0.0018 |
| GO:0023052 | P | signaling | 73 | 3274 | 0.0027 |
| GO:0071554 | P | cell wall organization or biogenesis | 20 | 660 | 0.0055 |
| GO:0006022 | P | aminoglycan metabolic process | 8 | 164 | 0.0057 |
| GO:0046417 | P | chorismate metabolic process | 7 | 141 | 0.0086 |
| GO:0009073 | P | aromatic amino acid family biosynthetic process | 7 | 141 | 0.0086 |
| GO:0009266 | P | response to temperature stimulus | 8 | 179 | 0.0092 |
| GO:0009408 | P | response to heat | 8 | 179 | 0.0092 |
| GO:0050794 | P | regulation of cellular process | 170 | 8997 | 0.0095 |
| GO:0009308 | P | amine metabolic process | 58 | 2665 | 0.011 |
| GO:0003333 | P | amino acid transmembrane transport | 9 | 227 | 0.012 |
| GO:0006865 | P | amino acid transport | 9 | 227 | 0.012 |
| GO:0015837 | P | amine transport | 9 | 227 | 0.012 |
| GO:0043170 | P | macromolecule metabolic process | 718 | 42476 | 0.013 |
| GO:0016053 | P | organic acid biosynthetic process | 30 | 1233 | 0.016 |
| GO:0046394 | P | carboxylic acid biosynthetic process | 30 | 1233 | 0.016 |
| GO:0006568 | P | tryptophan metabolic process | 5 | 96 | 0.021 |
| GO:0006586 | P | indolalkylamine metabolic process | 5 | 96 | 0.021 |
| GO:0042434 | P | indole derivative metabolic process | 5 | 96 | 0.021 |
| GO:0042430 | P | indole and derivative metabolic process | 5 | 96 | 0.021 |
| GO:0050896 | P | response to stimulus | 100 | 5156 | 0.022 |
| GO:0000272 | P | polysaccharide catabolic process | 8 | 214 | 0.023 |
| GO:0009628 | P | response to abiotic stimulus | 10 | 301 | 0.025 |
| GO:0006536 | P | glutamate metabolic process | 5 | 101 | 0.025 |
| GO:0006633 | P | fatty acid biosynthetic process | 14 | 489 | 0.027 |
| GO:0044283 | P | small molecule biosynthetic process | 44 | 2069 | 0.032 |
| GO:0044092 | P | negative regulation of molecular function | 7 | 189 | 0.034 |
| GO:0043086 | P | negative regulation of catalytic activity | 7 | 189 | 0.034 |
| GO:0006520 | P | cellular amino acid metabolic process | 47 | 2251 | 0.036 |
| GO:0009607 | P | response to biotic stimulus | 5 | 116 | 0.041 |
| GO:0042546 | P | cell wall biogenesis | 7 | 199 | 0.043 |
| GO:0070882 | P | cellular cell wall organization or biogenesis | 7 | 199 | 0.043 |
| GO:0044238 | P | primary metabolic process | 886 | 53794 | 0.048 |
| GO:0050789 | P | regulation of biological process | 170 | 9518 | 0.051 |
| GO:0009611 | P | response to wounding | 5 | 154 | 0.1 |
| GO:0008104 | P | protein localization | 16 | 2110 | 1 |
| GO:0009311 | P | oligosaccharide metabolic process | 7 | 433 | 0.52 |
| GO:0044281 | P | small molecule metabolic process | 107 | 8653 | 1 |
| GO:0044282 | P | small molecule catabolic process | 8 | 991 | 0.99 |
| GO:0045454 | P | cell redox homeostasis | 10 | 550 | 0.37 |
| GO:0009966 | P | regulation of signal transduction | 6 | 481 | 0.77 |
| GO:0044275 | P | cellular carbohydrate catabolic process | 7 | 891 | 0.99 |
| GO:0055085 | P | transmembrane transport | 95 | 6293 | 0.67 |
| GO:0006073 | P | cellular glucan metabolic process | 8 | 746 | 0.9 |
| GO:0065007 | P | biological regulation | 182 | 10277 | 0.057 |
| GO:0046483 | P | heterocycle metabolic process | 20 | 2215 | 1 |
| GO:0019725 | P | cellular homeostasis | 11 | 575 | 0.3 |
| GO:0051056 | P | regulation of small GTPase mediated signal transduction | 6 | 464 | 0.74 |
| GO:0006767 | P | water-soluble vitamin metabolic process | 6 | 356 | 0.49 |
| GO:0006766 | P | vitamin metabolic process | 6 | 356 | 0.49 |
| GO:0015849 | P | organic acid transport | 9 | 322 | 0.075 |
| GO:0006952 | P | defense response | 10 | 362 | 0.067 |
| GO:0032787 | P | monocarboxylic acid metabolic process | 17 | 1113 | 0.58 |
| GO:0045184 | P | establishment of protein localization | 16 | 1981 | 1 |
| GO:0030163 | P | protein catabolic process | 9 | 1364 | 1 |
| GO:0043436 | P | oxoacid metabolic process | 68 | 3777 | 0.15 |
| GO:0046486 | P | glycerolipid metabolic process | 5 | 971 | 1 |
| GO:0046128 | P | purine ribonucleoside metabolic process | 5 | 302 | 0.52 |
| GO:0043648 | P | dicarboxylic acid metabolic process | 8 | 357 | 0.21 |
| GO:0006575 | P | cellular amino acid derivative metabolic process | 9 | 494 | 0.38 |
| GO:0009605 | P | response to external stimulus | 5 | 239 | 0.33 |
| GO:0016052 | P | carbohydrate catabolic process | 15 | 1105 | 0.75 |
| GO:0016051 | P | carbohydrate biosynthetic process | 17 | 1171 | 0.66 |
| GO:0043038 | P | amino acid activation | 18 | 1051 | 0.39 |
| GO:0043039 | P | tRNA aminoacylation | 18 | 1051 | 0.39 |
| GO:0019637 | P | organophosphate metabolic process | 5 | 1222 | 1 |
| GO:0019438 | P | aromatic compound biosynthetic process | 9 | 347 | 0.11 |
| GO:0007165 | P | signal transduction | 17 | 2146 | 1 |
| GO:0019320 | P | hexose catabolic process | 7 | 808 | 0.97 |
| GO:0022607 | P | cellular component assembly | 6 | 2022 | 1 |
| GO:0034645 | P | cellular macromolecule biosynthetic process | 197 | 14577 | 0.99 |
| GO:0042398 | P | cellular amino acid derivative biosynthetic process | 5 | 310 | 0.54 |
| GO:0042364 | P | water-soluble vitamin biosynthetic process | 6 | 356 | 0.49 |
| GO:0032940 | P | secretion by cell | 6 | 406 | 0.62 |
| GO:0006807 | P | nitrogen compound metabolic process | 244 | 18913 | 1 |
| GO:0009064 | P | glutamine family amino acid metabolic process | 5 | 184 | 0.17 |
| GO:0034660 | P | ncRNA metabolic process | 18 | 1964 | 1 |
| GO:0044264 | P | cellular polysaccharide metabolic process | 8 | 854 | 0.96 |
| GO:0032318 | P | regulation of Ras GTPase activity | 5 | 394 | 0.74 |
| GO:0044262 | P | cellular carbohydrate metabolic process | 44 | 3762 | 0.98 |
| GO:0032313 | P | regulation of Rab GTPase activity | 5 | 249 | 0.36 |
| GO:0006886 | P | intracellular protein transport | 10 | 1479 | 1 |
| GO:0006887 | P | exocytosis | 6 | 406 | 0.62 |
| GO:0051188 | P | cofactor biosynthetic process | 11 | 914 | 0.85 |
| GO:0016043 | P | cellular component organization | 12 | 4641 | 1 |
| GO:0065003 | P | macromolecular complex assembly | 5 | 1833 | 1 |
| GO:0033013 | P | tetrapyrrole metabolic process | 5 | 278 | 0.45 |
| GO:0006812 | P | cation transport | 30 | 2427 | 0.93 |
| GO:0065009 | P | regulation of molecular function | 13 | 1155 | 0.91 |
| GO:0065008 | P | regulation of biological quality | 15 | 1280 | 0.9 |
| GO:0034613 | P | cellular protein localization | 10 | 1571 | 1 |
| GO:0018130 | P | heterocycle biosynthetic process | 6 | 695 | 0.96 |
| GO:0006810 | P | transport | 176 | 12862 | 0.98 |
| GO:0006629 | P | lipid metabolic process | 68 | 4142 | 0.38 |
| GO:0009309 | P | amine biosynthetic process | 16 | 810 | 0.22 |
| GO:0000271 | P | polysaccharide biosynthetic process | 7 | 794 | 0.97 |
| GO:0006811 | P | ion transport | 36 | 3014 | 0.96 |
| GO:0050790 | P | regulation of catalytic activity | 13 | 1145 | 0.91 |
| GO:0019318 | P | hexose metabolic process | 14 | 1245 | 0.92 |
| GO:0032483 | P | regulation of Rab protein signal transduction | 5 | 249 | 0.36 |
| GO:0032482 | P | Rab protein signal transduction | 5 | 249 | 0.36 |
| GO:0043413 | P | macromolecule glycosylation | 11 | 624 | 0.4 |
| GO:0023060 | P | signal transmission | 21 | 2390 | 1 |
| GO:0009059 | P | macromolecule biosynthetic process | 197 | 14608 | 0.99 |
| GO:0051336 | P | regulation of hydrolase activity | 5 | 422 | 0.79 |
| GO:0008652 | P | cellular amino acid biosynthetic process | 16 | 674 | 0.075 |
| GO:0046903 | P | secretion | 6 | 406 | 0.62 |
| GO:0044271 | P | cellular nitrogen compound biosynthetic process | 24 | 1441 | 0.42 |
| GO:0046907 | P | intracellular transport | 10 | 1916 | 1 |
| GO:0044272 | P | sulfur compound biosynthetic process | 5 | 379 | 0.71 |
| GO:0070727 | P | cellular macromolecule localization | 10 | 1571 | 1 |
| GO:0006950 | P | response to stress | 73 | 4184 | 0.2 |
| GO:0009110 | P | vitamin biosynthetic process | 6 | 356 | 0.49 |
| GO:0033865 | P | nucleoside bisphosphate metabolic process | 5 | 201 | 0.22 |
| GO:0006518 | P | peptide metabolic process | 5 | 157 | 0.11 |
| GO:0006519 | P | cellular amino acid and derivative metabolic process | 51 | 2606 | 0.073 |
| GO:0006576 | P | cellular biogenic amine metabolic process | 5 | 232 | 0.31 |
| GO:0006631 | P | fatty acid metabolic process | 15 | 735 | 0.19 |
| GO:0006732 | P | coenzyme metabolic process | 18 | 981 | 0.29 |
| GO:0046578 | P | regulation of Ras protein signal transduction | 6 | 464 | 0.74 |
| GO:0044042 | P | glucan metabolic process | 8 | 746 | 0.9 |
| GO:0009312 | P | oligosaccharide biosynthetic process | 6 | 218 | 0.14 |
| GO:0044248 | P | cellular catabolic process | 23 | 2887 | 1 |
| GO:0044249 | P | cellular biosynthetic process | 261 | 18890 | 0.99 |
| GO:0034641 | P | cellular nitrogen compound metabolic process | 62 | 3316 | 0.1 |
| GO:0009250 | P | glucan biosynthetic process | 7 | 647 | 0.88 |
| GO:0023051 | P | regulation of signaling process | 6 | 481 | 0.77 |
| GO:0010646 | P | regulation of cell communication | 6 | 481 | 0.77 |
| GO:0043087 | P | regulation of GTPase activity | 5 | 419 | 0.79 |
| GO:0007265 | P | Ras protein signal transduction | 6 | 464 | 0.74 |
| GO:0043632 | P | modification-dependent macromolecule catabolic process | 9 | 1119 | 0.99 |
| GO:0006139 | P | nucleobase, nucleoside, nucleotide and nucleic acid metabolic process | 193 | 16571 | 1 |
| GO:0051234 | P | establishment of localization | 176 | 12862 | 0.98 |
| GO:0042278 | P | purine nucleoside metabolic process | 5 | 302 | 0.52 |
| GO:0005984 | P | disaccharide metabolic process | 6 | 370 | 0.53 |
| GO:0051641 | P | cellular localization | 16 | 2451 | 1 |
| GO:0051179 | P | localization | 176 | 12991 | 0.98 |
| GO:0043933 | P | macromolecular complex subunit organization | 7 | 2087 | 1 |
| GO:0015672 | P | monovalent inorganic cation transport | 5 | 1002 | 1 |
| GO:0016070 | P | RNA metabolic process | 169 | 10967 | 0.62 |
| GO:0006644 | P | phospholipid metabolic process | 5 | 1106 | 1 |
| GO:0008610 | P | lipid biosynthetic process | 26 | 1647 | 0.52 |
| GO:0051603 | P | proteolysis involved in cellular protein catabolic process | 9 | 1231 | 1 |
| GO:0006720 | P | isoprenoid metabolic process | 6 | 308 | 0.36 |
| GO:0009987 | P | cellular process | 954 | 64217 | 0.99 |
| GO:0019941 | P | modification-dependent protein catabolic process | 9 | 1119 | 0.99 |
| GO:0034637 | P | cellular carbohydrate biosynthetic process | 15 | 1093 | 0.74 |
| GO:0030243 | P | cellulose metabolic process | 6 | 330 | 0.42 |
| GO:0023046 | P | signaling process | 21 | 2390 | 1 |
| GO:0030001 | P | metal ion transport | 18 | 972 | 0.28 |
| GO:0006508 | P | proteolysis | 52 | 4923 | 1 |
| GO:0044257 | P | cellular protein catabolic process | 9 | 1231 | 1 |
| GO:0044255 | P | cellular lipid metabolic process | 27 | 2348 | 0.96 |
| GO:0055086 | P | nucleobase, nucleoside and nucleotide metabolic process | 14 | 1705 | 1 |
| GO:0033036 | P | macromolecule localization | 20 | 2573 | 1 |
| GO:0006082 | P | organic acid metabolic process | 69 | 3796 | 0.13 |
| GO:0007242 | P | intracellular signaling cascade | 9 | 1246 | 1 |
| GO:0005996 | P | monosaccharide metabolic process | 15 | 1329 | 0.93 |
| GO:0005992 | P | trehalose biosynthetic process | 5 | 163 | 0.12 |
| GO:0005991 | P | trehalose metabolic process | 5 | 177 | 0.15 |
| GO:0033692 | P | cellular polysaccharide biosynthetic process | 7 | 755 | 0.95 |
| GO:0051186 | P | cofactor metabolic process | 21 | 1258 | 0.42 |
| GO:0009108 | P | coenzyme biosynthetic process | 10 | 674 | 0.62 |
| GO:0006006 | P | glucose metabolic process | 9 | 968 | 0.97 |
| GO:0006007 | P | glucose catabolic process | 7 | 808 | 0.97 |
| GO:0044106 | P | cellular amine metabolic process | 47 | 2394 | 0.079 |
| GO:0016137 | P | glycoside metabolic process | 6 | 370 | 0.53 |
| GO:0046365 | P | monosaccharide catabolic process | 7 | 808 | 0.97 |
| GO:0016138 | P | glycoside biosynthetic process | 5 | 206 | 0.23 |
| GO:0019752 | P | carboxylic acid metabolic process | 68 | 3777 | 0.15 |
| GO:0030244 | P | cellulose biosynthetic process | 6 | 330 | 0.42 |
| GO:0042592 | P | homeostatic process | 11 | 919 | 0.85 |
| GO:0015031 | P | protein transport | 16 | 1981 | 1 |
| GO:0006091 | P | generation of precursor metabolites and energy | 8 | 2402 | 1 |
| GO:0009100 | P | glycoprotein metabolic process | 11 | 624 | 0.4 |
| GO:0009101 | P | glycoprotein biosynthetic process | 11 | 624 | 0.4 |
| GO:0006486 | P | protein amino acid glycosylation | 11 | 624 | 0.4 |
| GO:0008299 | P | isoprenoid biosynthetic process | 6 | 308 | 0.36 |
| GO:0010467 | P | gene expression | 182 | 14438 | 1 |
| GO:0006511 | P | ubiquitin-dependent protein catabolic process | 9 | 1119 | 0.99 |
| GO:0006790 | P | sulfur metabolic process | 5 | 440 | 0.82 |
| GO:0046351 | P | disaccharide biosynthetic process | 5 | 206 | 0.23 |
| GO:0046164 | P | alcohol catabolic process | 7 | 891 | 0.99 |
| GO:0006725 | P | cellular aromatic compound metabolic process | 19 | 815 | 0.065 |
| GO:0006418 | P | tRNA aminoacylation for protein translation | 17 | 977 | 0.37 |
| GO:0070085 | P | glycosylation | 11 | 624 | 0.4 |
| GO:0009058 | P | biosynthetic process | 287 | 20626 | 0.99 |
| GO:0044265 | P | cellular macromolecule catabolic process | 16 | 2278 | 1 |
| GO:0009117 | P | nucleotide metabolic process | 11 | 1512 | 1 |
| GO:0009116 | P | nucleoside metabolic process | 8 | 462 | 0.44 |
| GO:0051649 | P | establishment of localization in cell | 16 | 2322 | 1 |
| GO:0009119 | P | ribonucleoside metabolic process | 5 | 355 | 0.66 |
| GO:0007264 | P | small GTPase mediated signal transduction | 9 | 1048 | 0.98 |
| GO:0009056 | P | catabolic process | 38 | 3313 | 0.98 |
| GO:0009057 | P | macromolecule catabolic process | 24 | 2625 | 1 |
| GO:0046942 | P | carboxylic acid transport | 9 | 322 | 0.075 |
| GO:0006753 | P | nucleoside phosphate metabolic process | 11 | 1512 | 1 |
| GO:0005975 | P | carbohydrate metabolic process | 96 | 7421 | 0.98 |
| GO:0042180 | P | cellular ketone metabolic process | 68 | 3797 | 0.16 |
| GO:0005976 | P | polysaccharide metabolic process | 16 | 1107 | 0.67 |
| GO:0044237 | P | cellular metabolic process | 800 | 49113 | 0.12 |
| GO:0006066 | P | alcohol metabolic process | 18 | 1800 | 0.98 |
| GO:0006399 | P | tRNA metabolic process | 18 | 1619 | 0.95 |
| GO:0044085 | P | cellular component biogenesis | 13 | 2619 | 1 |
| GO:0006259 | P | DNA metabolic process | 7 | 3762 | 1 |
| GO:0006412 | P | translation | 31 | 4563 | 1 |
| GO:0006396 | P | RNA processing | 5 | 2286 | 1 |
| GO:0016192 | P | vesicle-mediated transport | 10 | 1653 | 1 |
| GO:0004672 | F | protein kinase activity | 422 | 13823 | 1.30E-38 |
| GO:0030246 | F | carbohydrate binding | 83 | 917 | 1.20E-34 |
| GO:0016773 | F | phosphotransferase activity, alcohol group as acceptor | 431 | 15564 | 2.50E-30 |
| GO:0016301 | F | kinase activity | 432 | 15659 | 4.60E-30 |
| GO:0016740 | F | transferase activity | 653 | 28658 | 6.30E-24 |
| GO:0030247 | F | polysaccharide binding | 42 | 463 | 2.80E-18 |
| GO:0001871 | F | pattern binding | 42 | 463 | 2.80E-18 |
| GO:0005506 | F | iron ion binding | 131 | 3588 | 1.20E-17 |
| GO:0016772 | F | transferase activity, transferring phosphorus-containing groups | 445 | 19025 | 1.90E-17 |
| GO:0020037 | F | heme binding | 120 | 3260 | 1.60E-16 |
| GO:0016705 | F | oxidoreductase activity, acting on paired donors, with incorporation or reduction of molecular oxygen | 119 | 3282 | 6.10E-16 |
| GO:0003824 | F | catalytic activity | 1335 | 73079 | 7.90E-15 |
| GO:0046872 | F | metal ion binding | 357 | 15190 | 3.50E-14 |
| GO:0046906 | F | tetrapyrrole binding | 120 | 3528 | 3.30E-14 |
| GO:0043167 | F | ion binding | 357 | 15344 | 1.30E-13 |
| GO:0043169 | F | cation binding | 357 | 15343 | 1.30E-13 |
| GO:0003700 | F | transcription factor activity | 99 | 2732 | 1.80E-13 |
| GO:0016757 | F | transferase activity, transferring glycosyl groups | 132 | 4235 | 7.10E-13 |
| GO:0046914 | F | transition metal ion binding | 255 | 10265 | 1.40E-12 |
| GO:0030528 | F | transcription regulator activity | 104 | 3116 | 5.00E-12 |
| GO:0005509 | F | calcium ion binding | 60 | 1474 | 1.60E-10 |
| GO:0016758 | F | transferase activity, transferring hexosyl groups | 106 | 3493 | 5.50E-10 |
| GO:0016491 | F | oxidoreductase activity | 281 | 12358 | 7.80E-10 |
| GO:0043565 | F | sequence-specific DNA binding | 67 | 1851 | 1.50E-09 |
| GO:0005488 | F | binding | 1364 | 78438 | 3.30E-08 |
| GO:0048037 | F | cofactor binding | 93 | 3366 | 3.70E-07 |
| GO:0045735 | F | nutrient reservoir activity | 25 | 474 | 4.50E-07 |
| GO:0050662 | F | coenzyme binding | 71 | 2355 | 4.80E-07 |
| GO:0016747 | F | transferase activity, transferring acyl groups other than amino-acyl groups | 48 | 1445 | 2.90E-06 |
| GO:0004601 | F | peroxidase activity | 37 | 1097 | 2.80E-05 |
| GO:0016684 | F | oxidoreductase activity, acting on peroxide as acceptor | 37 | 1097 | 2.80E-05 |
| GO:0008066 | F | glutamate receptor activity | 11 | 145 | 3.60E-05 |
| GO:0015276 | F | ligand-gated ion channel activity | 11 | 145 | 3.60E-05 |
| GO:0022834 | F | ligand-gated channel activity | 11 | 145 | 3.60E-05 |
| GO:0005230 | F | extracellular ligand-gated ion channel activity | 11 | 145 | 3.60E-05 |
| GO:0005231 | F | excitatory extracellular ligand-gated ion channel activity | 11 | 145 | 3.60E-05 |
| GO:0005234 | F | extracellular-glutamate-gated ion channel activity | 11 | 145 | 3.60E-05 |
| GO:0004970 | F | ionotropic glutamate receptor activity | 11 | 145 | 3.60E-05 |
| GO:0016209 | F | antioxidant activity | 37 | 1149 | 6.90E-05 |
| GO:0004888 | F | transmembrane receptor activity | 11 | 162 | 9.20E-05 |
| GO:0016706 | F | oxidoreductase activity, acting on paired donors, with incorporation or reduction of molecular oxygen, 2-oxoglutarate as one donor, and incorporation of one atom each of oxygen into both donors | 27 | 767 | 0.00016 |
| GO:0015238 | F | drug transmembrane transporter activity | 25 | 685 | 0.00017 |
| GO:0004872 | F | receptor activity | 11 | 174 | 0.00017 |
| GO:0005507 | F | copper ion binding | 23 | 638 | 0.00035 |
| GO:0008762 | F | UDP-N-acetylmuramate dehydrogenase activity | 10 | 166 | 0.00047 |
| GO:0004425 | F | indole-3-glycerol-phosphate synthase activity | 5 | 37 | 0.00047 |
| GO:0016746 | F | transferase activity, transferring acyl groups | 52 | 2002 | 0.00052 |
| GO:0005516 | F | calmodulin binding | 7 | 87 | 0.00067 |
| GO:0004190 | F | aspartic-type endopeptidase activity | 19 | 529 | 0.0011 |
| GO:0008107 | F | galactoside 2-alpha-L-fucosyltransferase activity | 6 | 70 | 0.0012 |
| GO:0004568 | F | chitinase activity | 8 | 125 | 0.0012 |
| GO:0031127 | F | alpha(1,2)-fucosyltransferase activity | 6 | 70 | 0.0012 |
| GO:0070001 | F | aspartic-type peptidase activity | 19 | 529 | 0.0011 |
| GO:0001883 | F | purine nucleoside binding | 468 | 26095 | 0.0012 |
| GO:0030554 | F | adenyl nucleotide binding | 468 | 26095 | 0.0012 |
| GO:0008061 | F | chitin binding | 5 | 48 | 0.0014 |
| GO:0005524 | F | ATP binding | 391 | 21555 | 0.0016 |
| GO:0008417 | F | fucosyltransferase activity | 7 | 105 | 0.0019 |
| GO:0015171 | F | amino acid transmembrane transporter activity | 9 | 173 | 0.0023 |
| GO:0005275 | F | amine transmembrane transporter activity | 9 | 173 | 0.0023 |
| GO:0050660 | F | FAD binding | 25 | 843 | 0.0028 |
| GO:0001882 | F | nucleoside binding | 468 | 26393 | 0.0028 |
| GO:0004842 | F | ubiquitin-protein ligase activity | 19 | 580 | 0.003 |
| GO:0032559 | F | adenyl ribonucleotide binding | 440 | 24855 | 0.0043 |
| GO:0019787 | F | small conjugating protein ligase activity | 19 | 603 | 0.0045 |
| GO:0004806 | F | triglyceride lipase activity | 13 | 357 | 0.0057 |
| GO:0005342 | F | organic acid transmembrane transporter activity | 9 | 206 | 0.0068 |
| GO:0046943 | F | carboxylic acid transmembrane transporter activity | 9 | 206 | 0.0068 |
| GO:0019842 | F | vitamin binding | 24 | 862 | 0.0069 |
| GO:0015297 | F | antiporter activity | 30 | 1166 | 0.0079 |
| GO:0016298 | F | lipase activity | 14 | 436 | 0.012 |
| GO:0060089 | F | molecular transducer activity | 21 | 789 | 0.017 |
| GO:0004871 | F | signal transducer activity | 21 | 789 | 0.017 |
| GO:0010181 | F | FMN binding | 8 | 209 | 0.021 |
| GO:0017076 | F | purine nucleotide binding | 485 | 28306 | 0.021 |
| GO:0015291 | F | secondary active transmembrane transporter activity | 31 | 1320 | 0.022 |
| GO:0000166 | F | nucleotide binding | 509 | 29819 | 0.023 |
| GO:0030170 | F | pyridoxal phosphate binding | 20 | 764 | 0.023 |
| GO:0070279 | F | vitamin B6 binding | 20 | 764 | 0.023 |
| GO:0022804 | F | active transmembrane transporter activity | 57 | 2735 | 0.024 |
| GO:0016829 | F | lyase activity | 49 | 2375 | 0.039 |
| GO:0005515 | F | protein binding | 365 | 21247 | 0.04 |
| GO:0050661 | F | NADP or NADPH binding | 13 | 478 | 0.045 |
| GO:0004867 | F | serine-type endopeptidase inhibitor activity | 6 | 161 | 0.047 |
| GO:0004866 | F | endopeptidase inhibitor activity | 7 | 206 | 0.049 |
| GO:0030414 | F | peptidase inhibitor activity | 7 | 206 | 0.049 |
| GO:0032553 | F | ribonucleotide binding | 455 | 27034 | 0.062 |
| GO:0032555 | F | purine ribonucleotide binding | 455 | 27034 | 0.062 |
| GO:0022836 | F | gated channel activity | 11 | 410 | 0.066 |
| GO:0016831 | F | carboxy-lyase activity | 17 | 717 | 0.069 |
| GO:0042802 | F | identical protein binding | 7 | 223 | 0.069 |
| GO:0004252 | F | serine-type endopeptidase activity | 12 | 750 | 0.52 |
| GO:0017171 | F | serine hydrolase activity | 19 | 1767 | 0.97 |
| GO:0003924 | F | GTPase activity | 7 | 874 | 0.98 |
| GO:0016818 | F | hydrolase activity, acting on acid anhydrides, in phosphorus-containing anhydrides | 57 | 7938 | 1 |
| GO:0016817 | F | hydrolase activity, acting on acid anhydrides | 57 | 8171 | 1 |
| GO:0004091 | F | carboxylesterase activity | 15 | 794 | 0.28 |
| GO:0015399 | F | primary active transmembrane transporter activity | 17 | 1242 | 0.75 |
| GO:0022892 | F | substrate-specific transporter activity | 61 | 4138 | 0.71 |
| GO:0016787 | F | hydrolase activity | 229 | 21923 | 1 |
| GO:0016788 | F | hydrolase activity, acting on ester bonds | 44 | 3945 | 0.99 |
| GO:0016779 | F | nucleotidyltransferase activity | 12 | 2721 | 1 |
| GO:0008415 | F | acyltransferase activity | 12 | 680 | 0.39 |
| GO:0015075 | F | ion transmembrane transporter activity | 47 | 3323 | 0.79 |
| GO:0015077 | F | monovalent inorganic cation transmembrane transporter activity | 11 | 1286 | 0.99 |
| GO:0008026 | F | ATP-dependent helicase activity | 6 | 1249 | 1 |
| GO:0000287 | F | magnesium ion binding | 18 | 1449 | 0.87 |
| GO:0016410 | F | N-acyltransferase activity | 9 | 388 | 0.17 |
| GO:0008324 | F | cation transmembrane transporter activity | 30 | 2580 | 0.96 |
| GO:0016853 | F | isomerase activity | 11 | 1468 | 1 |
| GO:0003735 | F | structural constituent of ribosome | 8 | 2854 | 1 |
| GO:0022857 | F | transmembrane transporter activity | 108 | 6212 | 0.16 |
| GO:0070011 | F | peptidase activity, acting on L-amino acid peptides | 42 | 4114 | 1 |
| GO:0004497 | F | monooxygenase activity | 7 | 276 | 0.15 |
| GO:0004499 | F | flavin-containing monooxygenase activity | 7 | 233 | 0.082 |
| GO:0004812 | F | aminoacyl-tRNA ligase activity | 18 | 1005 | 0.33 |
| GO:0016741 | F | transferase activity, transferring one-carbon groups | 14 | 2527 | 1 |
| GO:0008194 | F | UDP-glycosyltransferase activity | 21 | 1522 | 0.75 |
| GO:0004386 | F | helicase activity | 6 | 2573 | 1 |
| GO:0016759 | F | cellulose synthase activity | 6 | 306 | 0.35 |
| GO:0022891 | F | substrate-specific transmembrane transporter activity | 57 | 3541 | 0.45 |
| GO:0022890 | F | inorganic cation transmembrane transporter activity | 21 | 1703 | 0.89 |
| GO:0016879 | F | ligase activity, forming carbon-nitrogen bonds | 25 | 1830 | 0.79 |
| GO:0019001 | F | guanyl nucleotide binding | 17 | 2211 | 1 |
| GO:0015267 | F | channel activity | 13 | 565 | 0.12 |
| GO:0046983 | F | protein dimerization activity | 25 | 1780 | 0.74 |
| GO:0016798 | F | hydrolase activity, acting on glycosyl bonds | 54 | 3571 | 0.63 |
| GO:0004857 | F | enzyme inhibitor activity | 8 | 345 | 0.19 |
| GO:0016701 | F | oxidoreductase activity, acting on single donors with incorporation of molecular oxygen | 6 | 294 | 0.32 |
| GO:0016709 | F | oxidoreductase activity, acting on paired donors, with incorporation or reduction of molecular oxygen, NADH or NADPH as one donor, and incorporation of one atom of oxygen | 7 | 233 | 0.082 |
| GO:0016702 | F | oxidoreductase activity, acting on single donors with incorporation of molecular oxygen, incorporation of two atoms of oxygen | 6 | 251 | 0.21 |
| GO:0032561 | F | guanyl ribonucleotide binding | 15 | 2179 | 1 |
| GO:0016876 | F | ligase activity, forming aminoacyl-tRNA and related compounds | 18 | 1052 | 0.4 |
| GO:0016667 | F | oxidoreductase activity, acting on sulfur group of donors | 7 | 459 | 0.58 |
| GO:0005215 | F | transporter activity | 134 | 8389 | 0.44 |
| GO:0005216 | F | ion channel activity | 13 | 565 | 0.12 |
| GO:0008135 | F | translation factor activity, nucleic acid binding | 6 | 583 | 0.89 |
| GO:0008483 | F | transaminase activity | 5 | 217 | 0.26 |
| GO:0016407 | F | acetyltransferase activity | 9 | 400 | 0.19 |
| GO:0004175 | F | endopeptidase activity | 32 | 2097 | 0.59 |
| GO:0003676 | F | nucleic acid binding | 195 | 18400 | 1 |
| GO:0003677 | F | DNA binding | 159 | 9698 | 0.31 |
| GO:0008047 | F | enzyme activator activity | 5 | 505 | 0.9 |
| GO:0017111 | F | nucleoside-triphosphatase activity | 55 | 7688 | 1 |
| GO:0016903 | F | oxidoreductase activity, acting on the aldehyde or oxo group of donors | 6 | 447 | 0.7 |
| GO:0015082 | F | di-, tri-valent inorganic cation transmembrane transporter activity | 6 | 380 | 0.55 |
| GO:0016620 | F | oxidoreductase activity, acting on the aldehyde or oxo group of donors, NAD or NADP as acceptor | 5 | 352 | 0.65 |
| GO:0046873 | F | metal ion transmembrane transporter activity | 16 | 944 | 0.42 |
| GO:0005099 | F | Ras GTPase activator activity | 5 | 349 | 0.64 |
| GO:0005096 | F | GTPase activator activity | 5 | 494 | 0.89 |
| GO:0005097 | F | Rab GTPase activator activity | 5 | 349 | 0.64 |
| GO:0016760 | F | cellulose synthase (UDP-forming) activity | 6 | 306 | 0.35 |
| GO:0043531 | F | ADP binding | 51 | 3362 | 0.62 |
| GO:0004553 | F | hydrolase activity, hydrolyzing O-glycosyl compounds | 50 | 3355 | 0.67 |
| GO:0015405 | F | P-P-bond-hydrolysis-driven transmembrane transporter activity | 17 | 1242 | 0.75 |
| GO:0016881 | F | acid-amino acid ligase activity | 23 | 1224 | 0.23 |
| GO:0016887 | F | ATPase activity | 48 | 4231 | 0.99 |
| GO:0008168 | F | methyltransferase activity | 14 | 2504 | 1 |
| GO:0042626 | F | ATPase activity, coupled to transmembrane movement of substances | 16 | 1187 | 0.76 |
| GO:0042623 | F | ATPase activity, coupled | 25 | 2910 | 1 |
| GO:0046527 | F | glucosyltransferase activity | 8 | 986 | 0.99 |
| GO:0035251 | F | UDP-glucosyltransferase activity | 8 | 986 | 0.99 |
| GO:0005525 | F | GTP binding | 15 | 2179 | 1 |
| GO:0016835 | F | carbon-oxygen lyase activity | 18 | 810 | 0.098 |
| GO:0016836 | F | hydro-lyase activity | 8 | 392 | 0.28 |
| GO:0016830 | F | carbon-carbon lyase activity | 21 | 1109 | 0.23 |
| GO:0015300 | F | solute:solute antiporter activity | 5 | 481 | 0.87 |
| GO:0042578 | F | phosphoric ester hydrolase activity | 5 | 1086 | 1 |
| GO:0008238 | F | exopeptidase activity | 5 | 863 | 1 |
| GO:0008233 | F | peptidase activity | 44 | 4270 | 1 |
| GO:0008236 | F | serine-type peptidase activity | 19 | 1767 | 0.97 |
| GO:0016838 | F | carbon-oxygen lyase activity, acting on phosphates | 10 | 382 | 0.087 |
| GO:0004518 | F | nuclease activity | 5 | 989 | 1 |
| GO:0022838 | F | substrate-specific channel activity | 13 | 565 | 0.12 |
| GO:0010333 | F | terpene synthase activity | 8 | 341 | 0.18 |
| GO:0030234 | F | enzyme regulator activity | 15 | 1452 | 0.97 |
| GO:0016874 | F | ligase activity | 43 | 3092 | 0.81 |
| GO:0016875 | F | ligase activity, forming carbon-oxygen bonds | 18 | 1052 | 0.4 |
| GO:0009055 | F | electron carrier activity | 21 | 955 | 0.086 |
| GO:0015079 | F | potassium ion transmembrane transporter activity | 8 | 373 | 0.24 |
| GO:0008378 | F | galactosyltransferase activity | 8 | 321 | 0.14 |
| GO:0008270 | F | zinc ion binding | 99 | 5778 | 0.21 |
| GO:0008375 | F | acetylglucosaminyltransferase activity | 11 | 445 | 0.1 |
| GO:0030695 | F | GTPase regulator activity | 6 | 708 | 0.97 |
| GO:0016614 | F | oxidoreductase activity, acting on CH-OH group of donors | 26 | 1635 | 0.51 |
| GO:0016616 | F | oxidoreductase activity, acting on the CH-OH group of donors, NAD or NADP as acceptor | 24 | 1503 | 0.5 |
| GO:0051213 | F | dioxygenase activity | 6 | 280 | 0.28 |
| GO:0005083 | F | small GTPase regulator activity | 6 | 665 | 0.95 |
| GO:0070035 | F | purine NTP-dependent helicase activity | 6 | 1249 | 1 |
| GO:0022803 | F | passive transmembrane transporter activity | 13 | 565 | 0.12 |
| GO:0016769 | F | transferase activity, transferring nitrogenous groups | 5 | 217 | 0.26 |
| GO:0043492 | F | ATPase activity, coupled to movement of substances | 19 | 1448 | 0.81 |
| GO:0016462 | F | pyrophosphatase activity | 57 | 7823 | 1 |
| GO:0016820 | F | hydrolase activity, acting on acid anhydrides, catalyzing transmembrane movement of substances | 16 | 1399 | 0.92 |
| GO:0060589 | F | nucleoside-triphosphatase regulator activity | 6 | 748 | 0.98 |
| GO:0003723 | F | RNA binding | 11 | 3593 | 1 |
| GO:0003682 | F | chromatin binding | 26 | 1546 | 0.4 |
| GO:0005198 | F | structural molecule activity | 11 | 3354 | 1 |
| GO:0008080 | F | N-acetyltransferase activity | 9 | 383 | 0.16 |
| GO:0015035 | F | protein disulfide oxidoreductase activity | 7 | 348 | 0.31 |
| GO:0015036 | F | disulfide oxidoreductase activity | 7 | 348 | 0.31 |
| GO:0044421 | C | extracellular region part | 8 | 81 | 7.60E-05 |
| GO:0005615 | C | extracellular space | 7 | 66 | 0.00014 |
| GO:0005576 | C | extracellular region | 12 | 210 | 0.00021 |
| GO:0000151 | C | ubiquitin ligase complex | 19 | 593 | 0.0038 |
| GO:0044428 | C | nuclear part | 8 | 2015 | 1 |
| GO:0044424 | C | intracellular part | 114 | 20029 | 1 |
| GO:0044425 | C | membrane part | 100 | 10360 | 1 |
| GO:0044422 | C | organelle part | 20 | 7005 | 1 |
| GO:0005654 | C | nucleoplasm | 7 | 782 | 0.96 |
| GO:0031224 | C | intrinsic to membrane | 92 | 7781 | 1 |
| GO:0044464 | C | cell part | 381 | 37668 | 1 |
| GO:0070013 | C | intracellular organelle lumen | 7 | 900 | 0.99 |
| GO:0012505 | C | endomembrane system | 5 | 1115 | 1 |
| GO:0016021 | C | integral to membrane | 90 | 7655 | 1 |
| GO:0016020 | C | membrane | 268 | 19834 | 1 |
| GO:0043234 | C | protein complex | 50 | 8713 | 1 |
| GO:0043232 | C | intracellular non-membrane-bounded organelle | 12 | 6514 | 1 |
| GO:0043233 | C | organelle lumen | 7 | 900 | 0.99 |
| GO:0031090 | C | organelle membrane | 8 | 1440 | 1 |
| GO:0005783 | C | endoplasmic reticulum | 5 | 525 | 0.91 |
| GO:0031974 | C | membrane-enclosed lumen | 7 | 910 | 0.99 |
| GO:0043229 | C | intracellular organelle | 63 | 15014 | 1 |
| GO:0043228 | C | non-membrane-bounded organelle | 12 | 6514 | 1 |
| GO:0005623 | C | cell | 381 | 37668 | 1 |
| GO:0005622 | C | intracellular | 122 | 20824 | 1 |
| GO:0043227 | C | membrane-bounded organelle | 50 | 8922 | 1 |
| GO:0043226 | C | organelle | 63 | 15014 | 1 |
| GO:0000145 | C | exocyst | 6 | 228 | 0.16 |
| GO:0005938 | C | cell cortex | 6 | 238 | 0.18 |
| GO:0044446 | C | intracellular organelle part | 20 | 7005 | 1 |
| GO:0044444 | C | cytoplasmic part | 34 | 6500 | 1 |
| GO:0044448 | C | cell cortex part | 6 | 228 | 0.16 |
| GO:0005634 | C | nucleus | 37 | 6703 | 1 |
| GO:0030529 | C | ribonucleoprotein complex | 10 | 3457 | 1 |
| GO:0031981 | C | nuclear lumen | 7 | 835 | 0.98 |
| GO:0043231 | C | intracellular membrane-bounded organelle | 50 | 8850 | 1 |
| GO:0005737 | C | cytoplasm | 52 | 8179 | 1 |
| GO:0005840 | C | ribosome | 8 | 2853 | 1 |
| GO:0044451 | C | nucleoplasm part | 7 | 782 | 0.96 |
| GO:0032991 | C | macromolecular complex | 61 | 12676 | 1 |

*BG numbers represents number of genes in background or reference. P = biological process; F = molecular function; C = cellular components and Term = predicted function/process associated with a particular Accession number.
